# Development of a Large Gene-Associated SSR Marker Set and in-Depth Genetic Characterization in Scarlet Sage

**DOI:** 10.3389/fgene.2020.00504

**Published:** 2020-05-21

**Authors:** Si-Qian Jiao, Ai-Xiang Dong, Tian-Le Shi, Hui Liu, Ilga Porth, Hai-Bo Xin, Jian-Feng Mao

**Affiliations:** ^1^Beijing Advanced Innovation Center for Tree Breeding by Molecular Design, National Engineering Laboratory for Tree Breeding, Key Laboratory of Genetics and Breeding in Forest Trees and Ornamental Plants, Ministry of Education, College of Biological Sciences and Technology, Beijing Forestry University, Beijing, China; ^2^Beijing Key Laboratory of Greening Plants Breeding, Beijing Institute of Landscape Architecture, Beijing, China; ^3^Département des Sciences du Bois et de la Forêt, Pavillon Charles-Eugène-Marchand, Université Laval, Québec, QC, Canada

**Keywords:** *Salvia splendens*, genome-wide identification, gene-associated SSRs, genetic diversity, population structure

## Abstract

*Salvia splendens*, scarlet or tropical sage, is a tender perennial herbaceous flowering plant popularly grown in public and private gardens all over the world. In this study, we developed a set of simple sequence repeats (SSRs) from genome-wide sequences to assess the genetic diversity and population structure among 112 cultivars. We obtained 364,379 SSRs by mining scarlet sage’s recently published whole genome sequence; 14,545 gene-associated SSR loci were identified in 2 kb gene flanking regions. Among the 768 gene-associated SSR primer sets we screened, 576 loci successfully amplified in DNA pools of 3–4 different cultivars, of which 271 remained polymorphic when tested across eight individual plants. We searched for the related gene functions attributable to these gene-associated SSRs using diverse databases, resulting in 259 Non-redundant matching sequences, 205 individual Gene Ontology (GO) terms, 236 assigned to eukaryotic orthologous groups, and 67 KEGG-annotated (Kyoto Encyclopedia of Genes and Genomes) sequences. We finally selected 41 polymorphic SSR loci to infer genetic diversity and population structure among 112 *S. splendens* accessions. Based on the developed gene-associated SSRs, clustering analyses consistently revealed two distinct genetic groups within the core collection of *S. splendens* cultivars. This work developed and characterized an exhaustive set of genome-wide gene-associated SSR markers for scarlet sage. These SSRs can provide species identification, genetic diversity and population structure information for *S. splendens*, and will therefore be important tools for the management and protection of *S. splendens* germplasm.

## Introduction

*Salvia splendens* Ker-Gawler [National Center for Biotechnology Information (NCBI) taxon ID:180675], also known as scarlet sage or tropical sage, belongs to the Labiatae Salvia perennial herbs native to Brazil (Clebsch, 2003). While *S. splendens* is in fact a perennial of warmer climate zones, it is often grown in annual cultivation within cooler areas. When grown as an annual, it represents an important herbaceous flowering plant often used in parterre configurations. Due to its dense flowers, its wide variation of colors (scarlet, purple, pink, blue, lavender, salmon, yellow green, white, and bicolor), and long-lasting flowering (3–9 weeks or longer), scarlet sage is a very popular bedding plant widely cultivated in public gardens world-wide (Griffiths, 1994; Clebsch, 2003). *S. splendens* can provide outstanding visual effects when grown in beds, borders, and containers; further advantages include its long lifespan from late spring to the occurrences of first frosts and that its flower is easy to maintain and largely pest-free (Regnault-Roger, 1997). Scarlet sage’s demand on the market is ever increasing (Fu et al., 2009), and therefore breeding new varieties is also accelerating (Dong, 2009).

At present, Chinese breeders have screened new varieties suitable for domestic cultivation through introduction, trial planting, hybridization, and induced mutations (Wang et al., 2003; Hui et al., 2004; Fu et al., 2009; Hong et al., 2012; Liu K. et al., 2012; Liu X. et al., 2012). However, with the introduction of new excellent varieties, identities of many varieties remain obscure, with lack of genealogical background information, unclear species resources, which hampers effective breeding efforts (Tian et al., 2006). There are few studies on the genetics and genomics of *S. splendens*; for example, until recently, within the NCBI database only 33 gene sequences, mainly from the anthocyanin metabolic pathway, were available (Ge et al., 2014). Hence, it is now necessary to undertake genetic diversity studies of the existing *S. splendens* germplasm resources. So far, SSR markers are lacking for scarlet sage.

Simple sequence repeats (SSRs), otherwise known as short tandem repeats (STRs) or microsatellites, are abundant in eukaryotic and prokaryotic genomes, and are popular molecular markers in population genetics (Wang et al., 2018). Intra- and interspecific SSR length variation can be extensive, mainly due to the high DNA replication error rates for SSRs (Ellegren, 2004; Klintschar et al., 2004; Forster et al., 2015). Carefully selected SSRs behave as co-dominant genetic markers with high genetic polymorphism. Hence, SSRs are widely used as PCR-based markers in population characterizations as well as in genetic linkage mapping and tagging trait-associated genes during marker-assisted selection (Kumar et al., 2015). Despite growing competition from new markers such as SNPs (single nucleotide polymorphisms), SSRs still have great applicability due to their high polymorphism, relatively easy scoring, testable neutrality, Mendelian inheritance, and versatility and cost-effectiveness (Zane et al., 2002; Hodel et al., 2016). SSRs will remain as a convenient tool in population and pedigree investigations of many plant systems. Next-generation sequencing (NGS) has transformed the development of SSR loci for ecological and evolutionary biology studies. In recent years, high-throughput sequencing techniques have facilitated quick and inexpensive identification of large numbers of loci in non-model species (Hu et al., 2016), which paved the way for further genetic studies.

Exploring our recent high-quality genome assembly of *S. splendens* (Dong et al., 2018a), we quantified the SSRs distribution and developed a large, widely distributed set of polymorphic SSR markers from gene flanking regions, and provided a primary genetic characterization of scarlet sage cultivars or varieties. This study is therefore the first exhaustive report on the genetic diversity and population structure of *S. splendens*.

## Materials and Methods

### Sampling and Genomic DNA Isolation

Based on phenotypic assessment (flower color, plant height, inflorescence length and other indicators) within the *S. splendens* collection available to us, we selected 112 cultivars (such as Vista White, Oasis Red, Chilwee Lavender etc.) which originated from nine different sources (Supplementary Table S1). Phenotypic observations and sampling were performed on cultivated scarlet sage obtained at the Beijing Institute of Landscape Architecture germplasm bank and on plants growing in the green house. Among the selected samples, 57 accessions were hybrids from commercial cultivars provided by the Beijing Institute of Landscape Architecture (Beijing, China). Other samples originated from Floranova (England, United Kingdom) [16 cultivars], PanAmerican Seed Inc. (PanAmerican, United States) [12 cultivars], Syngenta Inc. (Holland) [9 cultivars], Beijing University of Agriculture (Beijing, China) [5 cultivars], XinYuan Seed Inc. (Beijing, China) [4 cultivars], Hangzhou Academy of Agricultural Sciences (Hangzhou, China) [3 cultivars], Takii Seed Inc. (Takii, Japan) [3 cultivars], and from a breeder in Chi Feng (Inner-Mongolia, China) [3 cultivars].

Fresh leaf tissue was collected from individual samples, flash frozen in liquid nitrogen and stored in a freezer at −80°C until the DNA was extracted. Genomic DNA was extracted using the common cetyltrimethyl ammonium bromide (CTAB) protocol established by Doyle and Doyle (1987). Briefly, 200 mg of frozen leaf tissue from each sample was ground in a 1.5 ml centrifugal tube under liquid nitrogen and by adding 800 μl of CTAB buffer to the homogenate. Samples were briefly mixed and incubated for 1 h at 55°C. A 24:1 mixture of chloroform:isoamyl alcohol was added to the homogenate, vortexed to mix, and centrifuged at 12,000 rpm for 10 min at room temperature. The supernatant for each sample was transferred to a new 1.5 ml centrifugal tube and mixed with one volume of isopropanol and incubated for 20 min at -20°C. Then, we collected the DNA-containing pellet by discarding the liquid, after centrifugation at 12,000 rpm for 10 min. The precipitate was washed twice with 500 μl ice-cold 70% ethanol. The ethanol was subsequently removed, and the resulting DNA pellet at the bottom of the tube was left to dry overnight. Finally, DNA was dissolved by adding 70 μl water. Concentration and purity of the resulting DNA solution were determined by Nano Drop 2000 (Thermo Scientific). DNA samples were diluted to a final concentration of 20 ng⋅μl^–1^ for further tests on PCR amplification using the newly developed SSR primers (see below).

### SSR Development and Genotyping

As a first step, we screened the entire *S. splendens* genome sequence (Dong et al., 2018a) for occurrence of SSRs with motif length of 2–9 bp and repeats more than 5. The *S. splendens* genome assembly was used as it was provided to GigaDB (Dong et al., 2018b) and available at ftp://parrot.genomics.cn/gigadb/pub/10.5524/100001_101000/100463/final_genome.fasta. We employed the genome-wide SSR analysis kit GMATA (Wang and Wang, 2016) to perform SSR mining and primer design. In the second step, we selected only such SSR loci that occurred within the 2 kb flanking regions of a gene. As these SSRs are tightly linked with genes, we defined these SSRs as gene-associated SSRs.

After obtaining results from the preliminary screening, we further selected SSR loci and designed primer pairs applying the following constraints: (1) 2–4 bp of motif length, (2) repeat number of 10–20, (3) amplification product size of 150–300 bp, (4) 20–22 bp primer lengths with forward and reverse primers of equal lengths, (5) annealing temperature of about 60°C. Finally, 768 SSR loci fulfilling these requirements for efficient high-throughput genotyping were selected to synthesize primers and were further considered for PCR amplification. Primer pairs were synthesized by Shanghai Sangon Biological Engineering Technology and Service Co., Ltd. (Shanghai, China).

The synthesized primer pairs were validated by PCR using the M13-tail technique, where a fluorescent label (FAM, HEX, TAMRA, ROX, respectively) is attached to a 5′M13-tailed forward primer (Boutin-Ganache et al., 2001). PCR amplification of each locus was conducted in a volume of 20 μl with 10 μl 2 × Taq PCR master mix (Biomed Tiangen, Beijing, China), 4 μl (4 pmol) fluorescent-dye-labeled M13 primer, 4 μl (4 pmol) reverse primer, and 2 μl (20 ng) genomic DNA. Conditions for PCR amplification were as follows: 94°C for 5 min; 28 cycles at 94°C for 40 s, annealing at 55°C for 40 s, and elongation at 72°C for 45 s; 10 cycles at 94°C for 40 s, annealing at 53°C for 40 s, elongation at 72°C for 45 s, with a final extension at 72°C for 10 min (Radosavljević et al., 2012). Labeled PCR products were analyzed on an ABI 3730XL sequencer (AB, United States) using the GeneScan-500 LIZ (ABI) size standard. SSR alleles were called with GeneMarker version 2.20 (Soft Genetics, State College, Pennsylvania, United States) and automated peaks binning was checked again manually. No SSR amplicons were sequenced here to confirm the presence of the intended SSR sequence in this study.

### Functional Assignments for Gene-Associated SSRs

Genes associated with SSR loci developed here were compared to protein databases entries, including the non-redundant (Nr) database^1^ and using BLASTX with a significance threshold *E*-value of 1e^–5^. For non-redundant annotations, BLAST2GO V. 2.4.4 was used to obtain Gene Ontology (GO) annotations of unique transcripts (Conesa et al., 2005). Metabolic pathway analysis of *S. splendens* was performed based on known pathways provided in the Kyoto Encyclopedia of Genes and Genomes (KEGG) (Kanehisa and Goto, 2000; Kanehisa et al., 2011). The gene sequences were also aligned with KOG (Eukaryotic Orthologous Groups) databases to predict and classify putative gene functions (Koonin et al., 2004).

### Data Analysis

Site-wise information on each SSR locus including number of alleles, number of effective alleles (*Ne*), observed heterozygosity (*Ho*), expected heterozygosity (*He*) were generated with GenALEx version 6.532 (Peakall and Smouse, 2012). Total number of alleles detected (*Nt*), number of available alleles per locus (*Na*), allelic richness (*AR*), number of private alleles by population (*Np*), observed (*Ho*), and expected (*He*) heterozygosity, and inbreeding coefficient (*F*) were estimated for each population using SPAGeDi (Hardy and Vekemans, 2002). Marker polymorphism indices including heterozygosity index (*H*), polymorphism information content (*PIC*), identification capability (*D*), effective multiple ratio (*E*), marker index (*MI*), and arithmetic mean heterozygosity (*H*_avp_) were calculated using iMEC software (iMEC) (Amiryousefi et al., 2018). Estimates of null allele frequencies (*Pn*) for each SSR locus and each population were obtained using FreeNA (Chapuis and Estoup, 2007). GeneCap version 1.434 was employed to identify samples sharing the same genotype or those with large allelic differences (Wilberg and Dreher, 2004). ARLEQUIN was used to test for Hardy-Weinberg Equilibrium (HWE), and for inference of the total genetic variation partitioned between and within populations based on Markov chain iterations and analysis of molecular variance (AMOVA) (Excoffier et al., 2007). The average pairwise level of genetic differentiation (*Fst*) between populations was measured by the GenALEx multilocus comparison method based on 999 permutations.

We used POPULATIONS software version 1.2.32^2^ to calculate *Dm*, the minimum genetic distance (Nei, 1987). The resulting neighbor-joining tree was viewed by FigTree (Rambaut, 2012) and branch support confidence was evaluated by bootstrapping with 1,000 replicates. We constructed the Neighbor-Net network which was displayed by SplitsTree (Huson and Bryant, 2006). Neighbor-Net is similar to the common Neighbor joining method, but by showing reticulations, it can represent alternative trees in the presence of distinct phylogenetic signals, which may arise, for instance, from gene flow between individual accessions.

In addition, model-based Bayesian analysis using STRUCTURE version 2.3.4 (Pritchard et al., 2000) was performed to evaluate genetic structuring among the 112 scarlet sage accessions. Structure analysis of the data (co-dominant markers), admixture, and independent allele frequency models were used in estimating the proper subgroups, without using species assignment of individuals as priors. For the analyses with the program STRUCTURE, we used a burn-in period of 100,000 iterations and *a posterior* numbers of Markov Chain Monte Carlo (MCMC) of 1,000,000 iterations for *K* equals 1–11. For each possible value of *K*, ten runs were performed. Two different approaches were used to detect the most likely *K* value: the first, proposed by Pritchard (Pritchard et al., 2000), is based on the rate of change of Ln P(D) for each *K* between 1 and 11, and the second is the criterion proposed by Evanno (Evanno et al., 2005), which is based on the second order rate of change of the likelihood function with respect to *K* (Δ*K*) (the *ad hoc* Δ*K* test). The results from STRUCTURE were processed with STRUCTURE HARVESTER (Earl and vonHoldt, 2012), which implements the Evanno method to identify the optimal groups (*K*). CLUMPAK (Kopelman et al., 2015) was used to visualize the barplot of the probability of membership from the results of the Q-matrix. Additionally, the discriminant analysis of principal components (DAPC), a multivariate method, was applied. When group priors are lacking, DAPC uses sequential K-means and model selection to infer genetic clusters. The function – “find clusters” – implemented in the “adegenet” package (Jombart, 2008) under R software (R Core Team, 2019) was carried out. The function “find clusters” runs successive K-means for a range of *k* values and computes the Bayesian information criterion (BIC) of the corresponding models. When we mapped the DAPC results, we added the label that provided the clustering result of the structure; finally we obtained the results graph with the R package, ggplot2 (Wickham, 2016).

## Results

### Distribution of SSR Loci and Their Motifs Across the *Salvia splendens* Genome

We documented a total of 364,379 SSRs with motif lengths of 2–9 bp throughout the *S. splendens* genome, translating to 451 SSRs/Mb (excluding mononucleotide SSRs) or an SSR every 2.2 kb. We did not further investigate mononucleotide repeats, complex SSRs or SSR motif lengths equal or greater 10 bp. Here we examined the types and distributions of these 364,379 SSRs in more detail.

**FIGURE 1:**
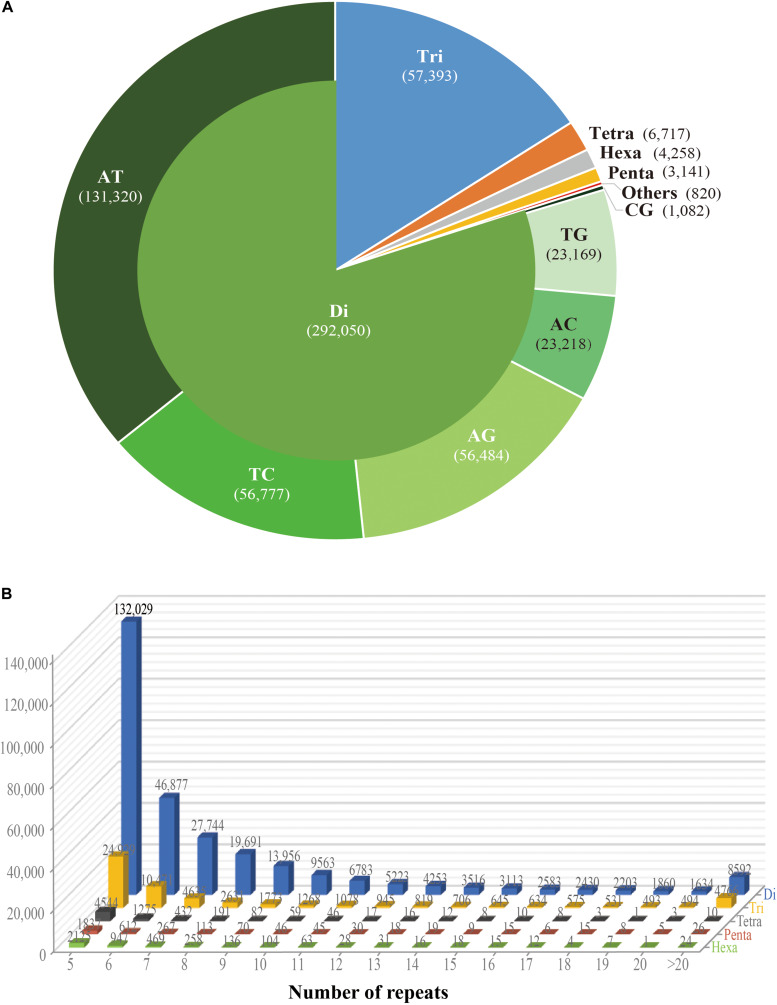
Number of different repeats and distribution patterns of SSR motif lengths obtained for the *Salvia splendens* genome sequence. **(A)** Number of different motif lengths of *S. splendens* and the distribution of different types of dinucleotide modes (AT; TC; AG; AC; TG; CG). **(B)** Distribution of SSR motif repeats from dinucleotides to hexanucleotides. The vertical axis represents the number of SSRs with different subject repetitions (from 5 to >20), which are distinguished by legends of different colors.

While the SSR frequency decreased as the number of repeat units increased, the rate of this change was more gradual for longer repeat motif types. Di- and tri-nucleotide repeats represented by far the major types of SSRs, accounting for 96%, and among these, di-nucleotide repeats were the most abundant type (80%; 292,050) of all detected SSRs. Tri-, tetra-, penta-, and hexa-nucleotide repeats accounted for 15.8% (57,393), 1.8% (6,717), 0.9% (3,141), and 1.2% (4,258), respectively. There were less than 3% (810) long-unit SSR loci (with motif lengths greater 6 bp) that we discovered in *S. splendens* (Table 1 and Figure 1A).

**TABLE 1:** Statistics for genome-wide retrieved SSRs in *Salvia splendens.*

Motif	Total	Percentage (%)	Density (SSRs/Mb)	Loci distance (kb)
Dinucleotide	292,050	80.15	361.45	2.77
Trinucleotide	57,393	15.75	71.03	14.08
Tetranucleotide	6,717	1.84	8.31	120.27
Hexanucleotide	3,141	0.86	3.89	257.24
Pentanucleotide	4,258	1.17	5.27	189.76
Other	820	0.23	1.01	985.37
Total	364,379	100	451.12	2.2

Overall, the frequency of SSRs decreased as the motif repeat number increased, and 89% (324,151) loci had fewer than 12 tandem repeats. In the distribution of di- to hexa-nucleotide SSR motif repeats in *S. splendens*, SSRs with five tandem repeats were the most frequent ones, with an abundance ranging from 132,029 to 2,125. Followed by six tandem repeats of SSRs were the second most common with a range of 46,877 to 947 in abundance. SSRs with more than 20 tandem repeats were very rare, with occurrences of 8,592 for di-nucleotide and 24 for penta-nucleotide motifs, for example (Figure 1B).

**FIGURE 2:**
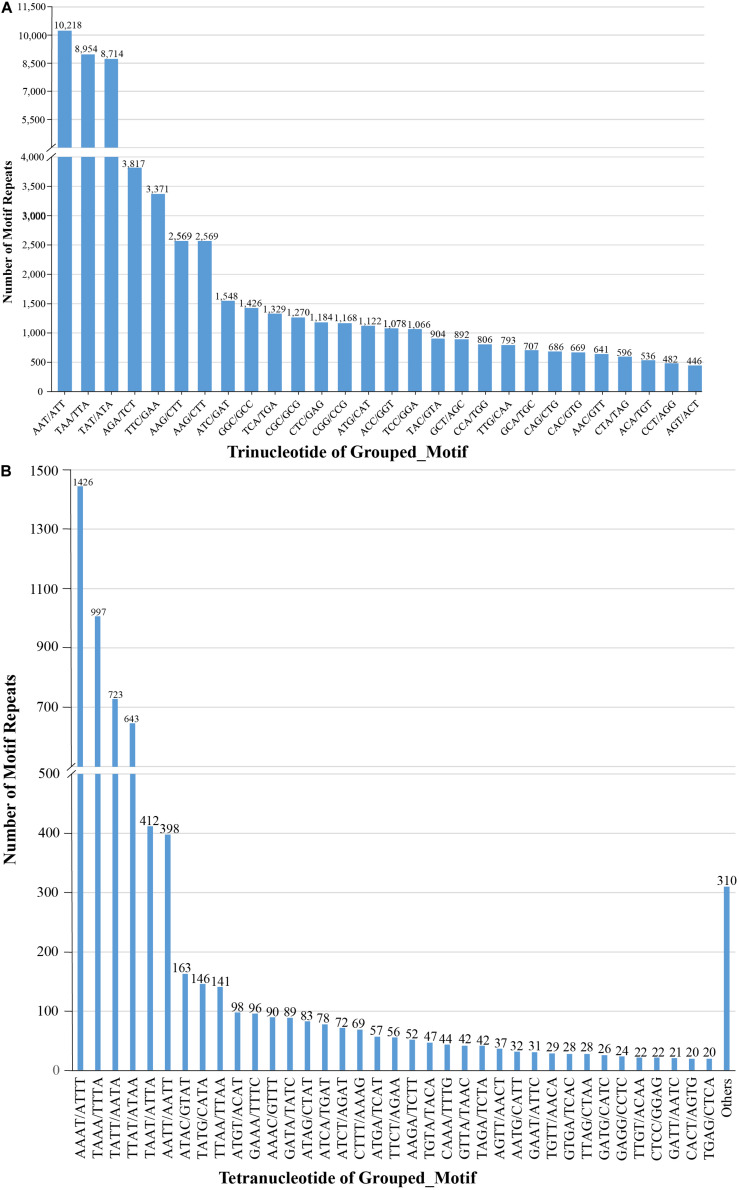
Number of tri- **(A)**, tetra- **(B)** nucleotide repeat motifs across the entire *S. splendens* genome.

We also examined the repeat motif types of the SSR. Motif AT/TA was the dominant di-nucleotide, accounting for 36% (131,320) of total SSRs in the *S. splendens* genome, followed by TC/GA (16.3%; 59,244), AG/CT (14.8%; 54,017), while the GC/GC motif pair was relatively rare (0.3%; 1,082) (Figure 1B). Motif pairs AAT/ATT, TAA/TTA, and TAT/ATA were the most abundant in tri-nucleotides repeats, with a total frequency of 8.2% (29,686) in the genome (Figure 2A). The most abundant tetra-nucleotide motif was AAAT/ATTT in the genome, with 0.4% frequency (1,426 in abundance) (Figure 2B).

### Gene-Associated SSR Marker Development and Polymorphism Assessment for *S. splendens*

We obtained 14,545 SSR loci located in the 2 kb range of the gene flanking regions. Finally, after strict filtering, we selected 768 SSR loci with 2–4 bp motif lengths and designed the primers for locus amplifications. SSR markers were screened using DNA pools of 3–4 different cultivars. 576 SSR markers were successfully amplified showing the expected size ranges. In the next step, 312 of the primer pairs generated monomorphic DNA amplified products (Supplementary Table S2), and 264 of the primer pairs exhibited allelic polymorphism, distinguishable by unambiguous banding patterns via capillary electrophoresis. Because seven primer pairs amplified more than one SSR locus, we could in fact detect 271 gene-associated SSRs with an amplicon of at least 100 bp in length (Supplementary Table S3). In total, amplified products from 271 primer pairs showed unambiguous and indisputably polymorphic bands. Di-nucleotide repeats were the most abundant (219 loci), followed by tri-nucleotide repeats (42 loci), and tetra-nucleotide repeats (three loci). Finally, the 271 SSR markers were used to evaluate the genetic diversity of 8 cultivars.

The SSR markers used in our evaluations (Supplementary Table S4) yielded a total of 800 alleles, the average number of alleles (*Na*) per locus was 2.952, ranging from 2 to 8. The mean value for number of effective alleles (*Ne*) was 2.197, with a range from 1.113 to 6.095. The observed heterozygosity (*Ho*) ranged from 0 to 1, with an average of 0.173, while the mean value of expected heterozygosity (*He*) was 0.475, and ranged from 0.011 to 0.836. The heterozygosity index (*H*) ranged from 0.664 to 0.219. The *PIC* values ranged from 0.594 to 0.372 with an average of 0.4394. The discriminating power (*D*) value was between 0.992 and 0.350 (Supplementary Table S4).

In addition, based on the results of the previous screening steps, we selected 41 polymorphic SSR markers for further genotyping of all 112 *S. splendens* cultivars in our collection (Supplementary Table S1), and a total of 485 different alleles was detected. The number of alleles per locus ranged between 4 and 29, with an average of 11.83 (Table 2). *He* ranged from 0.140 (locus ssps285) to 0.885 (ssps204), averaging at 0.348. *Ho* ranged from 0.028 (ssps285) to 0.964 (ssps343), averaging at 0.280. Zero null allele frequencies (*Pn*) were found at 20 SSR loci; the maximum values for *H* and *PIC* were 0.53 and 0.32, respectively, and their ranges 0.179 (ssps209) to 0.532 (ssps210) and 0.185 (ssps343) to 0.319 (ssps209), respectively. The *H*, *Hav* (arithmetic mean heterozygosity) and *MI* (marker index) values of each pair of primers were equal, indicating high ability in locus recognition and in distinction at the marker site, due to their high effective multiple ratio components. The *E* (effective multiple ratio) values were all 1, indicating that each assay reveals the corresponding locus for each marker. A higher *D* parameter (closest to 1) implied a lower probability of confusion between individual accessions. *D* parameters of 0.978 (ssps339) and 0.065 (ssps258) were considered highly and least polymorphic, respectively (Table 2).

**TABLE 2:** Characterization of 41 gene-associated SSR loci evaluated with 112 *Salvia splendens* individuals.

Locus	Allele Size Range (bp)	*N*	*Na*	*Ne*	*I*	*Ho*	*He*	*uHe*	*F*	*P*_n_	*Fst*	*Fst (ENA)*	*Fis*	*H*	*PIC*	*E*	*H.av*	*MI*	*D*
ssps196	147–173	107	7	2.778	1.289	0.047	0.640	0.643	0.927	0.000	0.378	0.288	0.866	0.317	0.299	1.000	0.317	0.317	0.261
ssps197	126–165	111	5	1.701	0.725	0.432	0.412	0.414	−0.049	0.000	−0.010	−0.005	−0.143	0.419	0.247	1.000	0.419	0.419	0.156
ssps198	201–270	100	15	5.175	2.012	0.340	0.807	0.811	0.579	0.100	0.147	0.126	0.554	0.321	0.304	1.000	0.321	0.321	0.301
ssps203	174–225	111	10	1.606	0.868	0.324	0.377	0.379	0.141	0.000	0.015	0.022	0.150	0.243	0.303	1.000	0.243	0.243	0.097
ssps204	150–236	102	20	8.677	2.440	0.167	0.885	0.889	0.812	0.080	0.059	0.045	0.749	0.254	0.312	1.000	0.254	0.254	0.249
ssps205	122–206	105	16	3.241	1.712	0.229	0.691	0.695	0.669	0.060	0.061	0.052	0.753	0.242	0.315	1.000	0.242	0.242	0.206
ssps207	231–291	103	12	3.006	1.405	0.728	0.667	0.671	−0.091	0.080	0.066	0.069	0.036	0.356	0.298	1.000	0.356	0.356	0.251
ssps209	122–196	109	21	4.207	1.967	0.514	0.762	0.766	0.326	0.000	0.015	0.008	0.370	0.179	0.319	1.000	0.179	0.179	0.138
ssps210	208–298	78	8	2.285	1.012	0.077	0.562	0.566	0.863	0.300	−0.025	−0.015	0.841	0.531	0.255	1.000	0.531	0.531	0.494
ssps211	221–305	93	29	7.740	2.520	0.258	0.871	0.876	0.704	0.160	0.011	0.011	0.697	0.339	0.290	1.000	0.339	0.339	0.336
ssps212	229–359	110	9	2.712	1.241	0.773	0.631	0.634	−0.224	0.000	−0.007	−0.006	−0.166	0.340	0.281	1.000	0.340	0.340	0.180
ssps224	151–231	98	15	5.090	2.001	0.765	0.804	0.808	0.048	0.120	0.032	0.026	0.138	0.378	0.297	1.000	0.378	0.378	0.334
ssps243	301–363	101	10	3.869	1.594	0.653	0.742	0.745	0.119	0.090	0.202	0.179	0.271	0.402	0.292	1.000	0.402	0.402	0.322
ssps244	217–267	102	9	2.892	1.290	0.608	0.654	0.657	0.071	0.080	0.027	0.039	0.143	0.406	0.289	1.000	0.406	0.406	0.309
ssps251	173–247	107	14	3.424	1.648	0.561	0.708	0.711	0.208	0.000	0.093	0.082	0.297	0.266	0.309	1.000	0.266	0.266	0.190
ssps255	180–213	107	4	1.176	0.347	0.121	0.149	0.150	0.187	0.000	0.005	0.008	0.339	0.454	0.258	1.000	0.454	0.454	0.154
ssps258	130–166	111	11	1.280	0.608	0.126	0.219	0.220	0.423	0.000	0.132	0.106	0.387	0.198	0.312	1.000	0.198	0.198	0.065
ssps272	234–260	104	12	2.257	1.220	0.269	0.557	0.560	0.517	0.070	0.172	0.172	0.663	0.296	0.308	1.000	0.296	0.296	0.219
ssps279	193–211	107	5	2.215	1.011	0.636	0.549	0.551	−0.159	0.000	−0.006	−0.005	−0.106	0.487	0.245	1.000	0.487	0.487	0.283
ssps285	227–235	107	4	1.163	0.311	0.028	0.140	0.141	0.800	0.000	0.013	0.051	0.904	0.434	0.265	1.000	0.434	0.434	0.155
ssps286	226–296	109	10	3.178	1.402	0.128	0.685	0.688	0.813	0.000	0.061	0.045	0.756	0.242	0.291	1.000	0.242	0.242	0.195
ssps293	283–313	107	10	4.485	1.742	0.822	0.777	0.781	−0.058	0.000	0.059	0.061	−0.083	0.357	0.271	1.000	0.357	0.357	0.281
ssps294	121–148	105	6	3.065	1.304	0.133	0.674	0.677	0.802	0.060	0.060	0.040	0.833	0.387	0.269	1.000	0.387	0.387	0.332
ssps296	172–226	111	12	3.549	1.567	0.189	0.718	0.721	0.737	0.000	−0.017	0.002	0.574	0.193	0.295	1.000	0.193	0.193	0.153
ssps299	236–282	106	8	2.839	1.358	0.132	0.648	0.651	0.796	0.000	0.292	0.207	0.784	0.319	0.283	1.000	0.319	0.319	0.262
ssps303	128–174	105	10	2.801	1.385	0.781	0.643	0.646	−0.214	0.060	0.021	0.021	−0.143	0.374	0.272	1.000	0.374	0.374	0.240
ssps304	172–262	99	12	3.900	1.671	0.424	0.744	0.747	0.429	0.110	0.119	0.111	0.597	0.369	0.280	1.000	0.369	0.369	0.322
ssps308	127–207	103	23	2.039	1.449	0.175	0.510	0.512	0.657	0.080	0.003	−0.005	0.577	0.230	0.297	1.000	0.230	0.230	0.193
ssps309	123–201	93	21	5.240	2.234	0.108	0.809	0.814	0.867	0.160	0.100	0.057	0.883	0.351	0.272	1.000	0.351	0.351	0.343
ssps314	266–324	97	10	3.254	1.464	0.691	0.693	0.696	0.003	0.130	0.103	0.089	0.121	0.443	0.269	1.000	0.443	0.443	0.337
ssps318	144–196	106	12	2.210	1.272	0.340	0.548	0.550	0.380	0.000	0.018	0.039	0.497	0.279	0.290	1.000	0.279	0.279	0.199
ssps321	157–238	106	16	2.784	1.404	0.755	0.641	0.644	−0.178	0.000	−0.004	−0.002	−0.259	0.276	0.291	1.000	0.276	0.276	0.168
ssps322	126–204	107	21	4.805	2.066	0.439	0.792	0.796	0.445	0.000	−0.006	−0.006	0.443	0.202	0.300	1.000	0.202	0.202	0.179
ssps325	242–293	92	15	2.942	1.616	0.261	0.660	0.664	0.605	0.170	0.069	0.053	0.622	0.397	0.268	1.000	0.397	0.397	0.365
ssps328	144–194	112	6	1.248	0.475	0.036	0.199	0.200	0.821	0.000	−0.011	−0.005	0.829	0.286	0.269	1.000	0.286	0.286	0.970
ssps330	121–196	110	12	1.669	1.005	0.045	0.401	0.403	0.887	0.000	0.051	0.040	0.902	0.189	0.298	1.000	0.189	0.189	0.104
ssps336	206–282	99	19	6.991	2.346	0.525	0.857	0.861	0.387	0.110	0.053	0.044	0.463	0.321	0.286	1.000	0.321	0.321	0.302
ssps339	256–310	112	7	1.746	0.846	0.054	0.427	0.429	0.875	0.000	−0.002	−0.002	0.850	0.256	0.278	1.000	0.256	0.256	0.978
ssps343	228–240	112	4	2.107	0.816	0.964	0.525	0.528	−0.835	0.000	0.022	0.022	−0.693	0.500	0.185	1.000	0.500	0.500	0.759
ssps344	289–297	108	4	1.525	0.607	0.056	0.344	0.346	0.839	0.000	0.017	0.034	0.783	0.430	0.244	1.000	0.430	0.430	0.232
ssps347	158–206	111	11	4.823	1.791	0.225	0.793	0.796	0.716	0.000	0.006	0.016	0.650	0.212	0.291	1.000	0.212	0.212	0.186
Mean		104.463	11.829	3.261	1.391	0.364	0.608	0.611	0.406	0.000				0.329	0.283	1.000	0.329	0.329	0.288
SE		1.070	0.917	0.271	0.087	0.043	0.031	0.031	0.066	0.000				0.093	0.025	0.000	0.093	0.093	0.197

****Na***, No. of Different Alleles; ***Ne***, No. of Effective Alleles; ***I***, Shannon’s Information Index; ***Ho***, Observed Heterozygosity; ***He***, Expected Heterozygosity; ***uHe***, Unbiased Expected Heterozygosity; ***F***, Fixation Index; ***H***, expected heterozygosity; ***PIC***, polymorphism information content; ***E***, effective multiplex ratio; ***Hav***, mean heterozygosity; ***MI***, marker index; ***D***, discriminating power.*

### Functional Annotations of the Gene-Associated SSRs in *S. splendens*

The 264 primer pairs (including the 271 surveyed polymorphic SSR loci, Supplementary Table S4) could be related to 259 individual genes. To predict the potential functions of the genes, we conducted annotations for all of these genes using three public domain databases, which yielded annotations for 205 (79%), 236 (91%), and 67 (26%) genes in GO, KOG, and KEGG databases, respectively.

**FIGURE 3:**
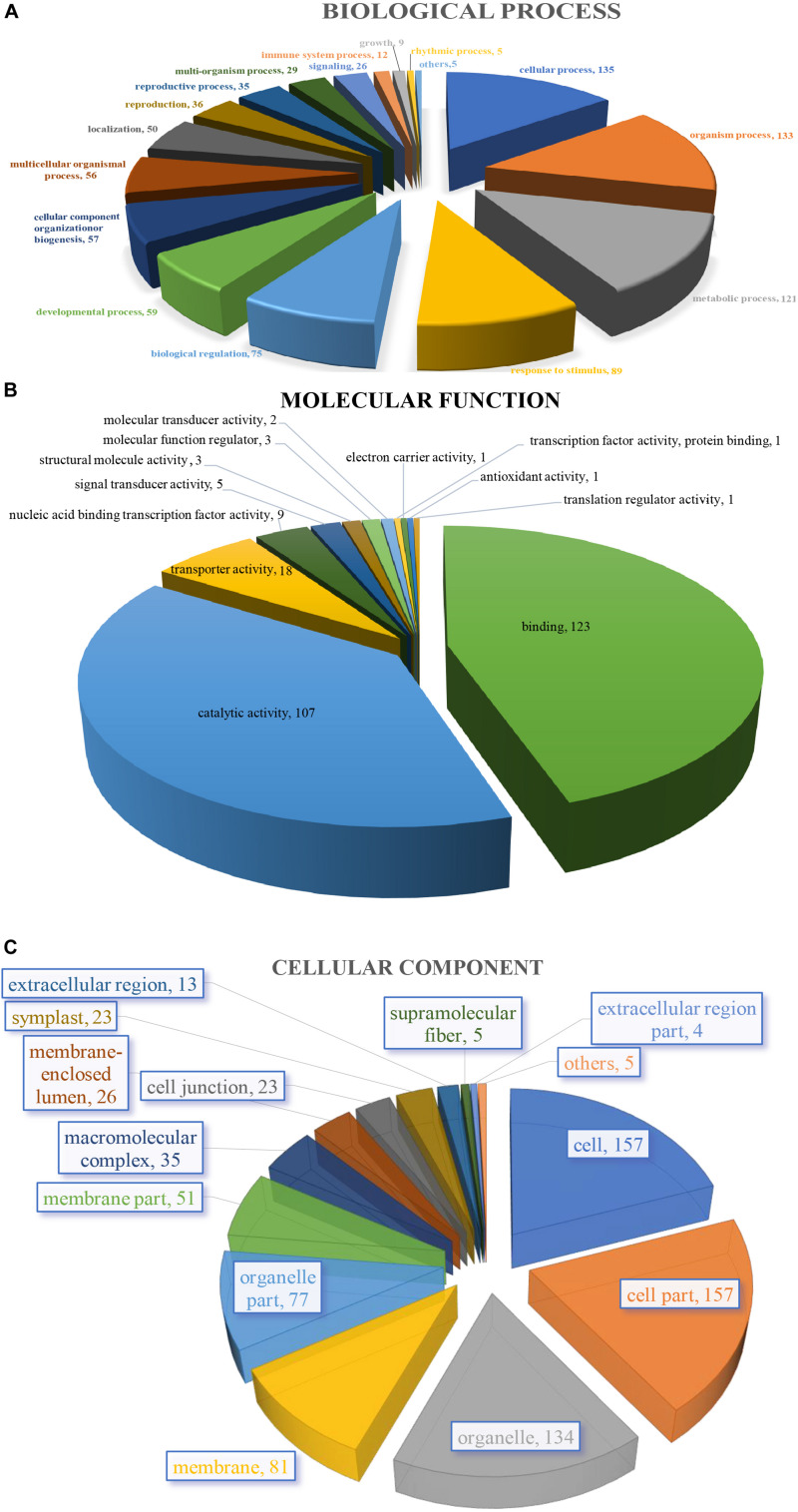
Characterization by gene ontology categories of 264 *S. splendens* gene-associated SSRs. **(A)** Biological process; **(B)** Molecular function; **(C)** Cellular component.

The 205 GO annotated genes were further categorized into 48 subcategories of the three main groups: biological process, cellular component, and molecular function (Figure 3). 168 genes were assigned in the biological process category as the dominant group [cellular process (135 genes); single-organism process (133); metabolic process (121) (Figure 3A)]. A total of 176 genes could be assigned to the molecular function category [binding (123 genes); catalytic activity (107); signal transducer activity (5) was least represented in the set (Figure 3B)]. For cellular component categorization, 172 genes were assigned to membrane, followed by cell (157), cell part (157), organelle (134), organelle part (77), membrane part (51), and extracellular region (13) (Figure 3C).

**FIGURE 4:**
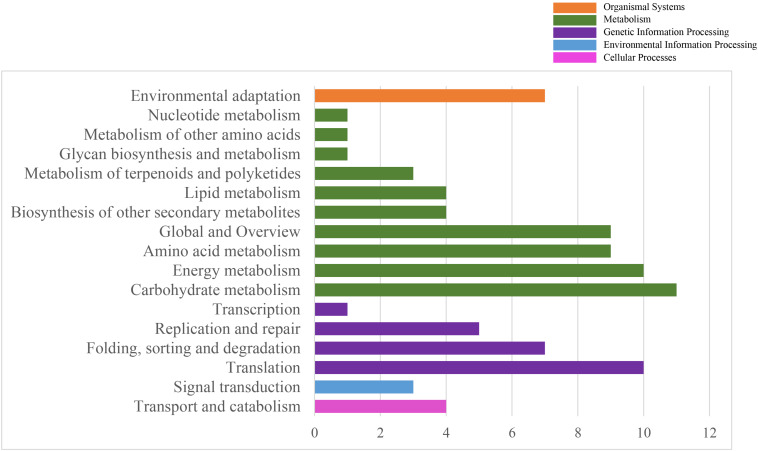
Characterization by KEGG categories of 67 *S. splendens* gene-associated SSRs.

To systematically understand the biological pathways in scarlet sage, a KEGG analysis was performed. Among the categorized loci, 67 genes with detected polymorphic SSRs could be related to 17 different subcategories within the KEGG database (Figure 4). We found five enriched KEGG categories for biochemical pathway, including cellular processes (4), environmental information processing (3), genetic information processing (23), metabolism (53), and organismal systems (7). In the subcategory “metabolism,” the most important were carbohydrate (11), energy (10), amino acid metabolism (9), and global and overview (9).

**FIGURE 5:**
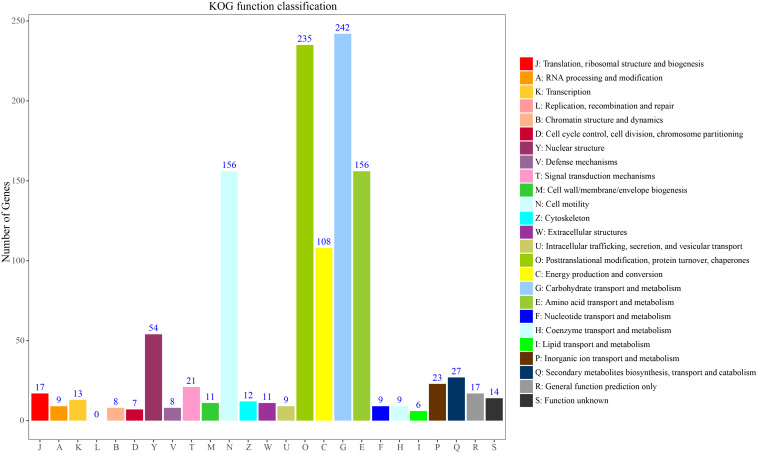
KOG function classification to characterize 236 *S. splendens* gene-associated SSRs.

To further classify these loci, 236 genes could be assigned to the 25 KOG functional categories A-Z (Figure 5). Most represented were primary metabolism, transport motility and synthesis as well as processing of DNA, RNA and proteins. Carbohydrate transport and metabolism (G) represented the largest group, followed by posttranslational modification, protein turnover, chaperones (O), amino acid transport and metabolism and cell motility (E). Fewer genes were assigned to energy production and conversion (C) and nuclear structure (Y). Overall, these functional annotations provide valuable information for gene-associated SSR exploration in *S. splendens*. Functional annotation for each gene-associated SSR is shown in Supplementary Table S5 for polymorphic SSRs and Supplementary Table S6 for monomorphic SSRs, respectively.

### *S. splendens* Cultivars Genetic Evaluation

**FIGURE 6:**
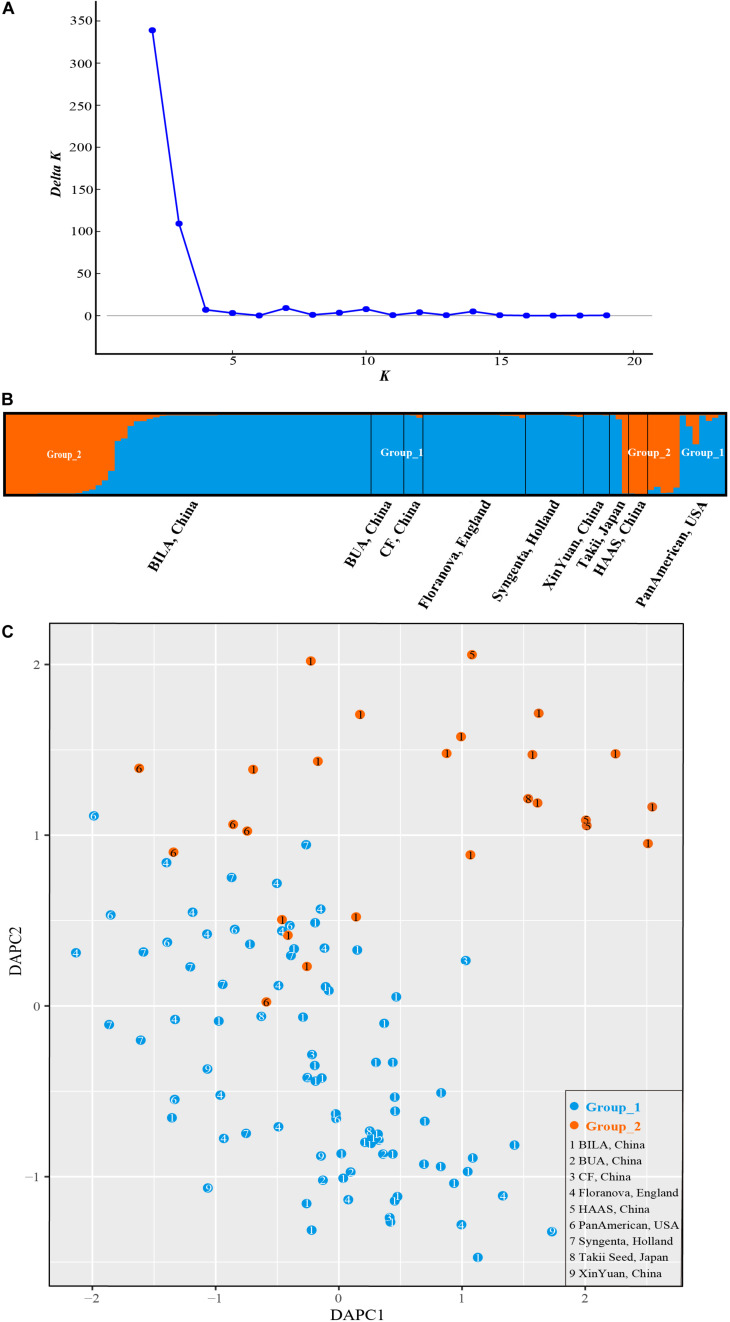
Bayesian clustering for SSR marker data from *S. splendens*. **(A)** Δ*K* was plotted against various values of *K*. **(B)** The two groups inferred from STRUCTURE analysis. **(C)** Discriminant analysis of principal components (DAPC) on SSR genotypic data. The number inside the points corresponds to the source of the varieties. Color of the point represents population assignment identified by STRUCTURE analysis.

STRUCTURE analysis revealed Δ*K*-peak at *K* = 2 as unambiguously the most likely number of groups (Figure 6A). Group_1 (in blue in Figure 6B) contained 86 accessions while Group_2 (orange, Figure 6B) had 26 cultivars. Genotypes in each cluster were categorized as pure or admixed, based on the estimated membership fractions. Accessions with an estimated membership fraction ≥0.80 were considered pure. Based on the genetic admixture, the accessions from BUA (Beijing University of Agriculture, China), CF (Chi Feng, China), Floranova (England), Syngenta (Holland), XinYuan (China), and HAAS (Hangzhou Academy of Agricultural Sciences, China) had no or negligible admixture, followed by BILA (Beijing Institute of Landscape Architecture, China), Takii (Japan), and PanAmerican (United States) which included some individuals with considerable admixture. The results from STRUCTURE failed to reveal any meaningful genetic structure or geographic grouping within our core collection of *S. splendens* stemming from nine different origins.

The sequential *k*-means algorithm in DAPC analysis also identified two groups (Figure 6C), consistent with previous results from STRUCTURE. We found individuals from BUA, CF, Floranova, Syngenta, and XinYuan assigned into Group_1 generated by STRUCTURE analysis, and those from HAAS were assigned to Group_2, respectively. Whereas BILA, Takii, and PanAmerican individuals divided among the two groups.

**FIGURE 7:**
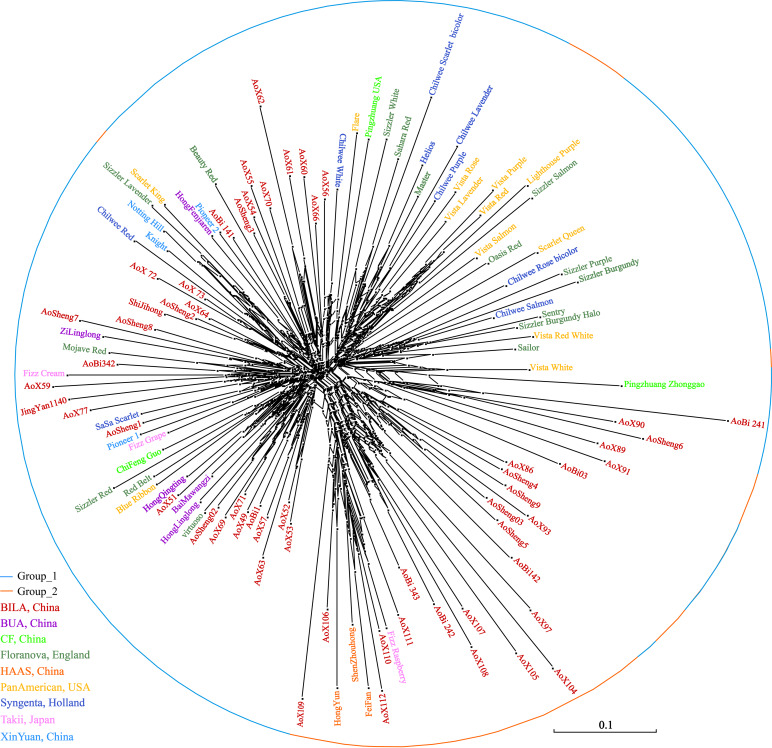
The neighbor network of *S. splendens* individuals.

The neighbor-network based on genetic distances among accessions presented a mixture (Figure 7). The 112 accessions roughly classified into two groups, corresponding to the previous overall assignment. Some samples, however, showed deviations from general clustering. For example, Aosheng5 and AoBi03 were assigned to Group_1 in STRUCTURE, but now, they were found inside Group_2 in the neighbor-network. The NJ tree (Supplementary Figure S1) generally concurred with the neighbor-network. In total, it was evident that genetic exchanges had been prevalent among these main *S. splendens* cultivars.

## Discussion

Genome-wide analysis of SSRs may reveal the potential functions of SSRs in gene regulation as well as genome organization, and serve as abundant markers for studies, such as population structure and genetic diversity analyses (Gol et al., 2017; Zhao et al., 2017). Based on the recently published *S. splendens* genome sequence (Dong et al., 2018a), we performed extensive data mining for SSR markers in the species and investigated the genetic diversity and population structure of the main cultivars in a core collection for this commercially important ornamental plant. This study was the first systematic population genetic study in *S. splendens* and we selected genetically unlinked genic markers and performed the first genetic evaluation for this commercially important ornamental plant with a long history of cultivation. At the same time, we developed a set of gene-associated SSR markers, which are effective tools for species identification and molecular breeding of *S. splendens*, thus providing a large number of molecular resources for further studies.

This large set of gene-associated SSRs (both, polymorphic and monomorphic loci) developed by us will be used as a new suit of genomic tools to study the various horticultural traits associated with this perennial herb. In our study, the distribution and frequency of SSRs was analyzed, focusing on repeat unit lengths of 2–9 bp in the *S. splendens* genome. A total of 364,379 SSRs were identified in the *S. splendens* genome (Supplementary Table S2). Given the estimated 808 Mb size of the entire *S. splendens* genome (Dong et al., 2018a), the overall SSR density was 451.12 SSRs per Mb. This observed SSR frequency in the *S. splendens* genome was lower than frequencies reported for other plant species. For example, overall densities of SSRs in genomes of rice (Zhang et al., 2007), poplar (Tuskan et al., 2006), and grapevine (Jaillon et al., 2007) were estimated as 529, 508, and 506 SSRs/Mb, respectively. This outcome may be mainly caused by the more stringent conditions used to define SSRs in the present study than those previously used for rice, poplar, and grapevine (Cavagnaro et al., 2010). However, it has been reported that there is a general negative correlation between genome size and SSR density in plants (Morgante et al., 2002), and our data in *S. splendens* would agree with this general trend.

Frequency analysis for the various motifs detected in scarlet sage showed that di-nucleotides and tri-nucleotides were the most abundant SSRs, accounting for up to 80.15% of all SSRs identified. This observation of highly abundant di-nucleotides and tri-nucleotides was in accordance with previous studies for several other species (Zhang et al., 2007; Liu et al., 2013; Xu et al., 2013). In addition, the repeat counts in SSR motifs showed that the distribution of genomic SSRs, irrespective of whether di-, tri-, tetra-, penta-, or hexa-nucleotides, tends to be biased to a smaller number of counts for each motif. While we also found a few cases of repeat numbers larger than 10 for di-nucleotide and tri-nucleotide SSRs, for tetra-, penta-, or hexa-nucleotide SSRs the frequency of high repeat counts was even lower in these longer motifs. The predominance of dinucleotides in *S. splendens* is in agreement with a previous SSR survey study in the species (Ge et al., 2014). For the scarlet sage genome, base composition of an SSR motif is strongly skewed toward A or T, especially at the first base. For instance, the most common di-nucleotide SSR motif was AT/TA, making up as much as 84% of all di-nucleotide motifs in the genome. In addition, a similar trend was also observed for longer motifs, such as AAT/ATT and AAAT/ATTT, which were the most abundant tri- and tetra-nucleotide motifs in *S. splendens* genome, respectively. The same pattern was observed in other annual and perennial plants (Gur-Arie et al., 2000; Venkateswarlu et al., 2006; Santonoceto et al., 2019; Tribhuvan et al., 2019), even though the most abundant repeat motifs in dinucleotide repeats can be different among plant species. Hence, some previous genomic studies revealed AC/CA to be the most common dinucleotide repeat instead (Cardle et al., 2000; Li et al., 2014). These different results may be attributable to specificities of the organisms that were studied (Tóth et al., 2000).

Among conventional DNA markers such as RAPD, AFLP and ISSR have been widely used while the application of single sequence repeats (SSRs) has been limited in *Salvia* (Karaca and Ince, 2017). We identified a set of high-quality markers to fill in the gap in the previous of *S. splendens* genetic assessment. Therefore, in our study, we screened 768 gene-associated SSR loci in *S. splendens*, of which 271 were polymorphic. Since the expected heterozygosity (*He*) ranged from 0.011 to 0.836, and the average *PIC* was 0.439, these values were close to those previously reported for closely related species. Nine SSR markers were developed from *Salvia officinalis* L., with the *Ho*, *He*, *PIC* ranged from 0.46 to 0.83, 0.73 to 0.93, and 0.70 to 0.92, respectively (Radosavljević et al., 2012). Similar level of genetic diversity was also seen for other *Salvia* species (ÝNce and Karaca, 2015; Krak et al., 2020). The SSR markers were identified to be highly polymorphic. These SSR markers could now be used to evaluate genetic similarity and interrelationship among cultivars. Also, the SSRs developed here are valuable tools for germplasm characterization, genetic diversity analysis, and will potentially support marker-assisted selection (MAS) in *S. splendens*.

In this study, we inferred the putative functions of the developed gene-associated SSR markers by performing a homology comparison of the genes containing the gene-associated SSRs from the gene annotation files. Here, these genes were found involved in a wide range of functions, which indicated that these gene-associated SSRs were potentially associated with important biological characters, since 79%, 91%, and 26% genes of *S. splendens* could be annotated with 48 GO subcategories, 25 KOG functional categories, and 17 different classifications in the KEGG database, respectively. GO assignments of SSR associated genes showed overrepresentation in categories related to regulating plant growth and primary metabolism. Regarding KOG, the majority of SSR loci developed were found to be associated with genes involved in primary metabolism, transport motility and synthesis as well as processing of DNA, RNA and proteins. Most of these SSR associated genes were enriched for metabolic process and environmental information processing in KEGG database. Similar functional enrichment results were also reported in date palm and citrus (Zhao et al., 2012; Liu et al., 2013). Hence, these results indicated that the developed gene-associated SSRs are not only significant for genetic diversity, but also valuable for future functional studies.

The 41 SSR markers selected in this study generated reproducible polymorphisms for all 112 cultivars, and helped to assess the genetic distances and the genetic structure within *S. splendens*. In our sample set of 112 cultivars, none of them were genetically identical. Using the core collection’s set of nine different origins, cultivars were classified into two large genetic groups. In this study, the results of the cluster analysis were not consistent with the collection’s origins. However, the results of STRUCTURE, DAPC, neighbor network and neighbor-joining analyses were consistent. Hybridization is currently the predominant way of scarlet sage breeding (Dong et al., 2012), such that in recent years, the *S. splendens* cultivation by crossing domestic with foreign varieties has been increasingly valued and welcomed in China, for example, the development of the two cultivars “Jingyan” and “Aoyunshenghuo” included in the present study. Here, we demonstrated that subtle hybridization history could be reconstructed with a larger sampling of cultivars, and using the developed SSR markers.

## Conclusion

In this study, we successfully developed and characterized a large set of genome-wide gene-associated SSR markers for scarlet sage. Simultaneously, this study constitutes the first report exploring the performance of SSR markers in the genetic analysis of cultivated *S. splendens* populations. In sum, we found that the SSRs used here are suitable for estimating genetic diversity and population structure in *S. splendens*. This resulting knowledge provides an important contribution to the breeding and conservation of *S. splendens* germplasm.

## Data Availability Statement

The raw data supporting the conclusions of this article will be made available by the authors, without undue reservation, to any qualified researcher.

## Author Contributions

J-FM, A-XD, and H-BX conceived and designed the experiments. S-QJ performed the experiment. A-XD and H-BX collected the samples. S-QJ, HL, and T-LS analyzed the data. S-QJ and J-FM wrote the manuscript. IP edited the manuscript.

## Conflict of Interest

The authors declare that the research was conducted in the absence of any commercial or financial relationships that could be construed as a potential conflict of interest.

## Footnotes

1 http://www.ncbi.nlm.nih.gov/

2 http://bioinformatics.org/∼tryphon/populations/
