# Supplementary material for: Development of a Large Gene-Associated SSR Marker Set and in-Depth Genetic Characterization in Scarlet Sage
Source: Front Genet. 2020 May 21;11:504. doi: 10.3389/fgene.2020.00504 (PMC7253628; doi:10.3389/fgene.2020.00504)
Supplement: Supplementary file 1 [file Data_Sheet_1.zip › Table S1-S4.DOCX]

**Table S1.** The 112 *S. splendens* accessions used in genetic analyses.

**Table S2.** Characteristics of 312 monomorphic SSR markers for *S. splendens*.

**Table S3.** Characteristics of 271 polymorphic SSR markers for *S. splendens*.

**Table S4.** Statistics calculated for 271 polymorphic SSR markers using eight *S. splendens* cultivar data sets.

**Table S5.** Functional annotations for the 264 polymorphic geneassociated SSRs found within the 2 kb gene flanking regions by querying the adjacent genes.

**Table S6.** Functional annotations for 312 monomorphic geneassociated SSRs in 2 kb gene flanking region retrieved by querying the adjacent genes.

**Table S1.** The 112 *S. splendens* accessions used in genetic analyses.

| **Sample No.** | **Name** | **Company/Country** | **Flower color** | **Plant height (cm)** | **Inflorescence length (cm)** |
| --- | --- | --- | --- | --- | --- |
| 1 | Vista White | PanAmerican Seed, United States of America | White | 14.4-15.6 | 12.3-20.7 |
| 2 | Vista Purple | PanAmerican Seed, United States of America | Purple | 14.6-17.2 | 10.5-16.5 |
| 3 | Vista Red | PanAmerican Seed, United States of America | Red | 16.9-20.0 | 16.1-19.0 |
| 4 | Vista Red white | PanAmerican Seed, United States of America | Flowerbed white corolla red | 18.4-25.0 | 15.1-18.3 |
| 5 | Vista Salmon | PanAmerican Seed, United States of America | Salmon red | 16.5-18.8 | 9.55-14.7 |
| 6 | Vista Rose | PanAmerican Seed, United States of America | Rose red | 13.2-14.9 | - |
| 7 | Vista Lavender | PanAmerican Seed, United States of America | Lavender | 17.7-20.0 | 18.9-21.3 |
| 8 | Scarlet King | PanAmerican Seed, United States of America | Red | 20.0-22.0 | 22.9-28.6 |
| 9 | Scarlet Queen | PanAmerican Seed, United States of America | Red | 21.0-25.0 | - |
| 10 | Flare | PanAmerican Seed, United States of America | Red | 23.0-28.0 | 21.6-27.3 |
| 11 | Lighthouse Purple | PanAmerican Seed, United States of America | Purple | 26.0-28.5 | 15.6-25.5 |
| 12 | Oasis Red | Floranova, England | Red | 24.0-26.5 | 17.8-19.1 |
| 13 | Sentry | Floranova, England | Red | 11.0-12.5 | 15.1-16.6 |
| 14 | Sailor | Floranova, England | Red | 19.0-22.0 | 15.0-16.0 |
| 15 | Sahara Red | Floranova, England | Red | 22.0-23.0 | 15.9-18.6 |
| 16 | Beauty Red | Floranova, England | Red | 16.5-19.5 | 16.0-20.1 |
| 17 | Sizzler Purple | Floranova, England | Purple | 25.0-26.5 | 18.0-22.5 |
| 18 | Sizzler Burgundy | Floranova, England | Wine red | 27.0-28.5 | 19.5-24.1 |
| 19 | Sizzler Salmon | Floranova, England | Salmon red | 14.0-26.5 | 19.2-24.3 |
| 20 | Sizzler Burgundy Halo | Floranova, England | Calyx wine red and white complex color, corolla wine red | 22.0-24.3 | 14.5-16.0 |
| 21 | Chilwee Lavender | Syngenta, Holland | Lavender | 22.5-27.0 | 17.5-23.6 |
| 22 | Chilwee Purple | Syngenta, Holland | Purple | 24.0-27.5 | 17.2-22.3 |
| 23 | Chilwee White | Syngenta, Holland | White | 22.1-23.0 | 20.7-23.2 |
| 24 | Chilwee Red | Syngenta, Holland | Red | 18.8-24.4 | 21.2-22.9 |
| 25 | Chilwee Salmon | Syngenta, Holland | Salmon red | 23.4-25.6 | 21.2-29.2 |
| 26 | Chilwee Rose bicolor | Syngenta, Holland | Flower buds rose and white in color, corolla tube rose red, edge white | 22.5-24.6 | 14.6-25.1 |
| 27 | Chilwee Scarlet bicolor | Syngenta, Holland | Flower buds red and white color, corolla tube red edge white | 19.0-22.2 | 17.2-20.8 |
| 28 | Helios | Syngenta, Holland | Red | 18.9-23.0 | 17.4-18.6 |
| 29 | Sizzler Lavender | Floranova, England | Lavender | 24.9-25.3 | - |
| 30 | Sizzler white | Floranova, England | White | 21.5-26.0 | 14.0-22.2 |
| 31 | Sizzler Red | Floranova, England | Red | 26.6-29.1 | - |
| 32 | SaSa Scarlet | Syngenta, Holland | Scarlet | 21.4-25.3 | 20.0-22.4 |
| 33 | Master | Floranova, England | Red | - | - |
| 34 | Mojave Red | Floranova, England | Red | 16.2-19.1 | - |
| 35 | Fizz Cream | Takii Seed, Japan | White | 17.6-19.4 | - |
| 36 | Fizz Grape | Takii Seed, Japan | Wine red | - | - |
| 37 | Pioneer 1 | XinYuan Seed, China | Red | - | - |
| 38 | Pioneer 2 | XinYuan Seed, China | Red | - | - |
| 39 | Notting Hill | XinYuan Seed, China | Red | - | - |
| 40 | Knight | XinYuan Seed, China | Red | - | - |
| 41 | virtuoso | Floranova, England | Red | 20.6-21.6 | 15.8-20.3 |
| 42 | ZiLinglong | Beijing University of Agriculture, China | Purple | 22.5-28.4 | 16.0-19.0 |
| 43 | HongFenjiaren | Beijing University of Agriculture, China | Rose red | 27.2-31.3 | - |
| 44 | HongQingting | Beijing University of Agriculture, China | Red | 26.4-33.5 | 12.0-14.9 |
| 45 | HongLinglong | Beijing University of Agriculture, China | Red | 26.1-32.0 | 16.2-18.4 |
| 46 | BaiMawangzi | Beijing University of Agriculture, China | White | 35.4-42.8 | 17.3-22.7 |
| 47 | Red Belt | Floranova, England | Red | 13.5-19.0 | 11.7-15.3 |
| 48 | Blue Ribbon | PanAmerican Seed, United States of America | Flowerbed blue and white color, corolla tube blue edge white | 19.8-25.5 | 18.1-23.0 |
| 49 | AoX 49 | Beijing Institute of Landscape Architecture, China | Red | 22.5-29.0 | 18.0-19.6 |
| 50 | ChiFeng Guo | ChiFeng, China | Red | 21.4-25.4 | 14.5-17.8 |
| 51 | AoX 51 | Beijing Institute of Landscape Architecture, China | Red | 18.9-20.6 | 12.8-20.5 |
| 52 | AoX 52 | Beijing Institute of Landscape Architecture, China | Red | 20.6-23.7 | 14.7-17.6 |
| 53 | AoX 53 | Beijing Institute of Landscape Architecture, China | Red | 25.5-28.0 | 13.8-18.0 |
| 54 | AoX 54 | Beijing Institute of Landscape Architecture, China | Red | 20.0-23.2 | 15.2-20.2 |
| 55 | AoX 55 | Beijing Institute of Landscape Architecture, China | Red | 25.6-30.0 | 18.0-20.0 |
| 56 | AoX 56 | Beijing Institute of Landscape Architecture, China | Red | 20.5-28.3 | 16.3-21.4 |
| 57 | AoX 57 | Beijing Institute of Landscape Architecture, China | Red | 21.8-24.5 | 14.0-17.0 |
| 58 | JingYan 1140 | Beijing Institute of Landscape Architecture, China | Red | 20.2-23.2 | 15.0-15.5 |
| 59 | AoX 59 | Beijing Institute of Landscape Architecture, China | Red | 25.1-27.3 | 12.9-15.0 |
| 60 | AoX 60 | Beijing Institute of Landscape Architecture, China | Red | 22.4-27.5 | 11.0-12.0 |
| 61 | AoX 61 | Beijing Institute of Landscape Architecture, China | Red | 21.0-22.6 | 13.2-14.6 |
| 62 | AoX 62 | Beijing Institute of Landscape Architecture, China | Red | 22.4-24.6 | 13.7-14.5 |
| 63 | AoX 63 | Beijing Institute of Landscape Architecture, China | Red | 18.8-24.6 | 17.2-20.2 |
| 64 | AoX 64 | Beijing Institute of Landscape Architecture, China | Red | 17.7-18.7 | 11.0-18.2 |
| 65 | ShiJihong | Beijing Institute of Landscape Architecture, China | Red | - | - |
| 66 | AoX 66 | Beijing Institute of Landscape Architecture, China | Red | 16.0-23.9 | - |
| 67 | Pingzhuang Zhonggao | ChiFeng, China | Red | 36.4-40.5 | 14.5-22.5 |
| 68 | Pingzhuang USA | ChiFeng, China | Red | - | - |
| 69 | AoX 69 | Beijing Institute of Landscape Architecture, China | Red | 21.3-22.2 | 14.9-15.6 |
| 70 | AoX 70 | Beijing Institute of Landscape Architecture, China | Red | 19.7-23.3 | 13.2-16.0 |
| 71 | AoX 71 | Beijing Institute of Landscape Architecture, China | Red | 16.6-19.4 | 11.6-14.2 |
| 72 | AoX 72 | Beijing Institute of Landscape Architecture, China | Red | 14.0-17.0 | 11.7-14.6 |
| 73 | AoX 73 | Beijing Institute of Landscape Architecture, China | Red | - | - |
| 74 | AoBi 1 | Beijing Institute of Landscape Architecture, China | Red | 18.0-20.5 | 11.5-16.6 |
| 75 | AoBi 3 | Beijing Institute of Landscape Architecture, China | Lavender | - | - |
| 76 | AoSheng 4 | Beijing Institute of Landscape Architecture, China | Salmon red | - | - |
| 77 | AoX 77 | Beijing Institute of Landscape Architecture, China | Red | - | - |
| 78 | AoBi 1 | Beijing Institute of Landscape Architecture, China | Fuchsia | - | - |
| 79 | AoSheng 2 | Beijing Institute of Landscape Architecture, China | Scarlet | 18.7-19.4 | 18.2-19.4 |
| 80 | AoSheng02 | Beijing Institute of Landscape Architecture, China | Red | - | - |
| 81 | AoSheng 1 | Beijing Institute of Landscape Architecture, China | Fuchsia | - | - |
| 82 | AoSheng 8 | Beijing Institute of Landscape Architecture, China | Flower white, corolla pale pink | 24.9-29.4 | 21.0-22.6 |
| 83 | AoSheng 7 | Beijing Institute of Landscape Architecture, China | Calyx wine red and white complex color, corolla wine red | 19.8-25 | - |
| 84 | AoSheng 9 | Beijing Institute of Landscape Architecture, China | Flower white, corolla purple | 28.3-22 | 10.5-16.5 |
| 85 | AoSheng 3 | Beijing Institute of Landscape Architecture, China | Lavender | 17-21.2 | 11.0-14.0 |
| 86 | AoX 86 | Beijing Institute of Landscape Architecture, China | Calyx white, corolla deep pink | 20.3-24.2 | 12.0-24.3 |
| 87 | AoSheng 6 | Beijing Institute of Landscape Architecture, China | Wine red | 16.3-20.4 | 13.6-21.0 |
| 88 | AoBi 2 | Beijing Institute of Landscape Architecture, China | Salmon red | 20.7-22.1 | - |
| 89 | AoX 89 | Beijing Institute of Landscape Architecture, China | Red | 16.0-19.2 | 11.6-16.8 |
| 90 | AoX 90 | Beijing Institute of Landscape Architecture, China | Flower white, corolla lavender | 19.6-23.2 | - |
| 91 | AoX 91 | Beijing Institute of Landscape Architecture, China | Flower white, corolla red | 17.6-25.4 | -- |
| 92 | AoSheng 5 | Beijing Institute of Landscape Architecture, China | Flower buds rose, corolla red | 22.1-23.2 | 14.7-19.1 |
| 93 | AoX93 | Beijing Institute of Landscape Architecture, China | - | - | - |
| 94 | AoBi 03 | Beijing Institute of Landscape Architecture, China | Red | 19.7-24.6 | 14.0-15.9 |
| 95 | AoBi 1 | Beijing Institute of Landscape Architecture, China | Fuchsia | 25.1-29.5 | 16.6-19.5 |
| 96 | AoSheng 03 | Beijing Institute of Landscape Architecture, China | Red | 18.7-22.8 | 12.2-15.5 |
| 97 | AoX 97 | Beijing Institute of Landscape Architecture, China | Flower blush and white multicolor, corolla red | - | - |
| 98 | AoBi 2 | Beijing Institute of Landscape Architecture, China | Salmon red | - | - |
| 99 | AoBi 3 | Beijing Institute of Landscape Architecture, China | Lavender | 25.6-29.4 | 12.0-18.3 |
| 100 | Fizz Raspberry | Takii Seed, Japan | Rose red | 20.6-23.8 | 21.4-23.9 |
| 101 | FeiFan | Hangzhou Academy of Agricultural Sciences, China | Blush and white gradient | 26.0-30.2 | 12.5-26.0 |
| 102 | HongYun | Hangzhou Academy of Agricultural Sciences, China | Red | 22.2-30.9 | 12.0-22.6 |
| 103 | ShenZhouhong | Hangzhou Academy of Agricultural Sciences, China | Red | 25.5-31.6 | 15.0-24.9 |
| 104 | AoX104 | Beijing Institute of Landscape Architecture, China | Red | - | - |
| 105 | AoX105 | Beijing Institute of Landscape Architecture, China | Red | - | - |
| 106 | AoX106 | Beijing Institute of Landscape Architecture, China | Fuchsia | - | - |
| 107 | AoX107 | Beijing Institute of Landscape Architecture, China | Gradient light wine red | - | - |
| 108 | AoX108 | Beijing Institute of Landscape Architecture, China | - | - | - |
| 109 | AoX109 | Beijing Institute of Landscape Architecture, China | - | - | - |
| 110 | AoX110 | Beijing Institute of Landscape Architecture, China | - | - | - |
| 111 | AoX111 | Beijing Institute of Landscape Architecture, China | - | - | - |
| 112 | AoX112 | Beijing Institute of Landscape Architecture, China | - | - | - |

**-** = No data.

**Table S2.** Characteristics of 312 monomorphic SSR markers for *S. splendens*.

| **Locus** | **Sequence (3' to 5')** | **Product size** | **Repetitions** | **Motif** | **Scaffold** |
| --- | --- | --- | --- | --- | --- |
| **ssps193** | F:TGATGTTGTTCGGCGTTTAG | 275 | 12 | AT | scaffold14 |
|  | R:TTGGGAGAGAAACGTTCGAG |  |  |  |  |
| **ssps194** | F:GTGGCAAAATCTGGACCACT | 299 | 11 | TA | scaffold14 |
|  | R:GTAGCGGTTTCCAGCCATTA |  |  |  |  |
| **ssps199** | F:AGGGATGAAGTAACCGATGC | 290 | 14 | AT | scaffold17 |
|  | R:CAAACCAAACCCTTCCAAAA |  |  |  |  |
| **ssps200** | F:CTGGGGTTGGTTGATAATGC | 141 | 12 | AGA | scaffold12 |
|  | R:AAACTCCGGCACCGTCTACT |  |  |  |  |
| **ssps202** | F:TCTACTCGTTGTCGGAGTCG | 215 | 12 | TCT | scaffold17 |
|  | R:CAGGTCATACGCAGGTGAAA |  |  |  |  |
| **ssps215** | F:TTCCAATGTCTCGTGGATGA | 242 | 10 | TA | scaffold19 |
|  | R:TTCGCTCTAATTGCGTTGTG |  |  |  |  |
| **ssps216** | F:GCAACGAGCAATACAACGAA | 217 | 10 | TAA | scaffold6 |
|  | R:GGTCTCTCGTTCCGTCAACT |  |  |  |  |
| **ssps217** | F:GGACGGACCAAAATGGTAAA | 254 | 17 | AG | scaffold6 |
|  | R:CTGCAACTTATTGCGTGTCC |  |  |  |  |
| **ssps218** | F:ATTTTCTCCCAATCCCCAAG | 298 | 11 | CA | scaffold6 |
|  | R:AATTTGCATGGCAACGTACA |  |  |  |  |
| **ssps219** | F:CTGCTGGAAATGAGGAGAGG | 278 | 10 | GA | scaffold6 |
|  | R:TGTGTTGGGAATCAACAGGA |  |  |  |  |
| **ssps220** | F:GCTGTGCAAATCAACACCAT | 197 | 16 | AT | scaffold6 |
|  | R:GAATGTCAACAATGCCAACG |  |  |  |  |
| **ssps221** | F:AGGCCAATGTCTCAACAAGC | 128 | 13 | GT | scaffold6 |
|  | R:TTACACACTCCATGCCATCC |  |  |  |  |
| **ssps223** | F:TTCCACGTGCCTCTCTCTCT | 168 | 13 | CA | scaffold6 |
|  | R:AGGAGGCGAGTTTGACACAG |  |  |  |  |
| **ssps225** | F:TCCCCACCAAAACGATCATA | 218 | 13 | GA | scaffold21 |
|  | R:GCCTCTGTTTCCTGGTGAGA |  |  |  |  |
| **ssps226** | F:TGTCCATGTCGTTACCTCCA | 205 | 20 | TC | scaffold21 |
|  | R:CAAATGCATCCCGATTTAGG |  |  |  |  |
| **ssps227** | F:TTGGACTTTGGACGGCTTTA | 165 | 15 | AAT | scaffold21 |
|  | R:CTTCCCCTGAATCTGGAAGG |  |  |  |  |
| **ssps228** | F:CGATGGAGATTTGGAAGGAG | 294 | 17 | TA | scaffold13 |
|  | R:TTTCTTTTGGGCTCGAAGAC |  |  |  |  |
| **ssps229** | F:AATATGAATCCCCACCACGA | 207 | 18 | TC | scaffold13 |
|  | R:ACAATTCCCTTGGCTTGAAA |  |  |  |  |
| **ssps230** | F:TTAACCTCCCAAACCACACC | 127 | 11 | TG | scaffold13 |
|  | R:CCGGCAAACTCTTCGACTAC |  |  |  |  |
| **ssps231** | F:CCCTTCACTCTCCCTCATCA | 222 | 11 | TC | scaffold13 |
|  | R:TGGTTCTTGATCTGGGTTCC |  |  |  |  |
| **ssps232** | F:TTTGTGCGTTTTGCACTTTG | 286 | 11 | AAT | scaffold13 |
|  | R:TCGACACTCATCGTTGCTTC |  |  |  |  |
| **ssps233** | F:GACTGAAAAGCTGCGAAGGA | 213 | 15 | TA | scaffold13 |
|  | R:TAGGCAACCACATCAACTCG |  |  |  |  |
| **ssps234** | F:AAAGAGTGAGGCAGCAGAGG | 252 | 19 | GA | scaffold13 |
|  | R:TGCTTTCGTCAACCCTTACA |  |  |  |  |
| **ssps235** | F:CCCCGCTTTGTTATTATGGA | 230 | 10 | TA | scaffold13 |
|  | R:TTGTCCCACAAAAGATGTCG |  |  |  |  |
| **ssps236** | F:ATATACACTCGCGCGCTCTC | 166 | 13 | TC | scaffold20 |
|  | R:CTTGATTTGGCCTCTTCTCG |  |  |  |  |
| **ssps239** | F:GGTTCAGTTCACCGAAACCT | 252 | 11 | AG | scaffold20 |
|  | R:TTGCTCCGATTTCTTTTGCT |  |  |  |  |
| **ssps241** | F:CTCCCTCCGACCACTATCAA | 135 | 15 | AG | scaffold20 |
|  | R:GAGGTGGGGCTAATTCTTCA |  |  |  |  |
| **ssps242** | F:AATGCAAAATGTGGGGAGAA | 266 | 11 | AAG | scaffold20 |
|  | R:TTTTGTTTGGCCTGACACCT |  |  |  |  |
| **ssps245** | F:AGGAAAGCTCACCTTGATCG | 271 | 17 | TTA | scaffold20 |
|  | R:GTTGTGGCCCAAATTGTTTT |  |  |  |  |
| **ssps246** | F:GCTTGCTTGCTTCCTTGTTT | 175 | 12 | TG | scaffold20 |
|  | R:CCAATTGCTGTCTCCATTCA |  |  |  |  |
| **ssps247** | F:GTGCCTATCCGCTATTGTGC | 204 | 11 | CT | scaffold20 |
|  | R:TTCGGCACGGATTTTAATGT |  |  |  |  |
| **ssps248** | F:AACAGCCTATGCACCGTCTC | 199 | 12 | AAT | scaffold20 |
|  | R:GCGCCTTTGAGGCTTATTTC |  |  |  |  |
| **ssps249** | F:ACCGTTGGATTGTGGAGTGT | 209 | 10 | CT | scaffold79 |
|  | R:ATCACACCACGGAACAGTGA |  |  |  |  |
| **ssps252** | F:TCTCCTCCTCCACGCAATAC | 260 | 11 | TC | scaffold12 |
|  | R:CATGAGCGAATCTCAACGAA |  |  |  |  |
| **ssps253** | F:TGCCGAGTTGCTTGTCTATG | 288 | 10 | TA | scaffold12 |
|  | R:CTCCGTTTGGCATTTGTTCT |  |  |  |  |
| **ssps256** | F:CAGTCACCTTCAACCCCAAT | 174 | 13 | CA | scaffold7 |
|  | R:TTGTGGGATAGCTGCAAGAA |  |  |  |  |
| **ssps257** | F:TTGGCTCCCAATTCCATAAG | 277 | 17 | TA | scaffold7 |
|  | R:TCGACAACCAAAGAAAGCAA |  |  |  |  |
| **ssps259** | F:ATCACAATACCAGCCGCAAT | 140 | 12 | GT | scaffold7 |
|  | R:TTTCTCCCCTCTTTCCTTCC |  |  |  |  |
| **ssps260** | F:TCGCGGATTCATCACATATC | 204 | 11 | CA | scaffold7 |
|  | R:AATGCCCCAAGCCTAATTTT |  |  |  |  |
| **ssps261** | F:CAAGTGCCCGAACCTCTCTA | 252 | 10 | TG | scaffold7 |
|  | R:ACTCCAGCTCCCAAATTTCC |  |  |  |  |
| **ssps262** | F:TTTCAGGACATCCCACTTGC | 166 | 15 | AC | scaffold18 |
|  | R:ACGCTCCAAATTGTGAAGGA |  |  |  |  |
| **ssps263** | F:GCTCATGGCTTTGTTTTCGT | 203 | 10 | ATG | scaffold18 |
|  | R:TTCTTCTTTCGCCAGCTTTC |  |  |  |  |
| **ssps264** | F:TTGCTTTTCCATCCCATTTC | 167 | 12 | AC | scaffold18 |
|  | R:GAATGGTTTTGCATGGATCA |  |  |  |  |
| **ssps265** | F:ACAATGACCCTCCGTGTTTT | 298 | 11 | TA | scaffold18 |
|  | R:CCCGAATCTGGACCATTAGA |  |  |  |  |
| **ssps266** | F:GATACTCGGCACGGGTTTTA | 187 | 14 | GA | scaffold18 |
|  | R:TTGCTATTTTGGGATGTCCA |  |  |  |  |
| **ssps267** | F:CTACGGGTTATGGGCAGAAA | 290 | 16 | GA | scaffold307 |
|  | R:AAAACTGTGCAACGGTCTCC |  |  |  |  |
| **ssps268** | F:TCCGGATTACTCCCTGTAGC | 166 | 11 | CT | scaffold16 |
|  | R:TTAGCACCCTAGTCCAACCA |  |  |  |  |
| **ssps269** | F:GTCCGGAATGTCGTGAAAAC | 189 | 12 | TA | scaffold49 |
|  | R:GGGCTGCATTTCTGGATTTA |  |  |  |  |
| **ssps271** | F:TCCGCGTTATCCATTTTCAT | 255 | 16 | TC | scaffold16 |
|  | R:GCATAACCGAAACCGCTAAA |  |  |  |  |
| **ssps273** | F:GGGTGTGGTTGTGTGTGTGT | 155 | 10 | GA | scaffold16 |
|  | R:CACTTTGTGAATTGCCGTGA |  |  |  |  |
| **ssps275** | F:TGAGGAGGACTCCACTGCTT | 287 | 10 | CTAT | scaffold16 |
|  | R:AGCGTGTATAGGCGACGAAC |  |  |  |  |
| **ssps276** | F:AAGGCGATTAGTGGTGAACG | 292 | 11 | TA | scaffold16 |
|  | R:TGGGTTGGATACCGGAAGTA |  |  |  |  |
| **ssps277** | F:TCCATTGCTTCTCCTTCGTT | 186 | 10 | ATT | scaffold11 |
|  | R:CACCTCAATTCCCTGGCTTA |  |  |  |  |
| **ssps278** | F:ATGCATTGTCATGCCCTTCT | 234 | 10 | AT | scaffold11 |
|  | R:TATTGGTGGCGCATCTGTAA |  |  |  |  |
| **ssps280** | F:CACTATAAATGGCGGGAGCA | 149 | 15 | TC | scaffold11 |
|  | R:GAGATTGGTTTTGGGCTGAA |  |  |  |  |
| **ssps281** | F:TGGCAGTTACTTTTCCGTCA | 254 | 14 | TA | scaffold11 |
|  | R:GGGCTGCATTTCTGGATTTA |  |  |  |  |
| **ssps282** | F:TGAATCTAGGGGACCAGCAT | 265 | 13 | GT | scaffold11 |
|  | R:CGTACATATGACCGCCCTTT |  |  |  |  |
| **ssps283** | F:CGGCGAGTACCTTAACTGGA | 237 | 18 | AG | scaffold15 |
|  | R:TGTTTGCATCAGAGGGTCAA |  |  |  |  |
| **ssps289** | F:TAAACGCAGCTGGAAAGAGG | 200 | 19 | TA | scaffold29 |
|  | R:TAGGGCCAAGATTTGGTCTG |  |  |  |  |
| **ssps295** | F:AATTGCCATGATGAGCCTTC | 211 | 11 | CT | scaffold29 |
|  | R:GCAACACCGCAATCAGTAGA |  |  |  |  |
| **ssps297** | F:ATCCCTCCCCTCTCTACTCG | 269 | 12 | CT | scaffold10 |
|  | R:CACCATCACCCCTTCAAATC |  |  |  |  |
| **ssps300** | F:GTAGCAGCAAAGGGAGTTGC | 196 | 10 | TA | scaffold10 |
|  | R:AGGCCATGAGATCACCCATA |  |  |  |  |
| **ssps306** | F:AAATTCTCCTGTGGGGCTCT | 270 | 10 | TC | scaffold10 |
|  | R:GCGATTGATTTCCTGCTGTT |  |  |  |  |
| **ssps307** | F:TGAAGCTCCAGTTGCATGTC | 248 | 11 | GA | scaffold10 |
|  | R:ATTTACACCGCTCTGGTTGG |  |  |  |  |
| **ssps310** | F:TCGGCATTGAAGGAATTAGC | 204 | 10 | AT | scaffold10 |
|  | R:CACGTTTTAGACAGCCCACA |  |  |  |  |
| **ssps311** | F:TGGGTGGAAGAGATTTGGTC | 141 | 15 | TA | scaffold33 |
|  | R:GTTCGGTGGTTCGGTCTTTA |  |  |  |  |
| **ssps312** | F:TTCGACTTTTCGGGTATTGC | 298 | 15 | AC | scaffold33 |
|  | R:TTGGTGCATGGTTGTTGAGT |  |  |  |  |
| **ssps313** | F:AATGATGTTGTTCGGCGTTT | 244 | 18 | TA | scaffold33 |
|  | R:TGCAAAATAGACCGTTCGTG |  |  |  |  |
| **ssps316** | F:GCTCATGTCCATCCAGTTCC | 221 | 12 | GA | scaffold28 |
|  | R:GAGTCCCGAGTCGTTGTGAT |  |  |  |  |
| **ssps317** | F:GTCGAACATGGGCTTGTTTT | 238 | 12 | CAT | scaffold28 |
|  | R:GACAATATCGCCGACCTCAT |  |  |  |  |
| **ssps319** | F:GCTTCCTCTCGGCTCTCTCT | 158 | 10 | CT | scaffold28 |
|  | R:CCACCTTCTACCTTCCACCA |  |  |  |  |
| **ssps320** | F:TGGCCCAGATTTGGTCTCTA | 139 | 12 | AT | scaffold8 |
|  | R:TCTGGACCGTTGATGAATGA |  |  |  |  |
| **ssps323** | F:CCGCCATTAGGAGTCACATT | 190 | 17 | TA | scaffold25 |
|  | R:GGCTGAGATTTGGTCTGCAT |  |  |  |  |
| **ssps326** | F:TAAAAGGTAGCCGGGTTGAG | 158 | 13 | AG | scaffold32 |
|  | R:CCGGGTCAGGTTACAACTTT |  |  |  |  |
| **ssps327** | F:CCCAATTTCTGGGTCAATGT | 282 | 17 | ATT | scaffold32 |
|  | R:GGGACAATATTTGGGTGACG |  |  |  |  |
| **ssps332** | F:GCGAACGTGAGACACGACTA | 171 | 11 | AC | scaffold34 |
|  | R:GCTCTGACCCAAACCCATTA |  |  |  |  |
| **ssps333** | F:ACCATTTTCCCTTTCCTGCT | 140 | 11 | AG | scaffold34 |
|  | R:GCAACACTCTGCAGCCATAA |  |  |  |  |
| **ssps335** | F:CTCTCGCTCTCAATCCCAAG | 190 | 12 | GAT | scaffold30 |
|  | R:TTCCTCCTCATCCTCCTCCT |  |  |  |  |
| **ssps340** | F:GCCTCAGGGGACTCTTGTTT | 135 | 11 | GA | scaffold30 |
|  | R:TACTCGGGCTGGACCTACTG |  |  |  |  |
| **ssps341** | F:CGGCTTCTTCATTCCATTTC | 245 | 15 | AGA | scaffold30 |
|  | R:TCAGAAATGCCCCAAAATTC |  |  |  |  |
| **ssps342** | F:TGATCCGTTGATGCCTTAGA | 213 | 12 | TA | scaffold27 |
|  | R:TCCCCACATACTACGACTGC |  |  |  |  |
| **ssps345** | F:CAAGTCCAAAGACGACAGCA | 211 | 12 | AC | scaffold27 |
|  | R:TGAAATGGAGATCCCAGAGG |  |  |  |  |
| **ssps346** | F:CGCATGTTTATGGCGTTATG | 294 | 15 | TA | scaffold27 |
|  | R:CCATTCTGCCCTTTCACAAT |  |  |  |  |
| **ssps349** | F:AAGAATGAAAGCGACGGCTA | 197 | 16 | AT | scaffold35 |
|  | R:TCCAGCGAATTGGATTCTTC |  |  |  |  |
| **ssps350** | F:TTACTTCCTCCGTCATGCAA | 285 | 19 | TA | scaffold35 |
|  | R:TCCCTCCATCCCACAATAAG |  |  |  |  |
| **ssps351** | F:ATCTTCCGCCCTCTTCTCAT | 177 | 10 | CA | scaffold5 |
|  | R:AATTCGCCACAGTTTTCTGC |  |  |  |  |
| **ssps352** | F:CAATGGAGCCTGAAGGAAGA | 143 | 15 | TA | scaffold5 |
|  | R:ACGATCCGGTTTCAAAACAA |  |  |  |  |
| **ssps353** | F:TTTCTCAGCATCCCAATTCC | 256 | 13 | TC | scaffold5 |
|  | R:GACATGAGGCGGTACCTAGC |  |  |  |  |
| **ssps354** | F:ATGAGTGTATTCGGGGCGTA | 194 | 12 | AT | scaffold5 |
|  | R:GGAGGAAGAGAGGGGAATTG |  |  |  |  |
| **ssps355** | F:ATGGTGGGGTGATCATTTTG | 251 | 10 | GT | scaffold5 |
|  | R:TTCACCCACAAACACTTCCA |  |  |  |  |
| **ssps356** | F:AGACCCACGGAGACGTTATG | 132 | 10 | GA | scaffold5 |
|  | R:CCATGCTTTCCATTTCACCT |  |  |  |  |
| **ssps360** | F:CTGGATCAACCCCAGATGAC | 263 | 13 | TC | scaffold23 |
|  | R:CTGAGCAGATTGCCAGACAG |  |  |  |  |
| **ssps362** | F:GCTTGTGCAGTCTCTTGTGC | 189 | 10 | TGT | scaffold23 |
|  | R:ATGGAAGTGGTCGACATTGG |  |  |  |  |
| **ssps363** | F:GCCACTTGTCAACTCCGTCT | 229 | 10 | TG | scaffold23 |
|  | R:GAATCGTAGCAGGCCACAAC |  |  |  |  |
| **ssps365** | F:GACGAGAACAACACGAGCAA | 236 | 13 | AG | scaffold23 |
|  | R:ATCCGTAAATCGCATTCAGC |  |  |  |  |
| **ssps366** | F:AGAACAAGGCCAACCAGAGA | 161 | 10 | GT | scaffold23 |
|  | R:AAAGAACTGCACCACCAACC |  |  |  |  |
| **ssps367** | F:TTCTCCTGCCTCCACATAGG | 233 | 13 | TTA | scaffold23 |
|  | R:GGAGAAATGCTTGGAGATGG |  |  |  |  |
| **ssps369** | F:CTCCGTTTGGCATTTGTTCT | 171 | 11 | TA | scaffold23 |
|  | R:TAGGGATGGTGAGGTTTGGA |  |  |  |  |
| **ssps371** | F:CGTAAGTGTGCCCCAGTTTT | 239 | 20 | TTA | scaffold1 |
|  | R:TGAATGCAACGGCTAATCAA |  |  |  |  |
| **ssps372** | F:ACTAGGCCCGAGGGAAATTA | 236 | 15 | AT | scaffold1 |
|  | R:CAATTATGCTTTCCCCGTTT |  |  |  |  |
| **ssps375** | F:GGGATTGCTATTGCTAGATGG | 205 | 10 | TA | scaffold1 |
|  | R:TGACAACAAATGAGAGGAGGA |  |  |  |  |
| **ssps376** | F:AGAGAGCGAAGGGAGGAAAG | 284 | 14 | GT | scaffold1 |
|  | R:ACGTTGAGACGCTGGATTTT |  |  |  |  |
| **ssps379** | F:GAACAACATTGCTGCCTTCA | 250 | 11 | AC | scaffold1 |
|  | R:CCCCGTCTAATCACGAGGTA |  |  |  |  |
| **ssps382** | F:TTTGGGCTATGGTCGGTTAG | 162 | 10 | GAG | scaffold116 |
|  | R:TGCAGAAAAGCCTCCATCTT |  |  |  |  |
| **ssps384** | F:CTCCATGGGAGGGTGTAGAA | 143 | 15 | AC | scaffold2 |
|  | R:GAGCCTTTCGTCCCATGATA |  |  |  |  |
| **ssps386** | F:TCGGCGTCTCTCTCTCTCTC | 259 | 15 | AT | scaffold24 |
|  | R:TAGGGCCAAGATTTGGTCTG |  |  |  |  |
| **ssps392** | F:CCCATCATTTTGCCCTATGT | 297 | 15 | AT | scaffold40 |
|  | R:AGGTGGATGAGAGGGTTGTG |  |  |  |  |
| **ssps397** | F:TTGCCCACTGAGTAGCATGT | 244 | 19 | TA | scaffold39 |
|  | R:CCAAAGTATGGCGGTTCAAT |  |  |  |  |
| **ssps398** | F:TTTGGGCTTTCTATGCTGCT | 175 | 13 | TC | scaffold39 |
|  | R:CATGGCAGCTTGTGCAATAC |  |  |  |  |
| **ssps400** | F:CGGAAATTGGGGTTATCATT | 198 | 11 | TA | scaffold39 |
|  | R:ACCAAATCTGCCCTTACTCG |  |  |  |  |
| **ssps402** | F:CGAAGCTGCCACATAAATGA | 267 | 10 | AG | scaffold247 |
|  | R:CTGCGACAGATGGGAATTTT |  |  |  |  |
| **ssps403** | F:ACGACCTCCGTTTGACATTT | 271 | 18 | TA | scaffold9 |
|  | R:GCCGTTTACCCAGTAGCAAG |  |  |  |  |
| **ssps406** | F:GCCCAAGAGGAAGAAGGCTA | 138 | 16 | AG | scaffold9 |
|  | R:TTATTCGCAAGCCACCAAAT |  |  |  |  |
| **ssps408** | F:GATGGCCCGATTAGGATAAA | 221 | 13 | TA | scaffold9 |
|  | R:TGATCCGTTGATGCCTTAGA |  |  |  |  |
| **ssps409** | F:CAACCTGCTCCACCTGAAAT | 207 | 11 | AT | scaffold9 |
|  | R:TTCGAAAGGGTGCGATAAAC |  |  |  |  |
| **ssps412** | F:GTGGCTGCGATTTGTTCTCT | 209 | 10 | TA | scaffold9 |
|  | R:CGCTGCTCCAATTGTATGTG |  |  |  |  |
| **ssps413** | F:CGATGGCAAAGGGTTTCTAA | 263 | 10 | GA | scaffold9 |
|  | R:AGAATGAGCGACGACGTTTT |  |  |  |  |
| **ssps414** | F:GGCTTGCTTTACGTTTTGGA | 183 | 14 | AC | scaffold26 |
|  | R:GCAGTCAACGAATCAAACGA |  |  |  |  |
| **ssps415** | F:CATGGACCAGACACTCTTGC | 165 | 11 | AG | scaffold26 |
|  | R:CAGAGCAAAGTTGGCATCAA |  |  |  |  |
| **ssps416** | F:CCAATTTTCCGCCAAACTAA | 183 | 10 | GA | scaffold26 |
|  | R:AGCATTTCCGATGAAGATGG |  |  |  |  |
| **ssps417** | F:TTTGTGCACTCACCTTCGTC | 229 | 10 | AT | scaffold26 |
|  | R:GGCGCTTAGCTCATAACTCG |  |  |  |  |
| **ssps418** | F:GAGACCTTAACCCGCTGTGA | 250 | 13 | ATT | scaffold26 |
|  | R:TTTGTGCGTTTTGCACTTTG |  |  |  |  |
| **ssps419** | F:TCATTCGTGGGCTGTACTCA | 278 | 17 | ATT | scaffold26 |
|  | R:ATATGAAGCCGGGAAGAAGG |  |  |  |  |
| **ssps420** | F:ACGTGCAGAGCCCAATTTAT | 248 | 16 | ATA | scaffold44 |
|  | R:GCTTAGCGCTCCTACAGGTT |  |  |  |  |
| **ssps421** | F:ACGAAATCGCAACTGCTTCT | 241 | 13 | CA | scaffold44 |
|  | R:TCCAGCCTTGACTTCGACTT |  |  |  |  |
| **ssps422** | F:GAGCCACCAGTTTTCACCTC | 271 | 13 | TAA | scaffold44 |
|  | R:ATGCAGGCGTGGTTTATAGG |  |  |  |  |
| **ssps423** | F:GAATACTCGTGGCCGTTGTT | 265 | 10 | TA | scaffold44 |
|  | R:TCACACTGCAGAATCCTTGG |  |  |  |  |
| **ssps424** | F:GGAGAAGAGAGGGCCGTAGT | 210 | 10 | TC | scaffold49 |
|  | R:CGTCGATTTCCAGCTTCTTC |  |  |  |  |
| **ssps425** | F:TCTACGACTTGTCGCACTCG | 163 | 14 | AT | scaffold45 |
|  | R:CACAGCAGCACATCTCATCA |  |  |  |  |
| **ssps431** | F:CCGAGCTAGCCATAGGTTGA | 168 | 12 | AT | scaffold41 |
|  | R:GTAAGCACGGGAAGCACAAG |  |  |  |  |
| **ssps432** | F:GGGTGCACTAACGAAAGGAA | 150 | 10 | GT | scaffold41 |
|  | R:GAGTGGGGAGAAGGGATAGG |  |  |  |  |
| **ssps436** | F:TGAGCTTTTGGGACAACACA | 244 | 10 | AT | scaffold52 |
|  | R:GTTGTGGATGAGTGGCTGAA |  |  |  |  |
| **ssps437** | F:GCTGTCTCCTCCTTGAGTGG | 278 | 11 | GT | scaffold52 |
|  | R:ATCCATGCTTGATTGCCTTC |  |  |  |  |
| **ssps438** | F:AGCTTGGCATGACAGTAGGG | 288 | 15 | AC | scaffold52 |
|  | R:ACGGCTCCTCCGATGTTATT |  |  |  |  |
| **ssps439** | F:GAGAAGAAATGGCGGATCAA | 163 | 12 | TC | scaffold52 |
|  | R:TCCAGCGTAGAAACAAAGCA |  |  |  |  |
| **ssps440** | F:TGAATGGCGTAAATCATGGA | 244 | 15 | TA | scaffold52 |
|  | R:TTTGATCAGCTCCCTCCCTA |  |  |  |  |
| **ssps443** | F:TGACAAAATCGTAGGGCTGA | 268 | 17 | AT | scaffold58 |
|  | R:GGGGAAAAGGAGAATGAGGT |  |  |  |  |
| **ssps444** | F:CCGTCCCCAAAGAGTATGAA | 249 | 11 | AG | scaffold3 |
|  | R:TTTCATCCATCCCCAAAGAG |  |  |  |  |
| **ssps450** | F:GGGAAATGGAGATGGTGATG | 259 | 20 | ATA | scaffold3 |
|  | R:AACTGGGTTCGTTGGGTTTT |  |  |  |  |
| **ssps454** | F:GCCTCATTCCCCACTAACAA | 178 | 12 | TC | scaffold3 |
|  | R:TGGGACGGAGGGAGTACTTA |  |  |  |  |
| **ssps455** | F:CTTTGAACCTGAACCGAACC | 289 | 12 | AT | scaffold59 |
|  | R:TTCCAATGGGGACTCCTAAA |  |  |  |  |
| **ssps456** | F:CATTGCATCAACCCATCAAC | 265 | 11 | TTA | scaffold59 |
|  | R:GCAAAAGCGTAGGTCCACAT |  |  |  |  |
| **ssps457** | F:ATGGGCCCTAAAGTTGCATT | 279 | 11 | GT | scaffold56 |
|  | R:CCACACCGTTTTGCTTACCT |  |  |  |  |
| **ssps458** | F:AGTGGGGAAAGTGGAGGAAT | 262 | 12 | TC | scaffold56 |
|  | R:AACCAACTTTGCTGGGTTCA |  |  |  |  |
| **ssps461** | F:AACCATCCATTCCTCTGCAA | 161 | 12 | AC | scaffold56 |
|  | R:AGTTTCTCCTCCGTCAGCAG |  |  |  |  |
| **ssps463** | F:CGAATCCGCTCTGATACCAT | 188 | 18 | AT | scaffold56 |
|  | R:TTTGGTTAAGGGGTGTTTGC |  |  |  |  |
| **ssps464** | F:CAGTTGGCCGTATACCTTCC | 180 | 10 | AC | scaffold57 |
|  | R:ACCTGCCAAATTCGTTCATC |  |  |  |  |
| **ssps465** | F:GCCAAGATTTGGTCTGCATT | 268 | 11 | TA | scaffold48 |
|  | R:GATGCGAGAAGCAGGACTCT |  |  |  |  |
| **ssps468** | F:ATCGCAGATTTGGAAGGAGA | 268 | 11 | TA | scaffold191 |
|  | R:TAGGGCCAAGATTTGGTCTG |  |  |  |  |
| **ssps470** | F:ATCGCCTCGTTTGTAACACC | 220 | 12 | AT | scaffold48 |
|  | R:TTCAAAATCCAAACCGAACC |  |  |  |  |
| **ssps472** | F:TTGAGGCATGTCCAATTGTC | 202 | 11 | TG | scaffold55 |
|  | R:CCAGCACTGGCAATGAAATA |  |  |  |  |
| **ssps473** | F:TGTGTTCAATCTTGGCATCC | 261 | 10 | CT | scaffold55 |
|  | R:ATACTTCCTCCCCTCGGCTA |  |  |  |  |
| **ssps474** | F:ACGCCAGTTCAAACTCAACC | 216 | 12 | AT | scaffold55 |
|  | R:CGTTTGGCATTTGTTCTACG |  |  |  |  |
| **ssps475** | F:TCTTCCCGGTTGATTTCTTC | 134 | 13 | CT | scaffold55 |
|  | R:CTCTCATCCCAATTCCAAGC |  |  |  |  |
| **ssps477** | F:AATCAAACCCCTCGTCCACT | 261 | 10 | AC | scaffold46 |
|  | R:AAACTTTTCTGGCCCTGGTT |  |  |  |  |
| **ssps478** | F:ACGCACATAAACACCCACAC | 160 | 10 | CT | scaffold46 |
|  | R:TATTGCTCACCGATCACTGC |  |  |  |  |
| **ssps479** | F:AACCAAGATGGATGGATGGA | 245 | 18 | CT | scaffold46 |
|  | R:GTGCAGCAGGAACCAAGAAG |  |  |  |  |
| **ssps480** | F:AGTGACAGTAAGGGGCAAGG | 161 | 11 | TA | scaffold46 |
|  | R:GGGATTTTGTTTCAGCGGTA |  |  |  |  |
| **ssps481** | F:AGCTGAAATCCCCTTGGAGT | 184 | 14 | TC | scaffold46 |
|  | R:TGGTAGTGCTGCTGGATGAG |  |  |  |  |
| **ssps482** | F:GATCGTTTTAGGTCATGCTTTG | 207 | 12 | TA | scaffold46 |
|  | R:GCATGATATTGTTCTGCGTTTC |  |  |  |  |
| **ssps483** | F:GAATAATTGGGCCACCTTGA | 276 | 11 | CT | scaffold64 |
|  | R:GTGTTTTGGTGGCAGAGGTT |  |  |  |  |
| **ssps484** | F:CTCTGATGCAACGACTCCAC | 184 | 14 | GTT | scaffold67 |
|  | R:TCCATCACCTCCGGATACAT |  |  |  |  |
| **ssps485** | F:CCCTTCTGGTCTGAGATTGC | 283 | 10 | AG | scaffold67 |
|  | R:TTCACAAATGACCGTTGCTC |  |  |  |  |
| **ssps486** | F:GTGATGCCCAAATCTCATCA | 136 | 13 | AC | scaffold67 |
|  | R:CTCCACCGGATTTCAGAGAG |  |  |  |  |
| **ssps487** | F:GAATCGTCCCCAAGTACCAA | 182 | 18 | AT | scaffold67 |
|  | R:TTGATTTCACGCTGCAAGAG |  |  |  |  |
| **ssps489** | F:TTACTGGCTGAAGCCCAAAT | 286 | 10 | GA | scaffold54 |
|  | R:TGGATCAACGCAAATCGTAA |  |  |  |  |
| **ssps490** | F:GAATGCTTCGTCCATCTCCT | 227 | 10 | CT | scaffold72 |
|  | R:GTATGTTGGCATCAGCATCG |  |  |  |  |
| **ssps491** | F:GCTCCACGGTCCTTTAGTTG | 201 | 13 | GA | scaffold73 |
|  | R:GCAGCCTGTTGGTTTAGGAA |  |  |  |  |
| **ssps494** | F:TGTCCTCACAAAGCAATCCA | 127 | 11 | GA | scaffold74 |
|  | R:CGGACTCAGCAATACGAACA |  |  |  |  |
| **ssps497** | F:CCAAAGCCAAACTGAAATGG | 172 | 11 | GA | scaffold62 |
|  | R:TTGTGGTTTCTGCTCTGGTG |  |  |  |  |
| **ssps498** | F:ACAGTCATAGCCGGGTTCAG | 177 | 10 | ACC | scaffold60 |
|  | R:GCAGGCACCCTACAGGTAAA |  |  |  |  |
| **ssps499** | F:TTTGGTATGGTTCGGTGGTT | 123 | 10 | TA | scaffold60 |
|  | R:GGGCTGCATTTCTGGATTTA |  |  |  |  |
| **ssps503** | F:CTCAAGGTTAGACGGGCAAG | 288 | 20 | AT | scaffold68 |
|  | R:CAACCCCAGAGTGGAAAAGA |  |  |  |  |
| **ssps504** | F:TGGCACTTGAGAGTGAGCAT | 285 | 14 | CT | scaffold68 |
|  | R:GCTTCTTTTCCACGTTGCTT |  |  |  |  |
| **ssps506** | F:TTGAACCCCAGAGGAAGTTG | 197 | 12 | AT | scaffold61 |
|  | R:ACGCCATCACACGCTTTATT |  |  |  |  |
| **ssps509** | F:CGCAAAAGCGAGCATACATA | 184 | 18 | AC | scaffold61 |
|  | R:CCTGTGAACATGTGGGTCAG |  |  |  |  |
| **ssps513** | F:TCCTGAAAATGCAGCAAAGA | 251 | 17 | AT | scaffold81 |
|  | R:AGATGGGCATTGTGATTGGT |  |  |  |  |
| **ssps515** | F:TGCCAACCCAATATTCTTCA | 185 | 10 | TG | scaffold63 |
|  | R:CGACCATATCGCTACGAACA |  |  |  |  |
| **ssps516** | F:ATGGTCAGGATTTGGTCTGG | 162 | 17 | TA | scaffold4 |
|  | R:CTCCGTTTGGCATTTGTTCT |  |  |  |  |
| **ssps518** | F:CTCCGTTTGGCATTTGTTCT | 153 | 15 | TA | scaffold80 |
|  | R:AAATCGTGGGGCTGAGATTA |  |  |  |  |
| **ssps519** | F:TCGCTCCATTCTCCTTGAAA | 232 | 12 | ATA | scaffold80 |
|  | R:TCTGGAATGAGCACGATCAG |  |  |  |  |
| **ssps521** | F:ATGCCAGGTCAATTCACACA | 229 | 11 | GA | scaffold65 |
|  | R:GTGATGGAAGGCCAGTTGAG |  |  |  |  |
| **ssps522** | F:TTGCGGGATTATCTTCTTGC | 184 | 20 | GAT | scaffold65 |
|  | R:TCTTTCCAACCCCTGACATC |  |  |  |  |
| **ssps524** | F:TCGCCAGACATTTTGTGATG | 260 | 11 | TAA | scaffold85 |
|  | R:ATCATCACGCAGACGCAATA |  |  |  |  |
| **ssps525** | F:TTGAGAAGCTCACGTGGTTG | 176 | 10 | AG | scaffold89 |
|  | R:ACCAGTTCACCTCCGATGAC |  |  |  |  |
| **ssps526** | F:AGCGACAGTGTGTGTGTGTG | 277 | 17 | GA | scaffold89 |
|  | R:TCAAACAATACAGGCGGATG |  |  |  |  |
| **ssps527** | F:CGGTCCCAAGAAGAAGTCTG | 213 | 11 | CT | scaffold79 |
|  | R:TACAATCCACGGAAATGCAA |  |  |  |  |
| **ssps528** | F:TTAGCCCAGGACGAAAAGAA | 254 | 12 | TA | scaffold76 |
|  | R:CCATGTCAACAATCCGACAC |  |  |  |  |
| **ssps529** | F:CAACAATCAGCCACGTGAAC | 243 | 13 | AG | scaffold78 |
|  | R:GCAAGGAATCGGAATCTCAA |  |  |  |  |
| **ssps532** | F:GGCCATTTGAGATGCCTTTA | 205 | 12 | CT | scaffold77 |
|  | R:AGAGAGAGGGCTGCCTCATA |  |  |  |  |
| **ssps533** | F:TCGGTTCCACAAATTCCTTC | 266 | 10 | CA | scaffold70 |
|  | R:TTCACACGGAAATGGACAAC |  |  |  |  |
| **ssps534** | F:ATGGAATTTTGGAGGGGTTT | 242 | 10 | AC | scaffold8 |
|  | R:AGCATGTGCATTGTCTGCAT |  |  |  |  |
| **ssps536** | F:GTGTGTGTGACATCCCCAAC | 294 | 11 | TA | scaffold8 |
|  | R:CACACGTGCACTGCTAACAA |  |  |  |  |
| **ssps540** | F:CCTTATGACGTGGCAGTGAA | 220 | 10 | CT | scaffold8 |
|  | R:CGAGAAGGGAATGTGAGAGC |  |  |  |  |
| **ssps541** | F:CTTGTCACCTGAGCCTCCAT | 200 | 12 | GA | scaffold8 |
|  | R:TAAGTCCAGAGAGCGGAAGC |  |  |  |  |
| **ssps542** | F:TTTTCCTGCATTCATCACCA | 230 | 10 | AC | scaffold8 |
|  | R:CCTTGGCCGTGATTACATTT |  |  |  |  |
| **ssps545** | F:TCAATCATGGGAAGGTTGGT | 156 | 11 | AT | scaffold8 |
|  | R:GAATGGTTTTGCATGGATCA |  |  |  |  |
| **ssps550** | F:GCAAACGCCTGCTCTTTTAT | 231 | 10 | TA | scaffold88 |
|  | R:CCATTCTGCCCTTTCACAAT |  |  |  |  |
| **ssps551** | F:GGGCCGTTAGATCTCATTCA | 155 | 18 | TA | scaffold88 |
|  | R:TGGATCGGCTCAAATAAAGG |  |  |  |  |
| **ssps552** | F:GTGGCAAAATCTGGACCACT | 212 | 11 | TA | scaffold88 |
|  | R:GTGGCAAAATCTGGACCACT |  |  |  |  |
| **ssps556** | F:GGCTGCCCAAAACCTATACA | 237 | 11 | TC | scaffold92 |
|  | R:GATTTGGCGAGACAAGCATT |  |  |  |  |
| **ssps557** | F:GATTGGGAATTGCCTAAAGGA | 218 | 10 | TG | scaffold92 |
|  | R:CCGGACACACAAACACACTAA |  |  |  |  |
| **ssps562** | F:CGACTGGAGAAACGAGGAAA | 235 | 11 | GA | scaffold98 |
|  | R:CCAACTGGTCGCATGTATTG |  |  |  |  |
| **ssps564** | F:CGGAATTCAGCAAATCCACT | 205 | 18 | GA | scaffold93 |
|  | R:CTTTTCCGCCTTTCCTTTTC |  |  |  |  |
| **ssps565** | F:CGGAGGGTCATTGTAAAGCA | 295 | 12 | TA | scaffold93 |
|  | R:TCCACTCTCTTCCTGGCTTG |  |  |  |  |
| **ssps566** | F:GAGTAGGGGAGGACGAAACC | 229 | 12 | TA | scaffold93 |
|  | R:CACTCGTTCTGCGTGTGACT |  |  |  |  |
| **ssps567** | F:TGTAGGGGCTGAGCTTCATT | 215 | 12 | AT | scaffold107 |
|  | R:CGTAGGATTCAGTTGCCACA |  |  |  |  |
| **ssps568** | F:TGCTTGATGAGTCTGGGTTG | 149 | 10 | TG | scaffold107 |
|  | R:TCCCGTAATGAACACCTTCC |  |  |  |  |
| **ssps569** | F:TCGCCACCAATCAATCACTA | 294 | 16 | AT | scaffold107 |
|  | R:TCGCTCAAAGCCTCTAGCAT |  |  |  |  |
| **ssps570** | F:CGTTGGTGGTCCAAGTAAGC | 245 | 10 | AT | scaffold102 |
|  | R:TTTCAGAATTTTGGCGAACC |  |  |  |  |
| **ssps571** | F:TTTCGGTTCTTGTGGGTTTT | 260 | 11 | CA | scaffold102 |
|  | R:CCCGATTTACCCCAACTTCT |  |  |  |  |
| **ssps573** | F:AGAGGACTGGTCCGAGGAAT | 282 | 15 | CT | scaffold101 |
|  | R:ATGACTACCGGCATCGTTTT |  |  |  |  |
| **ssps575** | F:CCGGACATTTTGTGATGAGA | 227 | 12 | AT | scaffold86 |
|  | R:TGATCCGTTGATGCCTTAGA |  |  |  |  |
| **ssps576** | F:AAGCTCTCAGGAGCACCAAT | 250 | 13 | TA | scaffold86 |
|  | R:GGGCCAAGATTTGGTCTGTA |  |  |  |  |
| **ssps578** | F:TGGGGAATTGAAGAGAGGTG | 154 | 19 | AAT | scaffold83 |
|  | R:TTTCTGCTCCCAAAATTGCT |  |  |  |  |
| **ssps580** | F:ACAAGTGTCAGCCGAATTGA | 273 | 11 | CA | scaffold96 |
|  | R:CGAATTTGGGGAAACGATTA |  |  |  |  |
| **ssps583** | F:TCCACAAACACACGTGAACA | 255 | 19 | AT | scaffold103 |
|  | R:TGGTGCCATTTGACGAACTA |  |  |  |  |
| **ssps584** | F:CTTGAAAACCGAACACCACA | 215 | 15 | AT | scaffold103 |
|  | R:TGCTAGAAATGGGCCAAGAC |  |  |  |  |
| **ssps585** | F:TGCCAAAGGAGCTGGTACAT | 193 | 12 | TA | scaffold103 |
|  | R:CAGTTGTCGACTCCGTCTCC |  |  |  |  |
| **ssps591** | F:TTATGGAGAATGGGGTGGTT | 264 | 12 | AC | scaffold84 |
|  | R:CCTTCTTCCTCTCACGTTGC |  |  |  |  |
| **ssps599** | F:TTGATGGTCATTGGATGCTC | 179 | 11 | CA | scaffold97 |
|  | R:CAGCCTCTTGTGTGTGTGTG |  |  |  |  |
| **ssps601** | F:TTGATGGTCATTGGATGCTC | 297 | 12 | CA | scaffold97 |
|  | R:AGCCCATTTGATGCAACTTT |  |  |  |  |
| **ssps602** | F:CGTGGTGGACTGATGCTAGA | 242 | 10 | AAAG | scaffold117 |
|  | R:TGCAGAGCGATGCTGATTAC |  |  |  |  |
| **ssps604** | F:CCATTGAATCCCAATCATCC | 189 | 11 | CTT | scaffold117 |
|  | R:ATGTGATGATGAGCCACTGC |  |  |  |  |
| **ssps606** | F:TCGAAGTAAGGGCGGAAATA | 222 | 12 | AG | scaffold113 |
|  | R:TCATCAATGGCGCTGTAGTC |  |  |  |  |
| **ssps611** | F:TCTCCAACCCTCACATTGCT | 292 | 11 | GAA | scaffold122 |
|  | R:GCTTACCGCTTTCCACAGAC |  |  |  |  |
| **ssps614** | F:AGTTTGAGTGCCGAATCGTT | 263 | 12 | TA | scaffold127 |
|  | R:CAGTCAACCTCTTCCCAAGC |  |  |  |  |
| **ssps615** | F:CAGTCAACCTCTTCCCAAGC | 281 | 20 | AT | scaffold127 |
|  | R:AGTTTGAGTGCCGAATCGTT |  |  |  |  |
| **ssps617** | F:ACCCTGGTTAGTTGCCCTCT | 225 | 15 | AT | scaffold114 |
|  | R:TGGACAAAGGAAGTGCATGA |  |  |  |  |
| **ssps618** | F:CCCCGGTTTGAAGTAAATTG | 246 | 18 | TA | scaffold129 |
|  | R:AAATCGTGGGGCTGAGATTA |  |  |  |  |
| **ssps622** | F:AACTTTATCCCGCCCTCTGT | 151 | 10 | TC | scaffold134 |
|  | R:CTTCCAGATCCGTCAATCGT |  |  |  |  |
| **ssps627** | F:ATGTCATCTCACGCCAAACA | 260 | 10 | TG | scaffold143 |
|  | R:GCGACGAACATGCTTTTACA |  |  |  |  |
| **ssps630** | F:CCTCCATAGAGCACCTCCAC | 161 | 13 | TC | scaffold150 |
|  | R:GCCACGATTTCACATCCTTT |  |  |  |  |
| **ssps631** | F:GGGGAAGGTAGCAGAGGTTT | 212 | 10 | GA | scaffold147 |
|  | R:CATCAATCACCACCAACTGC |  |  |  |  |
| **ssps632** | F:ACTATGCAGGGCACGTAAGG | 174 | 10 | AC | scaffold131 |
|  | R:GAGGGAGAAGGCTGGGTATT |  |  |  |  |
| **ssps633** | F:ATGTTGGCCTACTGCACACA | 271 | 10 | AT | scaffold131 |
|  | R:ATCGCTTGAATGACGAATCC |  |  |  |  |
| **ssps635** | F:ACAAACCAGCCTCCCTCTTC | 201 | 14 | TA | scaffold144 |
|  | R:GGCTCAGTTGCTGGAGTTGT |  |  |  |  |
| **ssps639** | F:TCGACAGAGAGAGTGGTTGG | 238 | 17 | TG | scaffold157 |
|  | R:ACCAATGCCGACAGATTTTC |  |  |  |  |
| **ssps641** | F:TGTCTGTCTCTGTGCGTGTG | 230 | 13 | GA | scaffold158 |
|  | R:GGGATTCAAAACCTTGCAGA |  |  |  |  |
| **ssps642** | F:TCTTCCTCCTCCTCCTCCTC | 162 | 10 | TCA | scaffold158 |
|  | R:CTCTCGCTCTCAATCCCAAG |  |  |  |  |
| **ssps643** | F:CGGCTGGAATGACAAAGAAT | 232 | 10 | GA | scaffold118 |
|  | R:AACACCGTCCAAAACAGTCC |  |  |  |  |
| **ssps645** | F:AAGGAAGCGGGAGAAGAGAG | 234 | 10 | TA | scaffold166 |
|  | R:TTCCCCAATTTTCCACACAC |  |  |  |  |
| **ssps646** | F:AGCTAGCCGTGAAAGCAAAA | 235 | 10 | CT | scaffold155 |
|  | R:ATGCGATGTGTGTGTGTGTG |  |  |  |  |
| **ssps655** | F:TGCACGAGCAGTTCTTCCTA | 129 | 10 | ATT | scaffold179 |
|  | R:ACGGTCATCTTTGGCTCTTG |  |  |  |  |
| **ssps656** | F:GTGTGTGTGTGTGTGCGTGT | 136 | 10 | GA | scaffold186 |
|  | R:CTTCCTTGGCTGGTTGTGAT |  |  |  |  |
| **ssps657** | F:GCCACAGTGAGGGGTAAAGA | 288 | 19 | AT | scaffold184 |
|  | R:CGGATCGCTACTCTCGAAAC |  |  |  |  |
| **ssps659** | F:TTCCTCCCGTCCCATAATAA | 158 | 11 | AT | scaffold133 |
|  | R:GGGGCTGAAATCAAGAATGA |  |  |  |  |
| **ssps660** | F:CTACCACCAGGTCTCGAAGG | 237 | 10 | AG | scaffold181 |
|  | R:TTTGCCTCCACTTTCTCGTT |  |  |  |  |
| **ssps661** | F:GAGATTCCCCGCAGGATTAG | 289 | 17 | GA | scaffold188 |
|  | R:CCTTCCAATTTCACGCGTAT |  |  |  |  |
| **ssps662** | F:GGAATTGACTGCATCTACGG | 148 | 10 | TG | scaffold173 |
|  | R:TGAACTGCCTACCCTCCTCT |  |  |  |  |
| **ssps664** | F:TTTCCAATATCCCGTGCATT | 225 | 10 | ATA | scaffold190 |
|  | R:TTAGAGCTGGCCAAACAACA |  |  |  |  |
| **ssps665** | F:TCCGTTACATCTCCCACTCC | 225 | 12 | CT | scaffold190 |
|  | R:GCCCATTTCTGATCGATTGT |  |  |  |  |
| **ssps666** | F:GCGCACACATACACACACAC | 240 | 19 | AT | scaffold189 |
|  | R:TCTCACCAGGAGATGTGCAG |  |  |  |  |
| **ssps668** | F:CGAGAGCCCATTCAACACTT | 264 | 10 | CT | scaffold201 |
|  | R:CGCGCAAATCAAACTACAGA |  |  |  |  |
| **ssps670** | F:CTAACCGATCCAAACCGAAA | 249 | 14 | AT | scaffold208 |
|  | R:ATTCGCAGACGGATGGAGTA |  |  |  |  |
| **ssps671** | F:CTTGCTGAAGCCCAGAAATC | 166 | 11 | GA | scaffold200 |
|  | R:CAGGCTTGCTATTGCACAAA |  |  |  |  |
| **ssps674** | F:AGCAGGAATCTCGGAAGTCA | 179 | 18 | AT | scaffold220 |
|  | R:TATGGGAGCAGCTGAACGTA |  |  |  |  |
| **ssps678** | F:CATTCCCTTCATTCCCCATT | 194 | 18 | TA | scaffold213 |
|  | R:GTGGCAAAATCTGGACCACT |  |  |  |  |
| **ssps679** | F:GCATTTGGGAATTGGGAATA | 197 | 10 | CT | scaffold222 |
|  | R:CGCCATCTTCAACACAAAGA |  |  |  |  |
| **ssps680** | F:CCTCTCCGATTCCAACAAAA | 241 | 17 | AC | scaffold222 |
|  | R:CCTCATCTTCGTCCTCTTCG |  |  |  |  |
| **ssps682** | F:GAACAAACGATGAGGGAGGA | 126 | 14 | TA | scaffold227 |
|  | R:AATGATGTTGTTCGGCGTTT |  |  |  |  |
| **ssps684** | F:TCCTCACTCAAATCCCATCC | 232 | 15 | AC | scaffold224 |
|  | R:CAGCAGCAGTAGCAGATTCG |  |  |  |  |
| **ssps687** | F:GATCGTCCATCCCTCAAATC | 239 | 12 | TA | scaffold232 |
|  | R:GGATTCAGTTGCCACAGCTT |  |  |  |  |
| **ssps688** | F:CCACACCAACAAAACCATCA | 179 | 10 | AC | scaffold229 |
|  | R:TCGACCGCCATTTCTTTAAC |  |  |  |  |
| **ssps690** | F:CCGTGTTTTCAAATCCGTGT | 191 | 11 | TC | scaffold223 |
|  | R:GCGCTTGGATCTTAATCTCG |  |  |  |  |
| **ssps691** | F:GCGCTTGGATCTTAATCTCG | 175 | 10 | AT | scaffold237 |
|  | R:TCGATCAAGTTTGGTGGTGA |  |  |  |  |
| **ssps692** | F:CATTCTTGGCCATCTCGATT | 237 | 11 | AG | scaffold410 |
|  | R:AAGTCTGCATTTTGGGGTTG |  |  |  |  |
| **ssps693** | F:TACCAGCACTGGCAATGAAA | 203 | 10 | AC | scaffold246 |
|  | R:TTGAGGCATGTCCAACTGTC |  |  |  |  |
| **ssps694** | F:TCAACACTTGATCGCCACAT | 209 | 12 | CT | scaffold245 |
|  | R:GCACGGCAAACCCTAGTATC |  |  |  |  |
| **ssps698** | F:CTGCTCAATCCGCTAAGGAA | 217 | 15 | ATT | scaffold242 |
|  | R:AATTCTTGTTGACGGCATCC |  |  |  |  |
| **ssps699** | F:CTTCTCCTCGAACAGCGAGT | 153 | 10 | ATC | scaffold251 |
|  | R:GAACCCATCCTCCTCATTCA |  |  |  |  |
| **ssps702** | F:TCATTCATCAACGGTCCAGA | 264 | 20 | TA | scaffold270 |
|  | R:CAACTCGCAACCACTTAGCC |  |  |  |  |
| **ssps704** | F:TGAACACCTCGGACAAACAA | 214 | 13 | GA | scaffold259 |
|  | R:TGCAGAGCATGTTCATGGAT |  |  |  |  |
| **ssps706** | F:GGTGGAAGGGAAGGAAAAGA | 284 | 11 | TA | scaffold266 |
|  | R:CCGTCTTGGGAAAATGACAC |  |  |  |  |
| **ssps707** | F:TTTCGTCCGTCCCTAAAGTG | 219 | 10 | CT | scaffold275 |
|  | R:CCGTCCCCAAAGAGTATGAA |  |  |  |  |
| **ssps709** | F:AAGAAGCGCCATTCATTTTG | 140 | 10 | GA | scaffold279 |
|  | R:TGGTGCGAAATCTTCAGCTA |  |  |  |  |
| **ssps712** | F:GGTGATGGATTCTGGCATTC | 216 | 17 | GA | scaffold277 |
|  | R:CCACACCACAAACCTCTTCC |  |  |  |  |
| **ssps714** | F:CAATAGTTCTGTGGGGTGGA | 274 | 11 | TA | scaffold277 |
|  | R:GCTTTGCTACAGTTGCTTGC |  |  |  |  |
| **ssps715** | F:GCTCCAAATCGAGAAGCAAC | 273 | 18 | TA | scaffold294 |
|  | R:AGAAAGCCAGGAACCTACCC |  |  |  |  |
| **ssps716** | F:AGCCGAAGGTTCTGAAGACA | 139 | 10 | TG | scaffold309 |
|  | R:TGGAATGGAACTGAGGGAGT |  |  |  |  |
| **ssps717** | F:TTTGTGCTATCGTGCTTCCA | 222 | 18 | AT | scaffold299 |
|  | R:GAGGAATCAAAGCCCACGTA |  |  |  |  |
| **ssps718** | F:GTGGGGCTGAGATCAAGAAG | 264 | 11 | TA | scaffold310 |
|  | R:ACTTATCGAACGCGGAAAAG |  |  |  |  |
| **ssps719** | F:GACCGCAAAATCAAGGAAAA | 220 | 11 | GA | scaffold312 |
|  | R:GGTGATAGGTATGGCGGAGA |  |  |  |  |
| **ssps720** | F:CCAAATCCCCTTCAATTCCT | 270 | 10 | CTT | scaffold317 |
|  | R:CCGGAACAAACGAGATCAGT |  |  |  |  |
| **ssps723** | F:GCTGGCAACATAGTTGGTGA | 260 | 12 | TAA | scaffold340 |
|  | R:GGTAGGGCCACAAAATTGAC |  |  |  |  |
| **ssps725** | F:GGAAGGAAGAGAGGGAATGG | 221 | 11 | TC | scaffold350 |
|  | R:ATGCGTGGATAAGTGGGAAC |  |  |  |  |
| **ssps729** | F:CGTCCGTCCCAACTTAAGAG | 179 | 11 | AT | scaffold374 |
|  | R:AATGATGTTGTTCGGCGTTT |  |  |  |  |
| **ssps731** | F:TAATGCAACACGGAACCAGT | 196 | 15 | TA | scaffold389 |
|  | R:TTGTTCGGCGTTTAAGGTTT |  |  |  |  |
| **ssps733** | F:CTGCCCAAGATTTCCATTGT | 196 | 10 | TA | scaffold406 |
|  | R:ACATATTTACCCCGCGCTTT |  |  |  |  |
| **ssps735** | F:TCAGTGTCCTCGCATACACC | 262 | 19 | TA | scaffold512 |
|  | R:AAGCACCAATCATCCCTTTG |  |  |  |  |
| **ssps738** | F:GTAGCGGTTTCCAGCCATTA | 233 | 13 | AT | scaffold573 |
|  | R:GTCAGCACACTAACGCAACC |  |  |  |  |
| **ssps739** | F:TTTGGTTAAGGGGTGTTTGC | 142 | 17 | TA | scaffold799 |
|  | R:CGTGGGGCTGAGATTAAGAA |  |  |  |  |
| **ssps740** | F:GAACAAACGATGAGGGAGGA | 164 | 19 | TA | scaffold812 |
|  | R:TGAGTGAGGTTTGCCTGAGA |  |  |  |  |
| **ssps744** | F:CTGATGTTGCACTTGCCATT | 231 | 11 | TTA | scaffold4 |
|  | R:GGATATTCCCGTGCAATGTT |  |  |  |  |
| **ssps745** | F:CGTGTGCAGTGTGTGAAATG | 134 | 12 | GT | scaffold4 |
|  | R:AGCGCAACCTATTGAGCCTA |  |  |  |  |
| **ssps746** | F:GATGGTCGCACTTTCTGGTT | 226 | 10 | AAT | scaffold4 |
|  | R:GCAGAAGTTCCCTGTCAAGC |  |  |  |  |
| **ssps747** | F:AGCGCAATTAGAGTGGATGC | 175 | 14 | AG | scaffold4 |
|  | R:GGGGTTGCTTCGTTTTGTTA |  |  |  |  |
| **ssps748** | F:AACCGTCGGATGACAAAATC | 218 | 14 | AT | scaffold4 |
|  | R:AAGTCCTTCGCAGCTTTTCA |  |  |  |  |
| **ssps749** | F:TAGGGCCAAGATTTGGTCTG | 137 | 16 | TA | scaffold4 |
|  | R:CCCCATTGCGGATACTCTAA |  |  |  |  |
| **ssps750** | F:TAAGGTCTAGGCCGTGGTGT | 264 | 11 | GA | scaffold4 |
|  | R:GAGAGCGAGAGAGAGCGATG |  |  |  |  |
| **ssps751** | F:TAAGGTCTAGGCCGTGGTGT | 297 | 17 | AG | scaffold4 |
|  | R:AAGAGAGAGCGCGAGAGATG |  |  |  |  |
| **ssps752** | F:CCAGAAATCCCCAATCAGAA | 164 | 11 | TC | scaffold4 |
|  | R:CCGAATCAACAAATCCCAGT |  |  |  |  |
| **ssps753** | F:TTGCCTGGATCACGCTAAAT | 215 | 17 | AC | scaffold4 |
|  | R:ATGCTGCTGTGTGTTTCTGG |  |  |  |  |
| **ssps754** | F:TTTCCTTGTAAGGGCAATCA | 203 | 17 | TTA | scaffold921 |
|  | R:ACCTTTGGAAAATGGCTTTG |  |  |  |  |
| **ssps755** | F:TGCTGAAACAACCAATTCCA | 202 | 10 | TAA | scaffold929 |
|  | R:TTCTCCTATCAACGCGGTTT |  |  |  |  |
| **ssps756** | F:TTGGAAATTTGAAGGAGACG | 273 | 17 | TA | scaffold970 |
|  | R:GCTGCCAACACCTCATAAAC |  |  |  |  |
| **ssps759** | F:TCGGCAAAATCCTACCAAAG | 281 | 12 | AT | scaffold1245 |
|  | R:ACGACCTCCGTTTGACATTT |  |  |  |  |
| **ssps760** | F:CCGGCAAACTCTTCGACTAC | 126 | 10 | TC | scaffold1241 |
|  | R:TTAACCTCCCAAACCACACC |  |  |  |  |
| **ssps761** | F:CCATGCTTTCCATTTCACCT | 132 | 10 | CT | scaffold1269 |
|  | R:AGACCCACGGAGACGTTATG |  |  |  |  |
| **ssps763** | F:TGAGACCTTTGTCTGCACCA | 129 | 11 | AG | scaffold2 |
|  | R:GACACCAGCCGCTAATTTTC |  |  |  |  |
| **ssps764** | F:GCGACATTTCTTCGTCACAC | 261 | 14 | CT | scaffold2 |
|  | R:GGATCAACCCCACCCTAAAT |  |  |  |  |
| **ssps765** | F:GATCGTTTTAGGTCATGCTTTG | 268 | 19 | TA | scaffold2 |
|  | R:TCTTTCAAACTGAGTGGCCATA |  |  |  |  |

**Table S3.** Characteristics of 271 polymorphic SSR markers for *S. splendens*.

| **Locus** | **Sequence (3' to 5')** | **Product size** | **Repetitions** | **Motif** | **Scaffold** |
| --- | --- | --- | --- | --- | --- |
| **ssps195** | F: TCTGCGTACGAGTCTCCAAA | 254 | 10 | TA | scaffold14 |
|  | R: CAAGGTGGGGCAATAAAGAT |  |  |  |  |
| **ssps196** | F: GACACAATTGGAAGCAAGCA | 157 | 10 | AC | scaffold1458 |
|  | R: TGGACCAAGAAGGAATCTGG |  |  |  |  |
| **ssps197** | F: AAACTCCGGCACCGTCTACT | 147 | 11 | TCT | scaffold40 |
|  | R: CTGGGGTTGGTTGATAATGC |  |  |  |  |
| **ssps198** | F: GCAGCTGACACAGAACCAGA | 267 | 15 | TTA | scaffold17 |
|  | R: CAGACAAGCAGGGTGAACAA |  |  |  |  |
| **ssps201** | F: CAGCCAATGAGAAGCATGAA | 238 | 12 | AC | scaffold17 |
|  | R: TTGGTTCGTGCCATTGTAGA |  |  |  |  |
| **ssps203** | F: TCTTGCCCAATTCATTCTCC | 201 | 14 | TTC | scaffold1023 |
|  | R: CAACGTCCATCTCTGTCGAA |  |  |  |  |
| **ssps204** | F: ATGGTCAGGATTTCGTCTGG | 157 | 19 | TA | scaffold19 |
|  | R: CCTAGTTCCTGTCCCCAAAA |  |  |  |  |
| **ssps205** | F: AAGTTGTGGAGAGGCATTGG | 136 | 13 | AT | scaffold19 |
|  | R: ACATGCACATGCCGAATAAA |  |  |  |  |
| **ssps206** | F: AATTTGAGGTTGGGTGCAAG | 186 | 10 | CT | scaffold19 |
|  | R: CCATCCCAAACCCTTCTCTA |  |  |  |  |
| **ssps207** | F: TGCATCAGCAATTCCCAATA | 253 | 10 | CT | scaffold19 |
|  | R: GATGAAGCCAGAAGCAGAGC |  |  |  |  |
| **ssps208** | F: ACAGTAAAACGACCGGACCA | 187 | 20 | ATT | scaffold19 |
|  | R: TCAATGGGACGAAGGGAGTA |  |  |  |  |
| **ssps209** | F: CGACTCCATCGAACACTGAA | 254 | 19 | TC | scaffold19 |
|  | R: TCTCTCACTCTCACCGCAGA |  |  |  |  |
| **ssps210** | F: CACGACCTCAAACCCAAAGT | 270 | 10 | TA | scaffold19 |
|  | R: GGTCCATCGCAAGGAATTTA |  |  |  |  |
| **ssps211** | F: GCAATTGAGCCCATTACTGA | 247 | 13 | TC | scaffold19 |
|  | R: CCCAATTGAAGAAGGACTGC |  |  |  |  |
| **ssps212** | F: GCCGTTGATGAAAATCACCT | 235 | 10 | GT | scaffold19 |
|  | R: CATGGGAACCGTTATCGACT |  |  |  |  |
| **ssps213** | F: CCAGGATGTCATTGTTCGAG | 172 | 13 | TG | scaffold19 |
|  | R: GGGTTTGATTCCGTTCCATA |  |  |  |  |
| **ssps214** | F: AGGGAAAAGAAAAGGCAAGC | 268 | 11 | AG | scaffold19 |
|  | R: TACTCCACCTGGGCTAGTCG |  |  |  |  |
| **ssps222** | F: CGCCATTGGAGATGCTCTTA | 200 | 10 | GA | scaffold6 |
|  | R: AGCGGAGACGGAGAGATTTT |  |  |  |  |
| **ssps224** | F: TTGCACGAACCAAATCACAT | 173 | 10 | AG | scaffold6 |
|  | R: AGTGCGATTGCAGAGTGAGA |  |  |  |  |
| **ssps237** | F: TCGCCCAATTTCTTTAATCG | 268 | 10 | TA | scaffold20 |
|  | R: AAGCTACATGGCTGCTGTGA |  |  |  |  |
| **ssps238** | F: GTTCGAAACAAGCTGGCATT | 296 | 13 | GA | scaffold20 |
|  | R: CGGGAGGGTCTATGTACCAA |  |  |  |  |
| **ssps240** | F: GGGCAAGGTTGAATTGAAGA | 162 | 11 | GA | scaffold20 |
|  | R: ACAAAACAAGGCCTCAAACG |  |  |  |  |
| **ssps243** | F: AGGACACCAACCCATCAAAG | 295 | 11 | AG | scaffold20 |
|  | R: AGAAAGGGCAAAACAAAGCA |  |  |  |  |
| **ssps244** | F: GCGACAGGTTCCAATGAGAT | 217 | 11 | AG | scaffold20 |
|  | R: GGGTGGTCAGCCTTGTTCTA |  |  |  |  |
| **ssps250** | F: CCCGCCTTATTATTGTGGAG | 202 | 11 | TA | scaffold12 |
|  | R: CCCATGGAACCCCTAAGTTT |  |  |  |  |
| **ssps251** | F: GACGCTCAAATGGTGAATCC | 219 | 11 | TC | scaffold12 |
|  | R: ACGCTGATCTGGAAAGATGG |  |  |  |  |
| **ssps254** | F: AAGAGGCCTTAGCCCATAGC | 165 | 17 | TA | scaffold12 |
|  | R: AAAGGATGAAGAGCCCCAAG |  |  |  |  |
| **ssps255** | F: CGGACGGAATTCATAATGGT | 270 | 10 | ATT | scaffold12 |
|  | R: CTTGGAGAAGGCCAAGTCTG |  |  |  |  |
| **ssps258** | F: ACCCAAATCAAGAATGCACA | 277 | 13 | TTA | scaffold7 |
|  | R: ACCGAACTTCAGACCCAAAC |  |  |  |  |
| **ssps270** | F: TTCAACACCCACACGTTCTC | 227 | 15 | TA | scaffold16 |
|  | R: CTTAACCCGCAAGCTCTGTT |  |  |  |  |
| **ssps272** | F: CGGCATAATGGGCTGTTTAG | 232 | 15 | TC | scaffold16 |
|  | R: TCTGTGATGGGCTTCTCTCC |  |  |  |  |
| **ssps274** | F: CAGCAATTTGGGTTCCTTGT | 212 | 19 | AG | scaffold16 |
|  | R: GATGGCTTGAGGTTGGATGT |  |  |  |  |
| **ssps279** | F: CAACGTCCATCTCTGTCGAA | 190 | 10 | GAA | scaffold211 |
|  | R: TCTTGCCCAATTCATTCTCC |  |  |  |  |
| **ssps284** | F: TCATTCATCAACGGTCCAGA | 242 | 10 | TA | scaffold15 |
|  | R: GAATGCTTTTGCATGGATCA |  |  |  |  |
| **ssps285** | F: CGCACAAACAGAAATTCCAA | 217 | 12 | CA | scaffold15 |
|  | R: GGAGGAGGAAACCGAGTACC |  |  |  |  |
| **ssps286** | F: ACACCATTCCATTTCGCTCT | 240 | 15 | CT | scaffold15 |
|  | R: TTGCGAAGACGAATACATGC |  |  |  |  |
| **ssps287** | F: AAGCCAAGCGTGTGTATCCT | 229 | 10 | TG | scaffold15 |
|  | R: TTGGCTGCTTGATTCATTTG |  |  |  |  |
| **ssps288** | F: ATCAGGCATGAGCACAAACA | 247 | 11 | AGA | scaffold15 |
|  | R: TTGGGAGGAAGAATGAATCG |  |  |  |  |
| **ssps290** | F: GCCTAAGAAAGTTGCCTCCA | 205 | 16 | CA | scaffold29 |
|  | R: ACAATGACCCTCCGTGTTTT |  |  |  |  |
| **ssps291** | F: GTCACTCATCACCGTCTCCA | 200 | 12 | TA | scaffold29 |
|  | R: TGCAATGCCTGTGAAAGAAG |  |  |  |  |
| **ssps292** | F: GCGAGACATCCGAAATTGAT | 230 | 10 | TA | scaffold29 |
|  | R: ACCAAATCTGCCCTTAATCG |  |  |  |  |
| **ssps293** | F: GACGGCTCCTCCAATGTTAT | 277 | 11 | TG | scaffold29 |
|  | R: TGGCATGACAATAGGGAAAA |  |  |  |  |
| **ssps294** | F: ATGGGAGAGAGGGAAGGATG | 235 | 19 | ATT | scaffold44 |
|  | R: AAGCGGGTACTTCAACATGG |  |  |  |  |
| **ssps296** | F: GGCAGATGCAAGAACAGGAT | 200 | 14 | GA | scaffold29 |
|  | R: TCCAAAGAGTGGCAACATCA |  |  |  |  |
| **ssps298** | F: ACGCAAATGGCAACCTAGAC | 251 | 11 | TC | scaffold42 |
|  | R: CCACGGTAGGCAAGTTTTCA |  |  |  |  |
| **ssps299** | F: AAGGGCTACACGAGACGTTG | 283 | 18 | TA | scaffold10 |
|  | R: GGGAAGAAGTTGCATCGGTA |  |  |  |  |
| **ssps301** | F: CAGAGCTCGCAATCAATCAA | 251 | 17 | AC | scaffold10 |
|  | R: ATCCTTTGGATTCCGTCACA |  |  |  |  |
| **ssps302** | F: AACGGTTGATGCTGTGTTGA | 221 | 13 | TA | scaffold10 |
|  | R: ATGCCTAATTCCAAGGCAAA |  |  |  |  |
| **ssps303** | F: TCCTGCAGATCAAACACTCG | 142 | 12 | AG | scaffold10 |
|  | R: AATGCTCGACGCTTTAATGG |  |  |  |  |
| **ssps304** | F: CCCCTGAAATCATCGAAATG | 228 | 11 | TC | scaffold10 |
|  | R: CGGAGAGGAGGAGAGGAAAA |  |  |  |  |
| **ssps305** | F: TACTATGCCTCGCCTCATCC | 266 | 19 | AT | scaffold10 |
|  | R: TGGTAAGGCACGTGAACAAA |  |  |  |  |
| **ssps308** | F: AATTGGGTGGGTAAGTGACG | 289 | 11 | TA | scaffold10 |
|  | R: CTTTGCTTTTGGGAGCTTTG |  |  |  |  |
| **ssps309** | F: TTTTGGTTCGGCTCTGTTTT | 181 | 13 | TA | scaffold10 |
|  | R: GGGCCAAGTTGTTGATGAAT |  |  |  |  |
| **ssps314** | F: GTACGCCTTCCAACACCCTA | 294 | 15 | CT | scaffold33 |
|  | R: GCAAGGCTATCGACATCTCC |  |  |  |  |
| **ssps315** | F: CCGTTTTGTTGGGAGAAATG | 221 | 20 | AT | scaffold28 |
|  | R: GAATGCTTTTGCATGGATCA |  |  |  |  |
| **ssps318** | F: GTTGGGAAAATTTGGTGGTG | 172 | 11 | CT | scaffold28 |
|  | R: TGCTTGTGGTGGTAGAGTGG |  |  |  |  |
| **ssps321** | F: ACGTGAACACACACCACCAT | 193 | 10 | GCT | scaffold31 |
|  | R: ACAGCATCAGCAACAGCAAC |  |  |  |  |
| **ssps322** | F: GGATCCAAGGGCTGTGATTA | 210 | 15 | AT | scaffold25 |
|  | R: GTGGCGGTGATTTGTTCTCT |  |  |  |  |
| **ssps324** | F: AAAATTCCCTGGATGCCTCT | 212 | 15 | CT | scaffold25 |
|  | R: TGAAATTGTGGTGGTGTTGC |  |  |  |  |
| **ssps325-1** | F: ACTTGCGAACGTACCCTTTT | 221 | 13 | TTA | scaffold25 |
|  | R: TCGGAGTCGAAATAGCAACA |  |  |  |  |
| **ssps325-2** | F: ACTTGCGAACGTACCCTTTT | 221 | 13 | TTA | scaffold25 |
|  | R: TCGGAGTCGAAATAGCAACA |  |  |  |  |
| **ssps328** | F: GCACCATGCTGTAGATCCAA | 168 | 13 | CT | scaffold32 |
|  | R: TGAATCAATCCCACTGCAAA |  |  |  |  |
| **ssps329** | F: AACCGTCGGATGACAAAATC | 144 | 11 | AT | scaffold32 |
|  | R: AAATTTTCAGGGACGGAGGT |  |  |  |  |
| **ssps330** | F: AGAGGTCGCACGATTCCTAA | 175 | 19 | TTA | scaffold32 |
|  | R: TGGTATAGGGCAGGCTATGG |  |  |  |  |
| **ssps331** | F: AAATCGTGGGGCTGAGATTA | 143 | 11 | TA | scaffold32 |
|  | R: ATGGACCACACATTCCCCTA |  |  |  |  |
| **ssps334** | F: CGCTAGAGATGGGTCGAGAA | 221 | 10 | TG | scaffold34 |
|  | R: TTCTACCGCTCATGTTGCTG |  |  |  |  |
| **ssps336** | F: AAACGGAAACCGAATGTCTG | 247 | 17 | TA | scaffold30 |
|  | R: TCCAAAACCAGTGCTCAAAA |  |  |  |  |
| **ssps337** | F: TGCTGCAATCCATAGGAAAG | 147 | 10 | AC | scaffold30 |
|  | R: AATTTGGCTCCCAATAGCTG |  |  |  |  |
| **ssps338** | F: GGCAGCAAGTCCACAAATCT | 239 | 11 | CT | scaffold30 |
|  | R: TCCCACCACTTTCTTCCAAC |  |  |  |  |
| **ssps339** | F: CGACTATGCAGCCTAACCAA | 292 | 16 | ATA | scaffold30 |
|  | R: CCCATCCATTCCATTCACTT |  |  |  |  |
| **ssps343** | F: CTCGGGAGAGTATCGTTTCG | 234 | 15 | TCT | scaffold27 |
|  | R: CCACCAGAGTCTCCTCAACC |  |  |  |  |
| **ssps344** | F: CCTTCACCTGGGATGGAGTA | 277 | 10 | CT | scaffold27 |
|  | R: GCCCTTTCAACCAAAACAAA |  |  |  |  |
| **ssps347** | F: GGCCTGAAGGAGCTGTAATG | 166 | 12 | GA | scaffold27 |
|  | R: TGCACGCCTCAACTACAAAC |  |  |  |  |
| **ssps348** | F: GCCGCATACCAGATACCTCT | 125 | 12 | CA | scaffold27 |
|  | R: ATGCGACATGAAACAGAAGC |  |  |  |  |
| **ssps357** | F: GCCATTTGAAACGAGAGCTT | 211 | 11 | GT | scaffold36 |
|  | R: CGGATTTCCAATCACAGCTT |  |  |  |  |
| **ssps358** | F: ATTCCCAGCCACTGCTAATG | 265 | 19 | TAT | scaffold23 |
|  | R: TTTTCGGCACGGAGATTAAG |  |  |  |  |
| **ssps359** | F: TGACAAAATCGTGGAGCTGA | 233 | 18 | AT | scaffold23 |
|  | R: GCAACACGGATACACATTGG |  |  |  |  |
| **ssps361** | F: AAATCACCACCACCGTCACT | 168 | 13 | AT | scaffold23 |
|  | R: TTTAAGCTTTCCGCACCTTG |  |  |  |  |
| **ssps364** | F: AAGCCACCCTTTTCATTTCA | 239 | 11 | CA | scaffold23 |
|  | R: CGAGTCTTTTGCCCACTTTT |  |  |  |  |
| **ssps368** | F: AGAGTCAGCCAGGGAGGTTT | 136 | 10 | GA | scaffold23 |
|  | R: TTACCGACTGATGGCTCCTC |  |  |  |  |
| **ssps370** | F: CGCACCAACCACATGATCTA | 229 | 17 | TA | scaffold1 |
|  | R: ACATCACCTTCCCAATTCCA |  |  |  |  |
| **ssps373** | F: GCTTGATTGTGGGTTTGGTT | 193 | 13 | AT | scaffold1 |
|  | R: GCGGGTTTGACAGAGAGAGA |  |  |  |  |
| **ssps374** | F: GGAATTGCCGATTACCTGAA | 257 | 10 | AT | scaffold1 |
|  | R: GACCCAATCACCGATAAGGA |  |  |  |  |
| **ssps377** | F: CACACAATCACCGCAGACTT | 280 | 10 | AG | scaffold1 |
|  | R: CATCGCGCTTCACTTATCAA |  |  |  |  |
| **ssps378-1** | F: ACCGATACCTTTTGGGTCGT | 250 | 16 | TTA | scaffold1 |
|  | R: CGCAGCTAACAACGAGATGA |  |  |  |  |
| **ssps378-2** | F: ACCGATACCTTTTGGGTCGT | 250 | 16 | TTA | scaffold1 |
|  | R: CGCAGCTAACAACGAGATGA |  |  |  |  |
| **ssps380** | F: CCTTTCGGAGGATGGTCTCT | 208 | 12 | TC | scaffold1 |
|  | R: GGCGAGCGTACCATAAACAT |  |  |  |  |
| **ssps381** | F: CCTTGGCCTCTAAACAAGGA | 127 | 12 | AT | scaffold24 |
|  | R: TGGCTACCAATCCAACACAC |  |  |  |  |
| **ssps383** | F: CTCCATGGGAGGGTGTAGAA | 210 | 20 | GA | scaffold24 |
|  | R: CTGTTTGTGAGTGGCAGCAT |  |  |  |  |
| **ssps385** | F: CGCCAGAGCGTAGATTTCTT | 287 | 14 | TTA | scaffold24 |
|  | R: GCTCCAACTTCAGTGCATCA |  |  |  |  |
| **ssps387** | F: GCCACCGTAAAATCAAAAGC | 176 | 10 | AG | scaffold24 |
|  | R: CTCCCAAACCGATGTTGTCT |  |  |  |  |
| **ssps388** | F: GCCCTAGAAATTGGGAGAGG | 190 | 10 | TC | scaffold24 |
|  | R: CTGCAACCACCATCTGCTTA |  |  |  |  |
| **ssps389** | F: CAGAGGGTTCAAGCATCCAT | 185 | 12 | AC | scaffold24 |
|  | R: ACTCCCTCCCCTCCTTACAA |  |  |  |  |
| **ssps390** | F: TCCCCTCCTCTTTCCCTAAA | 225 | 10 | AC | scaffold40 |
|  | R: CCCCACTGCTCATCTTCTTC |  |  |  |  |
| **ssps391** | F: CGCTCCACCTTCCTTTATCA | 280 | 16 | CT | scaffold40 |
|  | R: GACACCAGCAACGAAGCATA |  |  |  |  |
| **ssps393** | F: CACATGTAATGGGACGGATG | 271 | 17 | AT | scaffold40 |
|  | R: ATGAGTCCGAGCAGAGCATT |  |  |  |  |
| **ssps394** | F: GCATACCAACATGGGAGCTT | 260 | 13 | GA | scaffold40 |
|  | R: CAACAAACATGGCCACACTC |  |  |  |  |
| **ssps395** | F: AATTGCTCATGGAAGGCTGT | 237 | 15 | GT | scaffold42 |
|  | R: AGCAATGGAGGTCCAAAAGA |  |  |  |  |
| **ssps396** | F: GCAATTTCGATGTTGGTGAA | 239 | 19 | TG | scaffold42 |
|  | R: CACACACACACACTGGCTGA |  |  |  |  |
| **ssps399** | F: GCCACCTGCAAATAGGAGAA | 192 | 11 | GA | scaffold39 |
|  | R: ACCCCAGAACAGGAAGAGGT |  |  |  |  |
| **ssps401** | F: TCCTGTGTTTGTTTCGGTTT | 244 | 11 | TTA | scaffold128 |
|  | R: TCCTGAAATTAGGCATGGTT |  |  |  |  |
| **ssps404-1** | F: TTGCCAGGAAATCCAAAATC | 299 | 11 | TA | scaffold9 |
|  | R: GGTGCATTATTAGCCGGTGT |  |  |  |  |
| **ssps404-2** | F: TTGCCAGGAAATCCAAAATC | 299 | 11 | TA | scaffold9 |
|  | R: GGTGCATTATTAGCCGGTGT |  |  |  |  |
| **ssps405** | F: CCAAGTCTGTGAATGCCAAA | 298 | 11 | TC | scaffold9 |
|  | R: TCATTCCCACATCAAAAGCA |  |  |  |  |
| **ssps407** | F: GCCATGGACTGGTTTGAAGT | 231 | 11 | GAT | scaffold9 |
|  | R: TTCTCCTCCCCAATATGTCG |  |  |  |  |
| **ssps410** | F: TGAGGTGCATTGGATACTCG | 295 | 10 | AC | scaffold9 |
|  | R: AAGCGACACACACACACACA |  |  |  |  |
| **ssps411** | F: GTTCCCCACACCAATCAAAC | 202 | 10 | TA | scaffold9 |
|  | R: GGAGAGGAACCGTCGTATCA |  |  |  |  |
| **ssps426** | F: TGGCCCAGATTTGGTCTCTA | 270 | 20 | TA | scaffold45 |
|  | R: AAAGGATTGGGAAGCAGTGA |  |  |  |  |
| **ssps427** | F: ACACACACACACCCCAATGT | 262 | 12 | AC | scaffold45 |
|  | R: GCACAAAGGGCATCAGTCTT |  |  |  |  |
| **ssps428** | F: AGATGCGAAAACCCAAACTG | 151 | 11 | AG | scaffold41 |
|  | R: ACAGATCGCTGTCATTGTCG |  |  |  |  |
| **ssps429** | F: TTAGCCCTTTTCGGGTATTG | 206 | 17 | TA | scaffold41 |
|  | R: GTGGAGCACGAACTTGAGAA |  |  |  |  |
| **ssps430** | F: TATATAGCCGCGGGATGAAC | 197 | 13 | AT | scaffold41 |
|  | R: GATCGCTGGCACTTCTTTGT |  |  |  |  |
| **ssps433** | F: GCGACAGTGAGACAGATCCA | 212 | 11 | CA | scaffold50 |
|  | R: GACCCTCGACCCTAACAACA |  |  |  |  |
| **ssps434** | F: GGCGGACGACTGAAGTAGAG | 155 | 19 | AT | scaffold50 |
|  | R: AACCGTCGGATGACAAAATC |  |  |  |  |
| **ssps435** | F: AAGTCAGCCCAAGAGTGGAA | 165 | 20 | TTC | scaffold52 |
|  | R: GCACCCTAAAACGGCTAACA |  |  |  |  |
| **ssps441** | F: CGAAACGATCTCTTGTTCGAT | 199 | 14 | TTA | scaffold37 |
|  | R: TCGAGTAGTCCCAAATGTCCT |  |  |  |  |
| **ssps442** | F: GTTCGGTGGTTCGGTCTTTA | 270 | 11 | TA | scaffold53 |
|  | R: TCGTCAAATCGCAGTACGTC |  |  |  |  |
| **ssps445** | F: TTCTTCCCGATGGTGTTCTC | 221 | 13 | AG | scaffold3 |
|  | R: TATTTGGCTGCGTTTTGACC |  |  |  |  |
| **ssps446** | F: TATTTGGCTGCGTTTTGACC | 223 | 14 | CT | scaffold3 |
|  | R: TTCTTCCCGATGGTGTTCTC |  |  |  |  |
| **ssps447** | F: TTCCGGACACTCGACCTATC | 143 | 10 | CA | scaffold3 |
|  | R: GGCAAGGGAAGAGGCTAAAG |  |  |  |  |
| **ssps448** | F: CCCACCATTCCATAAACACA | 188 | 11 | AC | scaffold3 |
|  | R: ATTTCCTCGGTGTTTGCATC |  |  |  |  |
| **ssps449** | F: GCCACGCTTTCTTTGTCATT | 261 | 11 | AG | scaffold3 |
|  | R: GCATTGTGATTTCGGAGGAT |  |  |  |  |
| **ssps451** | F: GACATCGTAGGCCGTGAAAT | 257 | 13 | AG | scaffold133 |
|  | R: ACCGCCATGAGAGAGAAGAA |  |  |  |  |
| **ssps452** | F: TCGAACCAGATAATGCATCG | 264 | 20 | TC | scaffold3 |
|  | R: GATGAGCGGAACCACAGTTT |  |  |  |  |
| **ssps453** | F: TGCTGCAAAACAAAGGAATG | 180 | 11 | AG | scaffold3 |
|  | R: GCCAATAACTGGGTTGGATG |  |  |  |  |
| **ssps459** | F: CGGCGAGAGTGATGTAGATG | 225 | 11 | GA | scaffold56 |
|  | R: TCACGGCTCTCTTCACACAG |  |  |  |  |
| **ssps460** | F: CCCAAAGAAAGCCGGTAACT | 184 | 12 | CT | scaffold56 |
|  | R: CCTCCTCGTCTTCGCTTATG |  |  |  |  |
| **ssps462-1** | F: CCCTGCTCAGAAGCAAAGAA | 253 | 11 | CA | scaffold56 |
|  | R: ACAGATACGCCACCTGATCC |  |  |  |  |
| **ssps462-2** | F: CCCTGCTCAGAAGCAAAGAA | 253 | 11 | CA | scaffold56 |
|  | R: ACAGATACGCCACCTGATCC |  |  |  |  |
| **ssps466** | F: CCTGATAGCTGGAGCACACA | 268 | 13 | AG | scaffold48 |
|  | R: GGTTTTCATTTTGCCTCTGC |  |  |  |  |
| **ssps467** | F: TCCCATGAGGGAGAAAACAT | 249 | 12 | GT | scaffold48 |
|  | R: CCACACACAAAGAGGTGGTG |  |  |  |  |
| **ssps469** | F: TGGATTGATTTGTGGTGGAA | 156 | 20 | TA | scaffold48 |
|  | R: GCAAACCAAAGAGGTCCAGA |  |  |  |  |
| **ssps471** | F: TATGCAGTCGTTGGAAGCAG | 236 | 10 | AG | scaffold48 |
|  | R: GTTCCGATGACCGTCTTGTT |  |  |  |  |
| **ssps476** | F: GCTCCACGGTCCTTTAGTTG | 201 | 13 | GA | scaffold73 |
|  | R: GCAGCCTGTTGGTTTAGGAA |  |  |  |  |
| **ssps488** | F: AAATTCGGGTTTGAGGTCGT | 218 | 11 | TA | scaffold71 |
|  | R: CGCCCAGCATATCATCTCTC |  |  |  |  |
| **ssps492-1** | F: CAGTTGGCCGTATACCTTCC | 174 | 19 | TC | scaffold74 |
|  | R: CACCCAGATACCTGCCAAAT |  |  |  |  |
| **ssps492-2** | F: CAGTTGGCCGTATACCTTCC | 174 | 19 | TC | scaffold74 |
|  | R: CACCCAGATACCTGCCAAAT |  |  |  |  |
| **ssps493** | F: TGTCCTCACAAAGCAATCCA | 127 | 11 | GA | scaffold74 |
|  | R: CGGACTCAGCAATACGAACA |  |  |  |  |
| **ssps495** | F: ATATGCGCCCACACTTGTTT | 260 | 10 | TC | scaffold74 |
|  | R: CAAAAGCTAGCCCAAAGGTG |  |  |  |  |
| **ssps496** | F: TCCCTTCGTCTCCGTCAATA | 287 | 10 | AC | scaffold62 |
|  | R: GTTCAGGTCTCCGAGGAAGG |  |  |  |  |
| **ssps500** | F: CCCATCAACCACCACTTTTC | 274 | 12 | CT | scaffold75 |
|  | R: CTGCCCCTCAATGTACCACT |  |  |  |  |
| **ssps501** | F: CCGCCCATAGTCAGATCCTA | 163 | 14 | AT | scaffold68 |
|  | R: CAAGAAAAGGCATGCGTACA |  |  |  |  |
| **ssps502** | F: GCTGGGGACAAAGAATGAAA | 157 | 18 | AC | scaffold68 |
|  | R: TGTCACGTGCGATCTCTCTC |  |  |  |  |
| **ssps505** | F: TTCCTCACCAATCCCTCATC | 199 | 11 | TC | scaffold61 |
|  | R: ACAACGGTGGAGAAAACTGC |  |  |  |  |
| **ssps507** | F: GCACACAGCAAGGTCTCTCA | 127 | 11 | TG | scaffold61 |
|  | R: AAGCAAACAACGCCCTAATG |  |  |  |  |
| **ssps508** | F: CGCGAAAATTGTGAATGAGT | 215 | 16 | AT | scaffold61 |
|  | R: ACAATTCCAAGTGGCTCCAT |  |  |  |  |
| **ssps510** | F: GCCGATGCAATTTGGTTCTA | 276 | 13 | CT | scaffold61 |
|  | R: AGAATCCATTCCCACCCAAC |  |  |  |  |
| **ssps511** | F: CCACCACCAATAGCCACAAT | 133 | 17 | GA | scaffold81 |
|  | R: GGATAACAGAGCAGGGATGC |  |  |  |  |
| **ssps512** | F: GGCTCCTCGTTTTATGGTGA | 235 | 11 | GA | scaffold81 |
|  | R: GCATTAGTTGATGCCGAGGT |  |  |  |  |
| **ssps514** | F: TGGCCCAGATTTGGTCTCTA | 207 | 11 | TA | scaffold63 |
|  | R: AGCCCATTCTGCCCTTTTAT |  |  |  |  |
| **ssps517** | F: CGGTTTTGTCGGTTTTGTTC | 213 | 14 | AT | scaffold80 |
|  | R: CTCGTGATGAAGGCGAAAAT |  |  |  |  |
| **ssps520** | F: CATGTTGAGCAGCAGCATTT | 151 | 13 | CT | scaffold80 |
|  | R: TCTTGGGAGTGGGTTTTGAG |  |  |  |  |
| **ssps523** | F: CAACGAAGGCGTAAAGGTGT | 127 | 14 | AGA | scaffold87 |
|  | R: GCATTCCACTCGAGCAATCT |  |  |  |  |
| **ssps530** | F: AGGAGGCTGATGATGGTGTC | 156 | 10 | TCC | scaffold78 |
|  | R: CCTGTGAATCTTGCTGACGA |  |  |  |  |
| **ssps531** | F: CGTTGACCTGCTCCGATATT | 246 | 15 | TA | scaffold77 |
|  | R: TGCACTCATGCACAAACTCA |  |  |  |  |
| **ssps535** | F: ACACTCGCCATTTTCATTCC | 142 | 15 | TC | scaffold8 |
|  | R: TGAATGTGCGACCAAGAGAG |  |  |  |  |
| **ssps537** | F: TTGTTGGCATGGATATGGTG | 184 | 11 | TC | scaffold8 |
|  | R: CAACCCATTCCTCGTAATCC |  |  |  |  |
| **ssps538** | F: CCTGCAAGTGTGTTGACGAA | 217 | 14 | ATT | scaffold8 |
|  | R: TTATCCATCCACCGATCCAC |  |  |  |  |
| **ssps539** | F: CCAGACTGCAAGCTTTGGTT | 228 | 15 | TA | scaffold8 |
|  | R: GGGTGTGATGGAGGAATTGT |  |  |  |  |
| **ssps543** | F: GCAGTTCATGGGTGGATTTT | 211 | 15 | TCA | scaffold8 |
|  | R: TGGATGGTGCTCAATCATGT |  |  |  |  |
| **ssps544** | F: CACAGCAGCACATCTCATCA | 159 | 17 | TA | scaffold8 |
|  | R: TTCGGGCTGTTTGTGTGTAA |  |  |  |  |
| **ssps546** | F: GCCTGTGCAAGGATAAAGGA | 274 | 11 | TA | scaffold8 |
|  | R: GGCTGAGATTTGGTCTGCAT |  |  |  |  |
| **ssps547** | F: GGCCCATGAGTGCAAAATAA | 151 | 15 | TC | scaffold8 |
|  | R: CGAAAATCTTCCGGATTTGA |  |  |  |  |
| **ssps548** | F: TGTGGAGTTGAGCTTGAGGA | 177 | 19 | TC | scaffold8 |
|  | R: CGCAACTCATTCCAATCAAA |  |  |  |  |
| **ssps549** | F: TCCTGAAATTAGGCATGGTT | 247 | 12 | TAA | scaffold187 |
|  | R: TCCTGTGTTTGTTTCGGTTT |  |  |  |  |
| **ssps553-1** | F: GCACACTAACGCAACCCAAT | 256 | 10 | TAT | scaffold88 |
|  | R: ATGGCCGCTCTGTAAAACTG |  |  |  |  |
| **ssps553-2** | F: GCACACTAACGCAACCCAAT | 256 | 10 | TAT | scaffold88 |
|  | R: ATGGCCGCTCTGTAAAACTG |  |  |  |  |
| **ssps554** | F: GCATGCAAGAGATTCCCACT | 235 | 17 | AC | scaffold90 |
|  | R: TACACATGGAAGCCCAACAA |  |  |  |  |
| **ssps555** | F: TTGCCTTTACCCTTTTGGTG | 255 | 13 | TA | scaffold99 |
|  | R: GGCTGAGATTTGGTCTGCAT |  |  |  |  |
| **ssps558** | F: TGGCCAGTTAGCTTCATTCA | 259 | 17 | TAA | scaffold92 |
|  | R: TCCCGTTCAGCTGGATTTAC |  |  |  |  |
| **ssps559** | F: TTCAATCCCAACTTCCATCC | 146 | 11 | AC | scaffold92 |
|  | R: CTGGGTTTTGTTCCGATGAG |  |  |  |  |
| **ssps560** | F: GGAGGGTAATGCACCCCTAT | 168 | 20 | TA | scaffold98 |
|  | R: GGAAGAAGTCTGGCTCGAAA |  |  |  |  |
| **ssps561** | F: GGTTCGATTCTTCGATCCAT | 250 | 12 | AT | scaffold98 |
|  | R: AGGGATTACCCAAACCGAAT |  |  |  |  |
| **ssps563** | F: CCGCCTTGTTATTGTGGAGT | 293 | 12 | TA | scaffold95 |
|  | R: ACATCGAAAATCAGCCCCTA |  |  |  |  |
| **ssps572** | F: CGCGGAGTGAGAATTTTACG | 183 | 13 | TA | scaffold102 |
|  | R: AGGGCTTGGACAGGTCTTTT |  |  |  |  |
| **ssps574** | F: CATGTGTTTTGGCTGCTTTC | 234 | 19 | CT | scaffold109 |
|  | R: CAGCAATGTGGATGTCCAAG |  |  |  |  |
| **ssps577** | F: TATTTACCCCTCCGCCTTTT | 239 | 17 | TA | scaffold83 |
|  | R: ATGATTTGGGCTGCATTTCT |  |  |  |  |
| **ssps579** | F: ACGCACATGTTCCACTACCA | 230 | 16 | AT | scaffold96 |
|  | R: AGGACACCATTTCCAACCAA |  |  |  |  |
| **ssps581** | F: GCCATTCCATTCCATTTCAC | 167 | 10 | AC | scaffold105 |
|  | R: TGCTGTGTGCGTGTGTGTAT |  |  |  |  |
| **ssps582** | F: GGCCTAAATCTTTGACAGTGC | 287 | 10 | TA | scaffold103 |
|  | R: AAAACCGAAGCTTTTCTACCC |  |  |  |  |
| **ssps586** | F: CCAGAAGGCCCAGTAAATGA | 269 | 19 | TTA | scaffold103 |
|  | R: ATGGCCAACCACTTTGTTTT |  |  |  |  |
| **ssps587** | F: TTTGGTATGGTTCGGTGGTT | 207 | 20 | TA | scaffold115 |
|  | R: ACATCGACCCCTGTTGACAT |  |  |  |  |
| **ssps588** | F: GTTTCTCAGCCTTCCACTGC | 256 | 16 | GT | scaffold115 |
|  | R: TAGTCGCGCAGTTAGCTTCA |  |  |  |  |
| **ssps589** | F: TCGTGTCATGTGTTCGTGTG | 239 | 10 | TA | scaffold84 |
|  | R: CTCACCCAAATGCTCCTCTC |  |  |  |  |
| **ssps590** | F: CACCGCAGAAGACACGTAAA | 154 | 11 | TC | scaffold84 |
|  | R: CCGACTCTTTGATGCGTGTA |  |  |  |  |
| **ssps592** | F: GACCATTCTTCGACGGTTTC | 211 | 11 | TAT | scaffold116 |
|  | R: CAAGAGCACAACACCCACAG |  |  |  |  |
| **ssps593** | F: CATTCGCTCCATTGACTTTG | 296 | 12 | CT | scaffold119 |
|  | R: TTTGGCGGAGGAGTAGATTG |  |  |  |  |
| **ssps594** | F: CCAAATGGGACGAAAATGAA | 242 | 10 | GA | scaffold119 |
|  | R: CGAGTCACCTCCCCTCTGTA |  |  |  |  |
| **ssps595** | F: CTCCTCCTGGGATTGTTGAG | 262 | 15 | GA | scaffold119 |
|  | R: GGATTGGCTTATTTGGAGCA |  |  |  |  |
| **ssps596** | F: CGGAGGAAAATATCGACACG | 155 | 13 | AT | scaffold119 |
|  | R: TTGGGGAATTGATTTTGAGC |  |  |  |  |
| **ssps597** | F: ATTTGGTGCATTAGGGTTCG | 212 | 10 | AT | scaffold112 |
|  | R: TCCAATTCAAGGATCCAAGG |  |  |  |  |
| **ssps598** | F: TGGTCAGCAGTTGCTCATTT | 262 | 19 | CT | scaffold97 |
|  | R: CAGCCTCTTGTGTGTGTGTG |  |  |  |  |
| **ssps600** | F: TTGATGGTCATTGGATGCTC | 297 | 19 | CA | scaffold97 |
|  | R: AGCCCATTTGATGCAACTTT |  |  |  |  |
| **ssps603** | F: ACTCCATCCGTCCACCATTA | 185 | 11 | AT | scaffold117 |
|  | R: AGGAGTCCCGGTTCACTTTT |  |  |  |  |
| **ssps605** | F: GAGTCTGAGAGGCGTCAAGG | 235 | 13 | TAT | scaffold117 |
|  | R: ACCGCGATATCTTTTGTGCT |  |  |  |  |
| **ssps607** | F: CTTTTGCCCTCACATCTTCC | 193 | 10 | GA | scaffold113 |
|  | R: CGAAGCTACCCTCATGAACC |  |  |  |  |
| **ssps608** | F: ACCACCCCATTCATTGCTAA | 125 | 12 | TG | scaffold113 |
|  | R: GGTCACATTCCCAGCAATTC |  |  |  |  |
| **ssps609** | F: TGGTACAACGGGATCAACCT | 284 | 10 | TG | scaffold110 |
|  | R: CCTTTCCCCTTTTCCTTCTG |  |  |  |  |
| **ssps610** | F: TGTGCGTCCATCCAAAATAA | 279 | 10 | TC | scaffold123 |
|  | R: GTACTCCCTTCGTCGCACTC |  |  |  |  |
| **ssps612** | F: CCCACTTCCCTCCATCACTA | 196 | 10 | AC | scaffold4 |
|  | R: TTTGCAATCCGTCGACATAC |  |  |  |  |
| **ssps613** | F: CCGTCATGTTTTCCTCATCC | 243 | 14 | TG | scaffold124 |
|  | R: CCCATGAAAGCCAAAAGCTA |  |  |  |  |
| **ssps616** | F: CAAAATGTGGCTTCGAGTGA | 207 | 15 | GA | scaffold125 |
|  | R: CGGCTTTGCAATTGACTTTT |  |  |  |  |
| **ssps619** | F: GTTCCATCTCCAACGTCCAC | 239 | 14 | TA | scaffold137 |
|  | R: GATGCAGTGCCAGATGATGA |  |  |  |  |
| **ssps620** | F: CGTGGGGCTAAGATCAAGAA | 261 | 20 | TA | scaffold135 |
|  | R: ACATGACCACAAACCCGAAT |  |  |  |  |
| **ssps621** | F: CGTGAACCCAGAAAAGTTCC | 210 | 18 | AT | scaffold134 |
|  | R: TAATTCAGAAAGCCGGTTCG |  |  |  |  |
| **ssps623** | F: ATTGCCCGACATTCACATTT | 196 | 12 | TA | scaffold134 |
|  | R: TCACACACCCACACACACAC |  |  |  |  |
| **ssps624** | F: TTGTCCAACCTCAACAACCA | 274 | 16 | AAT | scaffold138 |
|  | R: TCGACGGTTTCAAGAAATGA |  |  |  |  |
| **ssps625** | F: GACATTTTCACCACCCATCC | 189 | 11 | CCA | scaffold143 |
|  | R: ACTCACCGGCAGTAAGCAAT |  |  |  |  |
| **ssps626** | F: AAAATCCTGCAAATCCAACG | 184 | 17 | CA | scaffold143 |
|  | R: GCCCACTGGCTCATAGTGAT |  |  |  |  |
| **ssps628** | F: CGAGGACTGCAGGAGGATAG | 249 | 12 | ATT | scaffold128 |
|  | R: GCTTTGTGCACGAGAGAAAA |  |  |  |  |
| **ssps629** | F: TGTGGATGCCCTTAGTGTGA | 252 | 13 | TC | scaffold132 |
|  | R: GAGGACGGATTTATGCGTGT |  |  |  |  |
| **ssps634** | F: ACATCGACCCCTGTTGACAT | 212 | 16 | TA | scaffold131 |
|  | R: GAATGGGATCTAACGGCTCA |  |  |  |  |
| **ssps636** | F: TTGAGTCCGGAATGTTGTGA | 262 | 17 | TA | scaffold141 |
|  | R: AGATCGTGGCCATTCATTTC |  |  |  |  |
| **ssps637** | F: ATTGGATGGGACACCACACT | 197 | 10 | GT | scaffold154 |
|  | R: ACAGGGCCTTTCTCCTTCAT |  |  |  |  |
| **ssps638** | F: AGTGCAACTGTGCAAGGGTA | 268 | 11 | AC | scaffold154 |
|  | R: CGTCAAATGTCCTGCTTCAC |  |  |  |  |
| **ssps640** | F: TTTCACCTCTGCCGAAAATC | 146 | 10 | TAGA | scaffold157 |
|  | R: TCTCTCTCGGTTTTGGTGGT |  |  |  |  |
| **ssps644** | F: ATAGCCCAAACCCAAATTCA | 138 | 20 | TC | scaffold118 |
|  | R: CCATTTTGCATCCGTTTTCT |  |  |  |  |
| **ssps647** | F: CCCCATACCCAAACACAAAC | 264 | 11 | CA | scaffold155 |
|  | R: ACCAACTACGGCAAAGGATG |  |  |  |  |
| **ssps648** | F: CAACGCTTGGACGGTAAAAT | 265 | 19 | GA | scaffold175 |
|  | R: CGACGAAGAAGGAGAGGTTG |  |  |  |  |
| **ssps649** | F: CAATTGCGTTTGCAGATGAC | 145 | 10 | TA | scaffold175 |
|  | R: CATGTGATGGGCTGAAAGAA |  |  |  |  |
| **ssps650** | F: GGCTTCAAAGCATCATCACA | 210 | 14 | AT | scaffold171 |
|  | R: GCCGTAGTGTCCAGGATTTC |  |  |  |  |
| **ssps651** | F: ATTGCGGATGCTCTAATGGT | 177 | 11 | GT | scaffold819 |
|  | R: TGGCAATTCGATTGAGAAGA |  |  |  |  |
| **ssps652** | F: CAACCCACTGTCAGGAGTCA | 269 | 10 | TCC | scaffold172 |
|  | R: AGCGTCCTATTCCTTGCTCA |  |  |  |  |
| **ssps653** | F: GCAGGGGAAATGGGTTTATT | 290 | 12 | AG | scaffold146 |
|  | R: CTGCCCTGTTTTCTTTGAGC |  |  |  |  |
| **ssps654** | F: CCATGCGTAATGCTTTTCCT | 206 | 17 | CT | scaffold168 |
|  | R: TGGGTTGACAGTCCATTCCT |  |  |  |  |
| **ssps658** | F: TTTGGAATTGGATTGGGCTA | 276 | 13 | AC | scaffold133 |
|  | R: AGACAGCACCAAGGAGGAAA |  |  |  |  |
| **ssps663** | F: GCCCAATTAGCTTCCAAAGA | 165 | 15 | TA | scaffold190 |
|  | R: AAAGTGCCCACAGTCACAAA |  |  |  |  |
| **ssps667** | F: AGAAGCCGTTGCACATTCTT | 192 | 17 | TC | scaffold198 |
|  | R: TCGAGGCCATTAAGCACTCT |  |  |  |  |
| **ssps669** | F: TCGTGGGGCTGAGATTTAGT | 192 | 11 | ATAC | scaffold204 |
|  | R: CTCCAACAGCCAGTTCCTTT |  |  |  |  |
| **ssps672** | F: TCCTCGTTGTTGCGCTTAAT | 235 | 14 | AT | scaffold216 |
|  | R: GTTAAGGAGCTCGGATGCAG |  |  |  |  |
| **ssps673** | F: ATTGGCCCACCAATTACAAA | 212 | 18 | TG | scaffold219 |
|  | R: AGTAGGCTGAAACCCAATCG |  |  |  |  |
| **ssps675** | F: AAGAAGACGGACACCACACC | 299 | 12 | CT | scaffold220 |
|  | R: GCTTCTGAGAGCAGGCAAAG |  |  |  |  |
| **ssps676** | F: ACACTCTCGACCCGTACGTT | 289 | 11 | GA | scaffold220 |
|  | R: CGGCCTCAATCCACTTTTAC |  |  |  |  |
| **ssps677** | F: TTTCCTTGCCGGACTAACTG | 190 | 14 | CT | scaffold213 |
|  | R: ATTCATTGCCTCCCACACTC |  |  |  |  |
| **ssps681** | F: GCCACCTGCAAATAGGAGAA | 245 | 14 | GT | scaffold562 |
|  | R: ACCTCCATACAAGCCTCACG |  |  |  |  |
| **ssps683** | F: GCCATCAATTGGAAGCTGTT | 298 | 19 | GT | scaffold228 |
|  | R: GCAGCTGACTCACCAACCTT |  |  |  |  |
| **ssps685** | F: CCGATAAACTAACCGGTCCA | 259 | 17 | TTA | scaffold224 |
|  | R: CACGAATTCTGCCTTCATCA |  |  |  |  |
| **ssps686** | F: CGAACGTCTGGGCATATTTT | 267 | 13 | AT | scaffold230 |
|  | R: GTGGGGCTGAGATCAAGAAG |  |  |  |  |
| **ssps689** | F: AATAAATGTTGCCCGTGCTC | 260 | 18 | TC | scaffold226 |
|  | R: CGATTCCAACAACAGGTTCA |  |  |  |  |
| **ssps695** | F: CCCTCTCTCCCTCTCCTCTC | 188 | 10 | GA | scaffold249 |
|  | R: ATCGACTTCCGTCACCACTC |  |  |  |  |
| **ssps696** | F: CCCTTGAAAAGCCTATGTGG | 282 | 10 | TC | scaffold249 |
|  | R: GGAATTAGCAGTTGCGGAGT |  |  |  |  |
| **ssps697** | F: CTTGCACGCACTGAGACTTC | 281 | 12 | AG | scaffold249 |
|  | R: CGTAGGGAGGCACTTGAGAC |  |  |  |  |
| **ssps700-1** | F: CAAACACCGAAATCTCAGCA | 172 | 14 | GA | scaffold254 |
|  | R: AACAAATCTGCCGCATTCAT |  |  |  |  |
| **ssps700-2** | F: CAAACACCGAAATCTCAGCA | 172 | 14 | GA | scaffold254 |
|  | R: AACAAATCTGCCGCATTCAT |  |  |  |  |
| **ssps701** | F: CCATCAACTTATTCCGCTCA | 163 | 12 | AT | scaffold257 |
|  | R: TCCTTCCGTCCCAATTTAAG |  |  |  |  |
| **ssps703** | F: CCATGTCAACAATCCGACAC | 271 | 13 | TA | scaffold273 |
|  | R: GGCATTTTGGCTTCTTTAGC |  |  |  |  |
| **ssps705** | F: TTTCGGCCCATCACTAAAAC | 253 | 11 | GA | scaffold278 |
|  | R: TATTCGGGAACGACGAAATC |  |  |  |  |
| **ssps708** | F: GAGATTAACGGCGGATTTGA | 287 | 10 | TC | scaffold275 |
|  | R: CTGAAATGGCCCAATGAAGT |  |  |  |  |
| **ssps710** | F: TTGAAATGGCCTGCTCAATA | 299 | 10 | ATT | scaffold261 |
|  | R: GCTCCTTCGGATTGATGAAA |  |  |  |  |
| **ssps711** | F: CCGACGTGAACATACACCAC | 299 | 10 | GCT | scaffold281 |
|  | R: TTATGCAGCAGCAGGTTTTG |  |  |  |  |
| **ssps713** | F: ATCAAAACATGAGGGCTTGC | 168 | 15 | CT | scaffold277 |
|  | R: GGAAATAATGCAGCCAGAGC |  |  |  |  |
| **ssps721** | F: CCCAATGTCGAAACACACAC | 207 | 12 | TC | scaffold327 |
|  | R: AGGTGGCAGTGTTCAGTCCT |  |  |  |  |
| **ssps722** | F: GCATCTGTTCCTGCACTCAA | 161 | 13 | AG | scaffold347 |
|  | R: TCCCTTGTGCAGTTGTTTCA |  |  |  |  |
| **ssps724** | F: CAAACACACACACGCAGTGA | 203 | 14 | AC | scaffold340 |
|  | R: GTTGTGCGCGACTCTGAATA |  |  |  |  |
| **ssps726** | F: CCAGGTGAGGCATTCTCATT | 120 | 10 | TTTG | scaffold350 |
|  | R: GAAACGCAAACAGTGAAGCA |  |  |  |  |
| **ssps727** | F: GCACATGCCACTTTGTTCAG | 249 | 12 | AC | scaffold338 |
|  | R: AGCCATAAAAGCACGCCTAC |  |  |  |  |
| **ssps728** | F: TCTCGATCCGACAAAATTCC | 143 | 12 | CA | scaffold367 |
|  | R: GTCGATGGAGGATTGGAAAA |  |  |  |  |
| **ssps730** | F: CTCCTTTGCCTCATTCTTCG | 235 | 12 | AGA | scaffold378 |
|  | R: GCTTCTCGCACCAAATCTTC |  |  |  |  |
| **ssps732** | F: GTAGCGGTTTCCAGCCATTA | 251 | 12 | TA | scaffold398 |
|  | R: CCACGAAGGACATTTTGGAC |  |  |  |  |
| **ssps734** | F: ACAAACAGGGGATTCCACAC | 247 | 11 | TC | scaffold468 |
|  | R: GTCGAACGAATGGCGTTAAT |  |  |  |  |
| **ssps736** | F: ACCAACTACGGCAAAGGATG | 278 | 10 | TG | scaffold531 |
|  | R: CCCCATACCCAAACACAAAC |  |  |  |  |
| **ssps737** | F: ATGCGATGTGTGTGTGTGTG | 249 | 16 | GA | scaffold531 |
|  | R: AGCTAGCCGTGAAAGCAAAA |  |  |  |  |
| **ssps741** | F: CAGCCATAGCCTAGCCACAT | 287 | 10 | CGC | scaffold4 |
|  | R: TGCTTCAAACATTGCTCCTG |  |  |  |  |
| **ssps742** | F: ATTTGGGCGGTTAGATCCTT | 203 | 17 | TA | scaffold4 |
|  | R: AACCGTCGGATGACAAAATC |  |  |  |  |
| **ssps743** | F: CAGCCTATGCACCCTCTCTC | 194 | 14 | ATA | scaffold4 |
|  | R: GCGCCTTTGAGGCTTATTTA |  |  |  |  |
| **ssps757** | F: GGACTTCATGTGCACCGTTA | 171 | 19 | TG | scaffold2 |
|  | R: CAATAGGCAGCCCAGCTTTA |  |  |  |  |
| **ssps758** | F: GGTGCTGATTTTGTAGGTAGGG | 214 | 10 | TA | scaffold1180 |
|  | R: CACTCCGTTGACATCAGTTTGT |  |  |  |  |
| **ssps762** | F: CGTGTTCCCCTCTCTGTGTT | 127 | 16 | GA | scaffold2 |
|  | R: CGTGACCACAACAAATCCAC |  |  |  |  |
| **ssps766** | F: GCTGCTGCTGCTATTTCCTC | 126 | 12 | TC | scaffold2 |
|  | R: GCCTAAATTCAAAGCGCAAC |  |  |  |  |
| **ssps767** | F: TCGTGTCGTCCTAAATGTCCT | 185 | 16 | ATA | scaffold2 |
|  | R: CGAATAGCCCGCTAAATTCAT |  |  |  |  |
| **ssps768** | F: ATTATGGGCAGGCTAGGACT | 228 | 11 | AC | scaffold2 |
|  | R: GCCTCCAAGGCCTATTTAAG |  |  |  |  |

**Table S4.** Statistics calculated for 271 polymorphic SSR markers using eight *S. splendens* cultivar data sets.

| **Locus** | ***N*** | ***Na*** | ***Ne*** | ***I*** | ***Ho*** | ***He*** | ***uHe*** | ***F*** | ***H*** | ***PIC*** | ***E*** | ***H.av*** | ***MI*** | ***D*** |
| --- | --- | --- | --- | --- | --- | --- | --- | --- | --- | --- | --- | --- | --- | --- |
| **ssps195** | 7 | 5 | 3.769 | 1.475 | 0.000 | 0.735 | 0.791 | 1.000 | 0.464 | 0.572 | 1.000 | 0.464 | 0.464 | 0.507 |
| **ssps196** | 7 | 2 | 2.000 | 0.693 | 0.143 | 0.500 | 0.538 | 0.714 | 0.594 | 0.536 | 1.000 | 0.594 | 0.594 | 0.679 |
| **ssps197** | 8 | 4 | 2.612 | 1.157 | 0.125 | 0.617 | 0.658 | 0.797 | 0.404 | 0.536 | 1.000 | 0.404 | 0.404 | 0.927 |
| **ssps198** | 6 | 2 | 1.800 | 0.637 | 0.000 | 0.444 | 0.485 | 1.000 | 0.656 | 0.543 | 1.000 | 0.656 | 0.656 | 0.714 |
| **ssps201** | 8 | 4 | 2.844 | 1.163 | 0.125 | 0.648 | 0.692 | 0.807 | 0.404 | 0.536 | 1.000 | 0.404 | 0.404 | 0.927 |
| **ssps203** | 8 | 2 | 1.280 | 0.377 | 0.000 | 0.219 | 0.233 | 1.000 | 0.500 | 0.493 | 1.000 | 0.500 | 0.500 | 0.767 |
| **ssps204** | 8 | 2 | 1.280 | 0.377 | 0.000 | 0.219 | 0.233 | 1.000 | 0.500 | 0.493 | 1.000 | 0.500 | 0.500 | 0.767 |
| **ssps205** | 7 | 4 | 2.970 | 1.197 | 0.286 | 0.663 | 0.714 | 0.569 | 0.553 | 0.549 | 1.000 | 0.553 | 0.553 | 0.607 |
| **ssps206** | 8 | 3 | 2.327 | 0.947 | 0.875 | 0.570 | 0.608 | -0.534 | 0.469 | 0.508 | 1.000 | 0.469 | 0.469 | 0.620 |
| **ssps207** | 8 | 3 | 2.723 | 1.043 | 0.625 | 0.633 | 0.675 | 0.012 | 0.497 | 0.495 | 1.000 | 0.497 | 0.497 | 0.717 |
| **ssps208** | 6 | 5 | 4.500 | 1.561 | 0.000 | 0.778 | 0.848 | 1.000 | 0.569 | 0.553 | 1.000 | 0.569 | 0.569 | 0.643 |
| **ssps209** | 8 | 2 | 1.600 | 0.562 | 0.000 | 0.375 | 0.400 | 1.000 | 0.531 | 0.555 | 1.000 | 0.531 | 0.531 | 0.607 |
| **ssps210** | 8 | 4 | 3.657 | 1.333 | 0.875 | 0.727 | 0.775 | -0.204 | 0.500 | 0.493 | 1.000 | 0.500 | 0.500 | 0.758 |
| **ssps211** | 2 | 3 | 2.667 | 1.040 | 1.000 | 0.625 | 0.833 | -0.600 | 0.508 | 0.538 | 1.000 | 0.508 | 0.508 | 0.509 |
| **ssps212** | 8 | 4 | 3.200 | 1.255 | 0.750 | 0.688 | 0.733 | -0.091 | 0.549 | 0.521 | 1.000 | 0.549 | 0.549 | 0.509 |
| **ssps213** | 8 | 2 | 1.280 | 0.377 | 0.250 | 0.219 | 0.233 | -0.143 | 0.492 | 0.497 | 1.000 | 0.492 | 0.492 | 0.825 |
| **ssps214** | 6 | 3 | 2.000 | 0.868 | 0.000 | 0.500 | 0.545 | 1.000 | 0.542 | 0.536 | 1.000 | 0.542 | 0.542 | 0.381 |
| **ssps222** | 8 | 8 | 5.818 | 1.923 | 0.375 | 0.828 | 0.883 | 0.547 | 0.380 | 0.579 | 1.000 | 0.380 | 0.380 | 0.397 |
| **ssps224** | 8 | 4 | 2.844 | 1.163 | 1.000 | 0.648 | 0.692 | -0.542 | 0.430 | 0.526 | 1.000 | 0.430 | 0.430 | 0.909 |
| **ssps237** | 8 | 5 | 2.327 | 1.160 | 0.250 | 0.570 | 0.608 | 0.562 | 0.480 | 0.503 | 1.000 | 0.480 | 0.480 | 0.846 |
| **ssps238** | 7 | 2 | 1.508 | 0.520 | 0.143 | 0.337 | 0.363 | 0.576 | 0.219 | 0.594 | 1.000 | 0.219 | 0.219 | 0.992 |
| **ssps240** | 8 | 3 | 2.415 | 0.984 | 0.625 | 0.586 | 0.625 | -0.067 | 0.573 | 0.524 | 1.000 | 0.573 | 0.573 | 0.536 |
| **ssps243** | 8 | 2 | 1.600 | 0.562 | 0.000 | 0.375 | 0.400 | 1.000 | 0.469 | 0.508 | 1.000 | 0.469 | 0.469 | 0.625 |
| **ssps244** | 8 | 4 | 2.723 | 1.143 | 1.000 | 0.633 | 0.675 | -0.580 | 0.492 | 0.497 | 1.000 | 0.492 | 0.492 | 0.692 |
| **ssps250** | 6 | 3 | 2.000 | 0.868 | 0.000 | 0.500 | 0.545 | 1.000 | 0.517 | 0.545 | 1.000 | 0.517 | 0.517 | 0.464 |
| **ssps251** | 8 | 3 | 2.327 | 0.947 | 0.125 | 0.570 | 0.608 | 0.781 | 0.611 | 0.542 | 1.000 | 0.611 | 0.611 | 0.619 |
| **ssps254** | 8 | 3 | 1.471 | 0.602 | 0.125 | 0.320 | 0.342 | 0.610 | 0.413 | 0.533 | 1.000 | 0.413 | 0.413 | 0.924 |
| **ssps255** | 8 | 2 | 1.438 | 0.483 | 0.125 | 0.305 | 0.325 | 0.590 | 0.500 | 0.493 | 1.000 | 0.500 | 0.500 | 0.767 |
| **ssps258** | 8 | 5 | 4.571 | 1.560 | 0.000 | 0.781 | 0.833 | 1.000 | 0.455 | 0.515 | 1.000 | 0.455 | 0.455 | 0.883 |
| **ssps270** | 8 | 5 | 3.657 | 1.455 | 0.125 | 0.727 | 0.775 | 0.828 | 0.349 | 0.557 | 1.000 | 0.349 | 0.349 | 0.954 |
| **ssps272** | 8 | 2 | 1.600 | 0.562 | 0.000 | 0.375 | 0.400 | 1.000 | 0.305 | 0.572 | 1.000 | 0.305 | 0.305 | 0.975 |
| **ssps274** | 8 | 2 | 1.969 | 0.685 | 0.125 | 0.492 | 0.525 | 0.746 | 0.500 | 0.493 | 1.000 | 0.500 | 0.500 | 0.767 |
| **ssps279** | 8 | 2 | 1.438 | 0.483 | 0.375 | 0.305 | 0.325 | -0.231 | 0.492 | 0.497 | 1.000 | 0.492 | 0.492 | 0.700 |
| **ssps284** | 7 | 6 | 5.444 | 1.748 | 0.000 | 0.816 | 0.879 | 1.000 | 0.542 | 0.536 | 1.000 | 0.542 | 0.542 | 0.423 |
| **ssps285** | 8 | 3 | 2.133 | 0.900 | 0.000 | 0.531 | 0.567 | 1.000 | 0.403 | 0.579 | 1.000 | 0.403 | 0.403 | 0.452 |
| **ssps286** | 7 | 3 | 2.333 | 0.956 | 0.000 | 0.571 | 0.615 | 1.000 | 0.497 | 0.529 | 1.000 | 0.497 | 0.497 | 0.464 |
| **ssps287** | 6 | 3 | 2.000 | 0.868 | 0.000 | 0.500 | 0.545 | 1.000 | 0.625 | 0.444 | 1.000 | 0.625 | 0.625 | 0.643 |
| **ssps288** | 7 | 2 | 1.690 | 0.598 | 0.000 | 0.408 | 0.440 | 1.000 | 0.602 | 0.429 | 1.000 | 0.602 | 0.602 | 0.607 |
| **ssps290** | 6 | 3 | 1.674 | 0.721 | 0.167 | 0.403 | 0.439 | 0.586 | 0.580 | 0.436 | 1.000 | 0.580 | 0.580 | 0.464 |
| **ssps291** | 7 | 3 | 2.579 | 1.004 | 0.000 | 0.612 | 0.659 | 1.000 | 0.559 | 0.443 | 1.000 | 0.559 | 0.559 | 0.607 |
| **ssps292** | 8 | 3 | 2.133 | 0.900 | 0.000 | 0.531 | 0.567 | 1.000 | 0.444 | 0.416 | 1.000 | 0.444 | 0.444 | 0.899 |
| **ssps293** | 8 | 2 | 1.600 | 0.562 | 0.000 | 0.375 | 0.400 | 1.000 | 0.500 | 0.389 | 1.000 | 0.500 | 0.500 | 0.767 |
| **ssps294** | 8 | 2 | 1.600 | 0.562 | 0.250 | 0.375 | 0.400 | 0.333 | 0.469 | 0.404 | 1.000 | 0.469 | 0.469 | 0.625 |
| **ssps296** | 8 | 3 | 1.684 | 0.736 | 0.000 | 0.406 | 0.433 | 1.000 | 0.444 | 0.416 | 1.000 | 0.444 | 0.444 | 0.899 |
| **ssps298** | 8 | 3 | 2.612 | 1.024 | 0.375 | 0.617 | 0.658 | 0.392 | 0.497 | 0.391 | 1.000 | 0.497 | 0.497 | 0.801 |
| **ssps299** | 8 | 2 | 1.600 | 0.562 | 0.000 | 0.375 | 0.400 | 1.000 | 0.500 | 0.389 | 1.000 | 0.500 | 0.500 | 0.767 |
| **ssps301** | 8 | 2 | 1.280 | 0.377 | 0.000 | 0.219 | 0.233 | 1.000 | 0.500 | 0.389 | 1.000 | 0.500 | 0.500 | 0.767 |
| **ssps302** | 8 | 5 | 3.122 | 1.354 | 0.125 | 0.680 | 0.725 | 0.816 | 0.349 | 0.454 | 1.000 | 0.349 | 0.349 | 0.954 |
| **ssps303** | 8 | 3 | 2.462 | 0.974 | 0.750 | 0.594 | 0.633 | -0.263 | 0.486 | 0.396 | 1.000 | 0.486 | 0.486 | 0.670 |
| **ssps304** | 7 | 2 | 1.690 | 0.598 | 0.000 | 0.408 | 0.440 | 1.000 | 0.602 | 0.429 | 1.000 | 0.602 | 0.602 | 0.607 |
| **ssps305** | 7 | 4 | 2.800 | 1.171 | 0.143 | 0.643 | 0.692 | 0.778 | 0.531 | 0.451 | 1.000 | 0.531 | 0.531 | 0.554 |
| **ssps308** | 8 | 7 | 6.095 | 1.873 | 0.250 | 0.836 | 0.892 | 0.701 | 0.293 | 0.471 | 1.000 | 0.293 | 0.293 | 0.971 |
| **ssps309** | 8 | 4 | 3.200 | 1.255 | 0.000 | 0.688 | 0.733 | 1.000 | 0.375 | 0.444 | 1.000 | 0.375 | 0.375 | 0.944 |
| **ssps314** | 8 | 2 | 1.969 | 0.685 | 0.125 | 0.492 | 0.525 | 0.746 | 0.492 | 0.393 | 1.000 | 0.492 | 0.492 | 0.700 |
| **ssps315** | 7 | 5 | 3.379 | 1.376 | 0.143 | 0.704 | 0.758 | 0.797 | 0.489 | 0.462 | 1.000 | 0.489 | 0.489 | 0.536 |
| **ssps318** | 8 | 2 | 1.280 | 0.377 | 0.000 | 0.219 | 0.233 | 1.000 | 0.500 | 0.389 | 1.000 | 0.500 | 0.500 | 0.767 |
| **ssps321** | 8 | 2 | 1.969 | 0.685 | 0.125 | 0.492 | 0.525 | 0.746 | 0.492 | 0.393 | 1.000 | 0.492 | 0.492 | 0.700 |
| **ssps322** | 8 | 3 | 1.910 | 0.831 | 0.125 | 0.477 | 0.508 | 0.738 | 0.469 | 0.404 | 1.000 | 0.469 | 0.469 | 0.870 |
| **ssps324** | 7 | 3 | 1.815 | 0.796 | 0.000 | 0.449 | 0.484 | 1.000 | 0.559 | 0.443 | 1.000 | 0.559 | 0.559 | 0.512 |
| **ssps325-1** | 7 | 2 | 1.324 | 0.410 | 0.000 | 0.245 | 0.264 | 1.000 | 0.602 | 0.429 | 1.000 | 0.602 | 0.602 | 0.464 |
| **ssps325-2** | 6 | 2 | 1.385 | 0.451 | 0.000 | 0.278 | 0.303 | 1.000 | 0.656 | 0.440 | 1.000 | 0.656 | 0.656 | 0.607 |
| **ssps328** | 8 | 2 | 2.000 | 0.693 | 0.000 | 0.500 | 0.533 | 1.000 | 0.500 | 0.389 | 1.000 | 0.500 | 0.500 | 0.767 |
| **ssps329** | 8 | 5 | 2.327 | 1.160 | 0.125 | 0.570 | 0.608 | 0.781 | 0.349 | 0.454 | 1.000 | 0.349 | 0.349 | 0.954 |
| **ssps330** | 5 | 4 | 3.571 | 1.332 | 0.000 | 0.720 | 0.800 | 1.000 | 0.615 | 0.435 | 1.000 | 0.615 | 0.615 | 0.696 |
| **ssps331** | 8 | 2 | 1.280 | 0.377 | 0.000 | 0.219 | 0.233 | 1.000 | 0.500 | 0.389 | 1.000 | 0.500 | 0.500 | 0.767 |
| **ssps334** | 8 | 3 | 2.667 | 1.040 | 0.000 | 0.625 | 0.667 | 1.000 | 0.444 | 0.416 | 1.000 | 0.444 | 0.444 | 0.899 |
| **ssps336** | 8 | 4 | 2.286 | 1.074 | 0.000 | 0.563 | 0.600 | 1.000 | 0.375 | 0.444 | 1.000 | 0.375 | 0.375 | 0.944 |
| **ssps337** | 8 | 2 | 1.280 | 0.377 | 0.000 | 0.219 | 0.233 | 1.000 | 0.500 | 0.389 | 1.000 | 0.500 | 0.500 | 0.767 |
| **ssps338** | 8 | 3 | 1.684 | 0.736 | 0.000 | 0.406 | 0.433 | 1.000 | 0.444 | 0.416 | 1.000 | 0.444 | 0.444 | 0.899 |
| **ssps339** | 8 | 5 | 4.000 | 1.494 | 0.000 | 0.750 | 0.800 | 1.000 | 0.320 | 0.463 | 1.000 | 0.320 | 0.320 | 0.964 |
| **ssps343** | 8 | 2 | 1.600 | 0.562 | 0.250 | 0.375 | 0.400 | 0.333 | 0.469 | 0.404 | 1.000 | 0.469 | 0.469 | 0.625 |
| **ssps344** | 8 | 3 | 1.471 | 0.602 | 0.125 | 0.320 | 0.342 | 0.610 | 0.469 | 0.417 | 1.000 | 0.469 | 0.469 | 0.870 |
| **ssps347** | 8 | 3 | 2.909 | 1.082 | 0.000 | 0.656 | 0.700 | 1.000 | 0.444 | 0.428 | 1.000 | 0.444 | 0.444 | 0.899 |
| **ssps348** | 8 | 5 | 2.723 | 1.277 | 0.250 | 0.633 | 0.675 | 0.605 | 0.375 | 0.456 | 1.000 | 0.375 | 0.375 | 0.942 |
| **ssps357** | 8 | 2 | 2.000 | 0.693 | 0.250 | 0.500 | 0.533 | 0.500 | 0.469 | 0.417 | 1.000 | 0.469 | 0.469 | 0.625 |
| **ssps358** | 6 | 5 | 4.500 | 1.561 | 0.000 | 0.778 | 0.848 | 1.000 | 0.555 | 0.463 | 1.000 | 0.555 | 0.555 | 0.629 |
| **ssps359** | 6 | 2 | 1.800 | 0.637 | 0.000 | 0.444 | 0.485 | 1.000 | 0.656 | 0.452 | 1.000 | 0.656 | 0.656 | 0.714 |
| **ssps361** | 7 | 3 | 2.333 | 0.956 | 0.000 | 0.571 | 0.615 | 1.000 | 0.559 | 0.455 | 1.000 | 0.559 | 0.559 | 0.583 |
| **ssps364** | 8 | 2 | 1.280 | 0.377 | 0.250 | 0.219 | 0.233 | -0.143 | 0.469 | 0.417 | 1.000 | 0.469 | 0.469 | 0.625 |
| **ssps368** | 7 | 2 | 1.324 | 0.410 | 0.000 | 0.245 | 0.264 | 1.000 | 0.602 | 0.441 | 1.000 | 0.602 | 0.602 | 0.464 |
| **ssps370** | 8 | 3 | 1.684 | 0.736 | 0.250 | 0.406 | 0.433 | 0.385 | 0.486 | 0.408 | 1.000 | 0.486 | 0.486 | 0.837 |
| **ssps373** | 8 | 3 | 2.133 | 0.900 | 0.000 | 0.531 | 0.567 | 1.000 | 0.444 | 0.428 | 1.000 | 0.444 | 0.444 | 0.899 |
| **ssps374** | 7 | 2 | 1.690 | 0.598 | 0.000 | 0.408 | 0.440 | 1.000 | 0.602 | 0.441 | 1.000 | 0.602 | 0.602 | 0.607 |
| **ssps377** | 8 | 2 | 1.280 | 0.377 | 0.000 | 0.219 | 0.233 | 1.000 | 0.500 | 0.402 | 1.000 | 0.500 | 0.500 | 0.767 |
| **ssps378-1** | 7 | 3 | 1.556 | 0.656 | 0.143 | 0.357 | 0.385 | 0.600 | 0.580 | 0.449 | 1.000 | 0.580 | 0.580 | 0.464 |
| **ssps378-2** | 7 | 5 | 3.769 | 1.475 | 0.000 | 0.735 | 0.791 | 1.000 | 0.464 | 0.480 | 1.000 | 0.464 | 0.464 | 0.507 |
| **ssps380** | 8 | 3 | 2.844 | 1.072 | 0.125 | 0.648 | 0.692 | 0.807 | 0.469 | 0.417 | 1.000 | 0.469 | 0.469 | 0.870 |
| **ssps381** | 7 | 3 | 1.815 | 0.796 | 0.000 | 0.449 | 0.484 | 1.000 | 0.559 | 0.455 | 1.000 | 0.559 | 0.559 | 0.512 |
| **ssps383** | 8 | 2 | 1.969 | 0.685 | 0.125 | 0.492 | 0.525 | 0.746 | 0.492 | 0.405 | 1.000 | 0.492 | 0.492 | 0.700 |
| **ssps385** | 8 | 2 | 1.969 | 0.685 | 0.375 | 0.492 | 0.525 | 0.238 | 0.430 | 0.434 | 1.000 | 0.430 | 0.430 | 0.542 |
| **ssps387** | 8 | 3 | 2.462 | 0.974 | 0.500 | 0.594 | 0.633 | 0.158 | 0.500 | 0.402 | 1.000 | 0.500 | 0.500 | 0.761 |
| **ssps388** | 8 | 2 | 1.280 | 0.377 | 0.250 | 0.219 | 0.233 | -0.143 | 0.469 | 0.417 | 1.000 | 0.469 | 0.469 | 0.625 |
| **ssps389** | 8 | 2 | 1.882 | 0.662 | 0.000 | 0.469 | 0.500 | 1.000 | 0.500 | 0.402 | 1.000 | 0.500 | 0.500 | 0.767 |
| **ssps390** | 8 | 5 | 3.556 | 1.392 | 0.250 | 0.719 | 0.767 | 0.652 | 0.375 | 0.456 | 1.000 | 0.375 | 0.375 | 0.942 |
| **ssps391** | 7 | 5 | 4.455 | 1.550 | 0.000 | 0.776 | 0.835 | 1.000 | 0.464 | 0.480 | 1.000 | 0.464 | 0.464 | 0.521 |
| **ssps393** | 8 | 3 | 1.471 | 0.602 | 0.125 | 0.320 | 0.342 | 0.610 | 0.469 | 0.417 | 1.000 | 0.469 | 0.469 | 0.870 |
| **ssps394** | 8 | 2 | 1.280 | 0.377 | 0.000 | 0.219 | 0.233 | 1.000 | 0.500 | 0.402 | 1.000 | 0.500 | 0.500 | 0.767 |
| **ssps395** | 8 | 4 | 2.510 | 1.103 | 0.125 | 0.602 | 0.642 | 0.792 | 0.404 | 0.445 | 1.000 | 0.404 | 0.404 | 0.927 |
| **ssps396** | 8 | 3 | 2.133 | 0.900 | 0.000 | 0.531 | 0.567 | 1.000 | 0.444 | 0.428 | 1.000 | 0.444 | 0.444 | 0.899 |
| **ssps399** | 8 | 2 | 1.280 | 0.377 | 0.000 | 0.219 | 0.233 | 1.000 | 0.500 | 0.402 | 1.000 | 0.500 | 0.500 | 0.767 |
| **ssps401** | 8 | 2 | 1.133 | 0.234 | 0.125 | 0.117 | 0.125 | -0.067 | 0.492 | 0.405 | 1.000 | 0.492 | 0.492 | 0.700 |
| **ssps404-1** | 8 | 2 | 1.600 | 0.562 | 0.000 | 0.375 | 0.400 | 1.000 | 0.500 | 0.402 | 1.000 | 0.500 | 0.500 | 0.767 |
| **ssps404-2** | 8 | 2 | 1.280 | 0.377 | 0.000 | 0.219 | 0.233 | 1.000 | 0.500 | 0.402 | 1.000 | 0.500 | 0.500 | 0.767 |
| **ssps405** | 8 | 3 | 2.133 | 0.900 | 0.000 | 0.531 | 0.567 | 1.000 | 0.444 | 0.428 | 1.000 | 0.444 | 0.444 | 0.899 |
| **ssps407** | 8 | 3 | 2.169 | 0.865 | 0.625 | 0.539 | 0.575 | -0.159 | 0.497 | 0.403 | 1.000 | 0.497 | 0.497 | 0.717 |
| **ssps410** | 6 | 2 | 1.800 | 0.637 | 0.000 | 0.444 | 0.485 | 1.000 | 0.656 | 0.452 | 1.000 | 0.656 | 0.656 | 0.714 |
| **ssps411** | 8 | 2 | 1.882 | 0.662 | 0.000 | 0.469 | 0.500 | 1.000 | 0.500 | 0.372 | 1.000 | 0.500 | 0.500 | 0.767 |
| **ssps426** | 7 | 2 | 1.960 | 0.683 | 0.000 | 0.490 | 0.527 | 1.000 | 0.602 | 0.412 | 1.000 | 0.602 | 0.602 | 0.679 |
| **ssps427** | 8 | 4 | 2.169 | 0.987 | 0.250 | 0.539 | 0.575 | 0.536 | 0.430 | 0.405 | 1.000 | 0.430 | 0.430 | 0.909 |
| **ssps428** | 8 | 3 | 2.844 | 1.072 | 0.500 | 0.648 | 0.692 | 0.229 | 0.500 | 0.372 | 1.000 | 0.500 | 0.500 | 0.761 |
| **ssps429** | 8 | 2 | 1.280 | 0.377 | 0.000 | 0.219 | 0.233 | 1.000 | 0.500 | 0.372 | 1.000 | 0.500 | 0.500 | 0.767 |
| **ssps430** | 8 | 4 | 2.612 | 1.157 | 0.125 | 0.617 | 0.658 | 0.797 | 0.404 | 0.416 | 1.000 | 0.404 | 0.404 | 0.927 |
| **ssps433** | 8 | 2 | 1.753 | 0.621 | 0.125 | 0.430 | 0.458 | 0.709 | 0.492 | 0.376 | 1.000 | 0.492 | 0.492 | 0.700 |
| **ssps434** | 8 | 4 | 3.368 | 1.303 | 0.250 | 0.703 | 0.750 | 0.644 | 0.430 | 0.405 | 1.000 | 0.430 | 0.430 | 0.909 |
| **ssps435** | 8 | 4 | 1.707 | 0.822 | 0.375 | 0.414 | 0.442 | 0.094 | 0.451 | 0.396 | 1.000 | 0.451 | 0.451 | 0.889 |
| **ssps441** | 8 | 2 | 1.280 | 0.377 | 0.000 | 0.219 | 0.233 | 1.000 | 0.500 | 0.372 | 1.000 | 0.500 | 0.500 | 0.767 |
| **ssps442** | 7 | 2 | 1.960 | 0.683 | 0.000 | 0.490 | 0.527 | 1.000 | 0.602 | 0.412 | 1.000 | 0.602 | 0.602 | 0.679 |
| **ssps445** | 8 | 5 | 4.000 | 1.494 | 0.000 | 0.750 | 0.800 | 1.000 | 0.320 | 0.446 | 1.000 | 0.320 | 0.320 | 0.964 |
| **ssps446** | 8 | 7 | 5.333 | 1.804 | 0.500 | 0.813 | 0.867 | 0.385 | 0.337 | 0.441 | 1.000 | 0.337 | 0.337 | 0.957 |
| **ssps447** | 8 | 2 | 1.882 | 0.662 | 0.000 | 0.469 | 0.500 | 1.000 | 0.500 | 0.372 | 1.000 | 0.500 | 0.500 | 0.767 |
| **ssps448** | 8 | 2 | 1.882 | 0.662 | 0.250 | 0.469 | 0.500 | 0.467 | 0.469 | 0.387 | 1.000 | 0.469 | 0.469 | 0.625 |
| **ssps449** | 8 | 2 | 1.753 | 0.621 | 0.125 | 0.430 | 0.458 | 0.709 | 0.492 | 0.376 | 1.000 | 0.492 | 0.492 | 0.700 |
| **ssps451** | 8 | 4 | 1.707 | 0.822 | 0.375 | 0.414 | 0.442 | 0.094 | 0.451 | 0.396 | 1.000 | 0.451 | 0.451 | 0.889 |
| **ssps452** | 8 | 4 | 2.510 | 1.061 | 0.500 | 0.602 | 0.642 | 0.169 | 0.500 | 0.372 | 1.000 | 0.500 | 0.500 | 0.761 |
| **ssps453** | 8 | 3 | 2.169 | 0.865 | 0.125 | 0.539 | 0.575 | 0.768 | 0.469 | 0.387 | 1.000 | 0.469 | 0.469 | 0.870 |
| **ssps459** | 8 | 3 | 2.462 | 0.974 | 1.000 | 0.594 | 0.633 | -0.684 | 0.444 | 0.399 | 1.000 | 0.444 | 0.444 | 0.565 |
| **ssps460** | 8 | 4 | 3.048 | 1.212 | 1.000 | 0.672 | 0.717 | -0.488 | 0.500 | 0.372 | 1.000 | 0.500 | 0.500 | 0.758 |
| **ssps462-1** | 7 | 2 | 1.324 | 0.410 | 0.000 | 0.245 | 0.264 | 1.000 | 0.602 | 0.412 | 1.000 | 0.602 | 0.602 | 0.464 |
| **ssps462-2** | 8 | 4 | 3.556 | 1.321 | 0.000 | 0.719 | 0.767 | 1.000 | 0.375 | 0.427 | 1.000 | 0.375 | 0.375 | 0.944 |
| **ssps466** | 8 | 2 | 1.753 | 0.621 | 0.625 | 0.430 | 0.458 | -0.455 | 0.305 | 0.451 | 1.000 | 0.305 | 0.305 | 0.350 |
| **ssps467** | 8 | 2 | 1.600 | 0.562 | 0.000 | 0.375 | 0.400 | 1.000 | 0.500 | 0.372 | 1.000 | 0.500 | 0.500 | 0.767 |
| **ssps469** | 8 | 4 | 3.122 | 1.245 | 0.125 | 0.680 | 0.725 | 0.816 | 0.404 | 0.416 | 1.000 | 0.404 | 0.404 | 0.927 |
| **ssps471** | 8 | 4 | 1.707 | 0.822 | 0.125 | 0.414 | 0.442 | 0.698 | 0.404 | 0.416 | 1.000 | 0.404 | 0.404 | 0.927 |
| **ssps476** | 7 | 2 | 1.324 | 0.410 | 0.000 | 0.245 | 0.264 | 1.000 | 0.602 | 0.412 | 1.000 | 0.602 | 0.602 | 0.464 |
| **ssps488** | 8 | 4 | 1.969 | 0.951 | 0.125 | 0.492 | 0.525 | 0.746 | 0.404 | 0.416 | 1.000 | 0.404 | 0.404 | 0.927 |
| **ssps492-1** | 8 | 2 | 1.600 | 0.562 | 0.000 | 0.375 | 0.400 | 1.000 | 0.500 | 0.372 | 1.000 | 0.500 | 0.500 | 0.767 |
| **ssps492-2** | 7 | 2 | 1.324 | 0.410 | 0.000 | 0.245 | 0.264 | 1.000 | 0.602 | 0.412 | 1.000 | 0.602 | 0.602 | 0.464 |
| **ssps493** | 8 | 2 | 1.969 | 0.685 | 0.625 | 0.492 | 0.525 | -0.270 | 0.305 | 0.451 | 1.000 | 0.305 | 0.305 | 0.350 |
| **ssps495** | 8 | 2 | 1.280 | 0.377 | 0.000 | 0.219 | 0.233 | 1.000 | 0.500 | 0.372 | 1.000 | 0.500 | 0.500 | 0.767 |
| **ssps496** | 8 | 2 | 1.600 | 0.562 | 0.000 | 0.375 | 0.400 | 1.000 | 0.500 | 0.372 | 1.000 | 0.500 | 0.500 | 0.767 |
| **ssps500** | 8 | 2 | 1.133 | 0.234 | 0.125 | 0.117 | 0.125 | -0.067 | 0.492 | 0.376 | 1.000 | 0.492 | 0.492 | 0.700 |
| **ssps501** | 8 | 3 | 2.327 | 0.947 | 0.125 | 0.570 | 0.608 | 0.781 | 0.469 | 0.442 | 1.000 | 0.469 | 0.469 | 0.870 |
| **ssps502** | 8 | 2 | 1.280 | 0.377 | 0.000 | 0.219 | 0.233 | 1.000 | 0.500 | 0.426 | 1.000 | 0.500 | 0.500 | 0.767 |
| **ssps505** | 8 | 2 | 1.280 | 0.377 | 0.000 | 0.219 | 0.233 | 1.000 | 0.500 | 0.426 | 1.000 | 0.500 | 0.500 | 0.767 |
| **ssps507** | 8 | 3 | 2.032 | 0.831 | 0.375 | 0.508 | 0.542 | 0.262 | 0.497 | 0.428 | 1.000 | 0.497 | 0.497 | 0.801 |
| **ssps508** | 8 | 2 | 1.280 | 0.377 | 0.000 | 0.219 | 0.233 | 1.000 | 0.500 | 0.426 | 1.000 | 0.500 | 0.500 | 0.767 |
| **ssps510** | 7 | 4 | 3.379 | 1.291 | 0.286 | 0.704 | 0.758 | 0.594 | 0.553 | 0.482 | 1.000 | 0.553 | 0.553 | 0.607 |
| **ssps511** | 8 | 4 | 2.844 | 1.163 | 0.375 | 0.648 | 0.692 | 0.422 | 0.451 | 0.450 | 1.000 | 0.451 | 0.451 | 0.889 |
| **ssps512** | 8 | 2 | 1.882 | 0.662 | 0.250 | 0.469 | 0.500 | 0.467 | 0.469 | 0.442 | 1.000 | 0.469 | 0.469 | 0.625 |
| **ssps514** | 8 | 2 | 1.280 | 0.377 | 0.000 | 0.219 | 0.233 | 1.000 | 0.500 | 0.426 | 1.000 | 0.500 | 0.500 | 0.767 |
| **ssps517** | 8 | 4 | 2.723 | 1.143 | 0.750 | 0.633 | 0.675 | -0.185 | 0.492 | 0.430 | 1.000 | 0.492 | 0.492 | 0.817 |
| **ssps520** | 8 | 3 | 2.327 | 0.947 | 0.625 | 0.570 | 0.608 | -0.096 | 0.497 | 0.428 | 1.000 | 0.497 | 0.497 | 0.717 |
| **ssps523** | 8 | 4 | 3.556 | 1.321 | 0.000 | 0.719 | 0.767 | 1.000 | 0.375 | 0.481 | 1.000 | 0.375 | 0.375 | 0.944 |
| **ssps530** | 8 | 2 | 1.280 | 0.377 | 0.000 | 0.219 | 0.233 | 1.000 | 0.500 | 0.426 | 1.000 | 0.500 | 0.500 | 0.767 |
| **ssps531** | 7 | 5 | 3.063 | 1.296 | 1.000 | 0.673 | 0.725 | -0.485 | 0.586 | 0.472 | 1.000 | 0.586 | 0.586 | 0.493 |
| **ssps535** | 8 | 2 | 1.280 | 0.377 | 0.000 | 0.219 | 0.233 | 1.000 | 0.500 | 0.426 | 1.000 | 0.500 | 0.500 | 0.767 |
| **ssps537** | 8 | 3 | 2.510 | 0.983 | 0.875 | 0.602 | 0.642 | -0.455 | 0.469 | 0.442 | 1.000 | 0.469 | 0.469 | 0.620 |
| **ssps538** | 6 | 3 | 1.946 | 0.824 | 0.333 | 0.486 | 0.530 | 0.314 | 0.653 | 0.477 | 1.000 | 0.653 | 0.653 | 0.643 |
| **ssps539** | 7 | 2 | 1.508 | 0.520 | 0.143 | 0.337 | 0.363 | 0.576 | 0.594 | 0.469 | 1.000 | 0.594 | 0.594 | 0.536 |
| **ssps543** | 8 | 3 | 2.415 | 0.984 | 0.875 | 0.586 | 0.625 | -0.493 | 0.469 | 0.442 | 1.000 | 0.469 | 0.469 | 0.620 |
| **ssps544** | 8 | 3 | 2.169 | 0.865 | 0.125 | 0.539 | 0.575 | 0.768 | 0.469 | 0.442 | 1.000 | 0.469 | 0.469 | 0.870 |
| **ssps546** | 6 | 4 | 3.429 | 1.286 | 0.167 | 0.708 | 0.773 | 0.765 | 0.607 | 0.483 | 1.000 | 0.607 | 0.607 | 0.688 |
| **ssps547** | 8 | 2 | 1.600 | 0.562 | 0.000 | 0.375 | 0.400 | 1.000 | 0.500 | 0.426 | 1.000 | 0.500 | 0.500 | 0.767 |
| **ssps548** | 8 | 3 | 1.684 | 0.736 | 0.000 | 0.406 | 0.433 | 1.000 | 0.444 | 0.453 | 1.000 | 0.444 | 0.444 | 0.899 |
| **ssps549** | 5 | 3 | 2.381 | 0.943 | 0.200 | 0.580 | 0.644 | 0.655 | 0.656 | 0.477 | 1.000 | 0.656 | 0.656 | 0.726 |
| **ssps553a** | 5 | 4 | 3.571 | 1.332 | 0.000 | 0.720 | 0.800 | 1.000 | 0.615 | 0.472 | 1.000 | 0.615 | 0.615 | 0.696 |
| **ssps553b** | 7 | 3 | 1.342 | 0.509 | 0.143 | 0.255 | 0.275 | 0.440 | 0.580 | 0.474 | 1.000 | 0.580 | 0.580 | 0.464 |
| **ssps554** | 8 | 3 | 2.169 | 0.865 | 0.125 | 0.539 | 0.575 | 0.768 | 0.469 | 0.442 | 1.000 | 0.469 | 0.469 | 0.870 |
| **ssps555** | 8 | 2 | 2.000 | 0.693 | 0.000 | 0.500 | 0.533 | 1.000 | 0.500 | 0.426 | 1.000 | 0.500 | 0.500 | 0.767 |
| **ssps558** | 8 | 2 | 1.600 | 0.562 | 0.000 | 0.375 | 0.400 | 1.000 | 0.500 | 0.426 | 1.000 | 0.500 | 0.500 | 0.767 |
| **ssps559** | 8 | 2 | 1.600 | 0.562 | 0.000 | 0.375 | 0.400 | 1.000 | 0.500 | 0.426 | 1.000 | 0.500 | 0.500 | 0.767 |
| **ssps560** | 8 | 2 | 1.280 | 0.377 | 0.000 | 0.219 | 0.233 | 1.000 | 0.500 | 0.426 | 1.000 | 0.500 | 0.500 | 0.767 |
| **ssps561** | 8 | 3 | 1.684 | 0.736 | 0.000 | 0.406 | 0.433 | 1.000 | 0.444 | 0.453 | 1.000 | 0.444 | 0.444 | 0.899 |
| **ssps563** | 7 | 2 | 1.324 | 0.410 | 0.000 | 0.245 | 0.264 | 1.000 | 0.602 | 0.466 | 1.000 | 0.602 | 0.602 | 0.464 |
| **ssps572** | 8 | 2 | 1.438 | 0.483 | 0.125 | 0.305 | 0.325 | 0.590 | 0.492 | 0.430 | 1.000 | 0.492 | 0.492 | 0.700 |
| **ssps574** | 8 | 4 | 3.282 | 1.251 | 0.375 | 0.695 | 0.742 | 0.461 | 0.451 | 0.450 | 1.000 | 0.451 | 0.451 | 0.889 |
| **ssps577** | 8 | 4 | 2.844 | 1.163 | 0.375 | 0.648 | 0.692 | 0.422 | 0.451 | 0.450 | 1.000 | 0.451 | 0.451 | 0.889 |
| **ssps579** | 6 | 4 | 3.000 | 1.242 | 0.000 | 0.667 | 0.727 | 1.000 | 0.586 | 0.485 | 1.000 | 0.586 | 0.586 | 0.643 |
| **ssps581** | 8 | 2 | 1.600 | 0.562 | 0.250 | 0.375 | 0.400 | 0.333 | 0.469 | 0.442 | 1.000 | 0.469 | 0.469 | 0.625 |
| **ssps582** | 8 | 3 | 1.684 | 0.736 | 0.000 | 0.406 | 0.433 | 1.000 | 0.444 | 0.419 | 1.000 | 0.444 | 0.444 | 0.899 |
| **ssps586** | 6 | 2 | 1.385 | 0.451 | 0.000 | 0.278 | 0.303 | 1.000 | 0.656 | 0.444 | 1.000 | 0.656 | 0.656 | 0.607 |
| **ssps587** | 7 | 2 | 1.324 | 0.410 | 0.000 | 0.245 | 0.264 | 1.000 | 0.602 | 0.433 | 1.000 | 0.602 | 0.602 | 0.464 |
| **ssps588** | 8 | 3 | 2.462 | 0.974 | 0.625 | 0.594 | 0.633 | -0.053 | 0.497 | 0.395 | 1.000 | 0.497 | 0.497 | 0.717 |
| **ssps589** | 8 | 2 | 1.600 | 0.562 | 0.000 | 0.375 | 0.400 | 1.000 | 0.500 | 0.393 | 1.000 | 0.500 | 0.500 | 0.767 |
| **ssps590** | 8 | 3 | 2.723 | 1.043 | 0.625 | 0.633 | 0.675 | 0.012 | 0.497 | 0.395 | 1.000 | 0.497 | 0.497 | 0.717 |
| **ssps592** | 8 | 8 | 4.414 | 1.754 | 0.875 | 0.773 | 0.825 | -0.131 | 0.359 | 0.454 | 1.000 | 0.359 | 0.359 | 0.948 |
| **ssps593** | 8 | 2 | 1.600 | 0.562 | 0.250 | 0.375 | 0.400 | 0.333 | 0.469 | 0.408 | 1.000 | 0.469 | 0.469 | 0.625 |
| **ssps594** | 8 | 2 | 1.280 | 0.377 | 0.000 | 0.219 | 0.233 | 1.000 | 0.500 | 0.393 | 1.000 | 0.500 | 0.500 | 0.767 |
| **ssps595** | 8 | 2 | 1.280 | 0.377 | 0.000 | 0.219 | 0.233 | 1.000 | 0.500 | 0.393 | 1.000 | 0.500 | 0.500 | 0.767 |
| **ssps596** | 8 | 2 | 1.280 | 0.377 | 0.000 | 0.219 | 0.233 | 1.000 | 0.500 | 0.393 | 1.000 | 0.500 | 0.500 | 0.767 |
| **ssps597** | 8 | 5 | 2.909 | 1.249 | 0.875 | 0.656 | 0.700 | -0.333 | 0.469 | 0.408 | 1.000 | 0.469 | 0.469 | 0.865 |
| **ssps598** | 8 | 2 | 2.000 | 0.693 | 0.250 | 0.500 | 0.533 | 0.500 | 0.469 | 0.408 | 1.000 | 0.469 | 0.469 | 0.625 |
| **ssps600** | 8 | 2 | 1.280 | 0.377 | 0.250 | 0.219 | 0.233 | -0.143 | 0.469 | 0.408 | 1.000 | 0.469 | 0.469 | 0.625 |
| **ssps603** | 8 | 3 | 1.910 | 0.831 | 0.625 | 0.477 | 0.508 | -0.311 | 0.497 | 0.395 | 1.000 | 0.497 | 0.497 | 0.717 |
| **ssps605** | 7 | 2 | 2.000 | 0.693 | 0.143 | 0.500 | 0.538 | 0.714 | 0.594 | 0.436 | 1.000 | 0.594 | 0.594 | 0.679 |
| **ssps607** | 8 | 3 | 2.612 | 1.024 | 1.000 | 0.617 | 0.658 | -0.620 | 0.444 | 0.419 | 1.000 | 0.444 | 0.444 | 0.565 |
| **ssps608** | 8 | 2 | 1.133 | 0.234 | 0.125 | 0.117 | 0.125 | -0.067 | 0.492 | 0.397 | 1.000 | 0.492 | 0.492 | 0.700 |
| **ssps609** | 8 | 2 | 1.280 | 0.377 | 0.000 | 0.219 | 0.233 | 1.000 | 0.500 | 0.393 | 1.000 | 0.500 | 0.500 | 0.767 |
| **ssps610** | 8 | 2 | 1.600 | 0.562 | 0.000 | 0.375 | 0.400 | 1.000 | 0.500 | 0.393 | 1.000 | 0.500 | 0.500 | 0.767 |
| **ssps612** | 8 | 3 | 2.169 | 0.921 | 0.750 | 0.539 | 0.575 | -0.391 | 0.486 | 0.400 | 1.000 | 0.486 | 0.486 | 0.670 |
| **ssps613** | 7 | 7 | 5.765 | 1.847 | 0.143 | 0.827 | 0.890 | 0.827 | 0.428 | 0.479 | 1.000 | 0.428 | 0.428 | 0.485 |
| **ssps616** | 8 | 4 | 3.282 | 1.251 | 0.125 | 0.695 | 0.742 | 0.820 | 0.404 | 0.437 | 1.000 | 0.404 | 0.404 | 0.927 |
| **ssps619** | 8 | 3 | 1.293 | 0.463 | 0.250 | 0.227 | 0.242 | -0.103 | 0.486 | 0.400 | 1.000 | 0.486 | 0.486 | 0.837 |
| **ssps620** | 8 | 3 | 2.462 | 0.974 | 0.000 | 0.594 | 0.633 | 1.000 | 0.444 | 0.419 | 1.000 | 0.444 | 0.444 | 0.899 |
| **ssps621** | 8 | 7 | 4.571 | 1.721 | 0.375 | 0.781 | 0.833 | 0.520 | 0.316 | 0.468 | 1.000 | 0.316 | 0.316 | 0.964 |
| **ssps623** | 8 | 8 | 4.923 | 1.841 | 0.250 | 0.797 | 0.850 | 0.686 | 0.264 | 0.483 | 1.000 | 0.264 | 0.264 | 0.978 |
| **ssps624** | 6 | 2 | 1.600 | 0.562 | 0.167 | 0.375 | 0.409 | 0.556 | 0.648 | 0.445 | 1.000 | 0.648 | 0.648 | 0.661 |
| **ssps625** | 8 | 2 | 1.133 | 0.234 | 0.125 | 0.117 | 0.125 | -0.067 | 0.492 | 0.397 | 1.000 | 0.492 | 0.492 | 0.700 |
| **ssps626** | 8 | 2 | 2.000 | 0.693 | 0.000 | 0.500 | 0.533 | 1.000 | 0.500 | 0.393 | 1.000 | 0.500 | 0.500 | 0.767 |
| **ssps628** | 7 | 3 | 2.800 | 1.061 | 0.143 | 0.643 | 0.692 | 0.778 | 0.580 | 0.440 | 1.000 | 0.580 | 0.580 | 0.655 |
| **ssps629** | 8 | 2 | 1.280 | 0.377 | 0.000 | 0.219 | 0.233 | 1.000 | 0.500 | 0.393 | 1.000 | 0.500 | 0.500 | 0.767 |
| **ssps634** | 5 | 2 | 1.923 | 0.673 | 0.000 | 0.480 | 0.533 | 1.000 | 0.664 | 0.444 | 1.000 | 0.664 | 0.664 | 0.750 |
| **ssps636** | 6 | 2 | 1.385 | 0.451 | 0.000 | 0.278 | 0.303 | 1.000 | 0.656 | 0.444 | 1.000 | 0.656 | 0.656 | 0.607 |
| **ssps637** | 8 | 4 | 3.459 | 1.305 | 0.875 | 0.711 | 0.758 | -0.231 | 0.498 | 0.394 | 1.000 | 0.498 | 0.498 | 0.788 |
| **ssps638** | 8 | 3 | 2.327 | 0.947 | 0.250 | 0.570 | 0.608 | 0.562 | 0.486 | 0.413 | 1.000 | 0.486 | 0.486 | 0.837 |
| **ssps640** | 8 | 3 | 2.723 | 1.043 | 0.875 | 0.633 | 0.675 | -0.383 | 0.469 | 0.421 | 1.000 | 0.469 | 0.469 | 0.620 |
| **ssps644** | 8 | 2 | 1.133 | 0.234 | 0.125 | 0.117 | 0.125 | -0.067 | 0.492 | 0.410 | 1.000 | 0.492 | 0.492 | 0.700 |
| **ssps647** | 8 | 6 | 3.657 | 1.509 | 0.625 | 0.727 | 0.775 | 0.140 | 0.395 | 0.453 | 1.000 | 0.395 | 0.395 | 0.931 |
| **ssps648** | 8 | 3 | 1.684 | 0.736 | 0.000 | 0.406 | 0.433 | 1.000 | 0.444 | 0.432 | 1.000 | 0.444 | 0.444 | 0.899 |
| **ssps649** | 7 | 2 | 1.960 | 0.683 | 0.000 | 0.490 | 0.527 | 1.000 | 0.602 | 0.446 | 1.000 | 0.602 | 0.602 | 0.679 |
| **ssps650** | 8 | 2 | 1.280 | 0.377 | 0.000 | 0.219 | 0.233 | 1.000 | 0.500 | 0.406 | 1.000 | 0.500 | 0.500 | 0.767 |
| **ssps651** | 8 | 3 | 2.462 | 0.974 | 0.000 | 0.594 | 0.633 | 1.000 | 0.444 | 0.432 | 1.000 | 0.444 | 0.444 | 0.899 |
| **ssps652** | 8 | 2 | 1.882 | 0.662 | 0.500 | 0.469 | 0.500 | -0.067 | 0.375 | 0.460 | 1.000 | 0.375 | 0.375 | 0.450 |
| **ssps653** | 8 | 2 | 1.438 | 0.483 | 0.375 | 0.305 | 0.325 | -0.231 | 0.430 | 0.438 | 1.000 | 0.430 | 0.430 | 0.542 |
| **ssps654** | 8 | 2 | 1.882 | 0.662 | 0.000 | 0.469 | 0.500 | 1.000 | 0.500 | 0.406 | 1.000 | 0.500 | 0.500 | 0.767 |
| **ssps658** | 7 | 2 | 1.690 | 0.598 | 0.000 | 0.408 | 0.440 | 1.000 | 0.602 | 0.446 | 1.000 | 0.602 | 0.602 | 0.607 |
| **ssps663** | 8 | 2 | 1.280 | 0.377 | 0.000 | 0.219 | 0.233 | 1.000 | 0.500 | 0.406 | 1.000 | 0.500 | 0.500 | 0.767 |
| **ssps667** | 8 | 2 | 1.280 | 0.377 | 0.000 | 0.219 | 0.233 | 1.000 | 0.500 | 0.406 | 1.000 | 0.500 | 0.500 | 0.767 |
| **ssps669** | 8 | 2 | 1.969 | 0.685 | 0.125 | 0.492 | 0.525 | 0.746 | 0.492 | 0.410 | 1.000 | 0.492 | 0.492 | 0.700 |
| **ssps672** | 8 | 2 | 1.280 | 0.377 | 0.000 | 0.219 | 0.233 | 1.000 | 0.500 | 0.406 | 1.000 | 0.500 | 0.500 | 0.767 |
| **ssps673** | 7 | 2 | 1.324 | 0.410 | 0.000 | 0.245 | 0.264 | 1.000 | 0.602 | 0.446 | 1.000 | 0.602 | 0.602 | 0.464 |
| **ssps675** | 7 | 3 | 1.782 | 0.759 | 0.286 | 0.439 | 0.473 | 0.349 | 0.594 | 0.448 | 1.000 | 0.594 | 0.594 | 0.512 |
| **ssps676** | 5 | 2 | 1.724 | 0.611 | 0.200 | 0.420 | 0.467 | 0.524 | 0.656 | 0.456 | 1.000 | 0.656 | 0.656 | 0.714 |
| **ssps677** | 8 | 2 | 1.969 | 0.685 | 0.125 | 0.492 | 0.525 | 0.746 | 0.492 | 0.410 | 1.000 | 0.492 | 0.492 | 0.700 |
| **ssps681** | 8 | 2 | 1.280 | 0.377 | 0.000 | 0.219 | 0.233 | 1.000 | 0.500 | 0.406 | 1.000 | 0.500 | 0.500 | 0.767 |
| **ssps683** | 8 | 3 | 1.684 | 0.736 | 0.000 | 0.406 | 0.433 | 1.000 | 0.444 | 0.432 | 1.000 | 0.444 | 0.444 | 0.899 |
| **ssps685** | 8 | 2 | 1.753 | 0.621 | 0.125 | 0.430 | 0.458 | 0.709 | 0.492 | 0.410 | 1.000 | 0.492 | 0.492 | 0.700 |
| **ssps686** | 7 | 3 | 2.579 | 1.004 | 0.000 | 0.612 | 0.659 | 1.000 | 0.559 | 0.460 | 1.000 | 0.559 | 0.559 | 0.607 |
| **ssps689** | 8 | 2 | 2.000 | 0.693 | 0.000 | 0.500 | 0.533 | 1.000 | 0.500 | 0.406 | 1.000 | 0.500 | 0.500 | 0.767 |
| **ssps695** | 8 | 4 | 3.200 | 1.255 | 0.000 | 0.688 | 0.733 | 1.000 | 0.375 | 0.460 | 1.000 | 0.375 | 0.375 | 0.944 |
| **ssps696** | 8 | 2 | 1.882 | 0.662 | 0.000 | 0.469 | 0.500 | 1.000 | 0.500 | 0.406 | 1.000 | 0.500 | 0.500 | 0.767 |
| **ssps697** | 7 | 2 | 1.690 | 0.598 | 0.000 | 0.408 | 0.440 | 1.000 | 0.602 | 0.446 | 1.000 | 0.602 | 0.602 | 0.607 |
| **ssps700-1** | 4 | 3 | 2.667 | 1.040 | 0.000 | 0.625 | 0.714 | 1.000 | 0.611 | 0.458 | 1.000 | 0.611 | 0.611 | 0.690 |
| **ssps700-2** | 2 | 2 | 2.000 | 0.693 | 0.000 | 0.500 | 0.667 | 1.000 | 0.406 | 0.498 | 1.000 | 0.406 | 0.406 | 0.464 |
| **ssps701** | 8 | 3 | 2.723 | 1.043 | 0.625 | 0.633 | 0.675 | 0.012 | 0.497 | 0.410 | 1.000 | 0.497 | 0.497 | 0.717 |
| **ssps703** | 6 | 2 | 1.385 | 0.451 | 0.000 | 0.278 | 0.303 | 1.000 | 0.656 | 0.459 | 1.000 | 0.656 | 0.656 | 0.607 |
| **ssps705** | 8 | 2 | 1.882 | 0.662 | 0.000 | 0.469 | 0.500 | 1.000 | 0.500 | 0.409 | 1.000 | 0.500 | 0.500 | 0.767 |
| **ssps708** | 8 | 2 | 1.882 | 0.662 | 0.000 | 0.469 | 0.500 | 1.000 | 0.500 | 0.409 | 1.000 | 0.500 | 0.500 | 0.767 |
| **ssps710** | 8 | 4 | 1.707 | 0.822 | 0.125 | 0.414 | 0.442 | 0.698 | 0.404 | 0.452 | 1.000 | 0.404 | 0.404 | 0.927 |
| **ssps711** | 8 | 4 | 2.844 | 1.163 | 0.250 | 0.648 | 0.692 | 0.614 | 0.430 | 0.441 | 1.000 | 0.430 | 0.430 | 0.909 |
| **ssps713** | 8 | 5 | 3.657 | 1.424 | 0.625 | 0.727 | 0.775 | 0.140 | 0.439 | 0.437 | 1.000 | 0.439 | 0.439 | 0.900 |
| **ssps721** | 7 | 2 | 1.324 | 0.410 | 0.000 | 0.245 | 0.264 | 1.000 | 0.602 | 0.448 | 1.000 | 0.602 | 0.602 | 0.464 |
| **ssps722** | 8 | 2 | 1.600 | 0.562 | 0.000 | 0.375 | 0.400 | 1.000 | 0.500 | 0.409 | 1.000 | 0.500 | 0.500 | 0.767 |
| **ssps724** | 8 | 2 | 1.969 | 0.685 | 0.125 | 0.492 | 0.525 | 0.746 | 0.492 | 0.413 | 1.000 | 0.492 | 0.492 | 0.700 |
| **ssps726** | 8 | 3 | 2.462 | 0.974 | 0.000 | 0.594 | 0.633 | 1.000 | 0.444 | 0.435 | 1.000 | 0.444 | 0.444 | 0.899 |
| **ssps727** | 5 | 2 | 1.471 | 0.500 | 0.000 | 0.320 | 0.356 | 1.000 | 0.664 | 0.460 | 1.000 | 0.664 | 0.664 | 0.679 |
| **ssps728** | 8 | 2 | 2.000 | 0.693 | 0.000 | 0.500 | 0.533 | 1.000 | 0.500 | 0.409 | 1.000 | 0.500 | 0.500 | 0.767 |
| **ssps730** | 8 | 3 | 2.667 | 1.040 | 0.000 | 0.625 | 0.667 | 1.000 | 0.444 | 0.435 | 1.000 | 0.444 | 0.444 | 0.899 |
| **ssps732** | 7 | 2 | 1.324 | 0.410 | 0.000 | 0.245 | 0.264 | 1.000 | 0.602 | 0.448 | 1.000 | 0.602 | 0.602 | 0.464 |
| **ssps734** | 8 | 2 | 1.600 | 0.562 | 0.500 | 0.375 | 0.400 | -0.333 | 0.375 | 0.463 | 1.000 | 0.375 | 0.375 | 0.450 |
| **ssps736** | 8 | 4 | 2.909 | 1.213 | 0.250 | 0.656 | 0.700 | 0.619 | 0.430 | 0.441 | 1.000 | 0.430 | 0.430 | 0.909 |
| **ssps737** | 7 | 3 | 2.882 | 1.079 | 0.000 | 0.653 | 0.703 | 1.000 | 0.559 | 0.462 | 1.000 | 0.559 | 0.559 | 0.631 |
| **ssps741** | 7 | 4 | 3.769 | 1.352 | 0.000 | 0.735 | 0.791 | 1.000 | 0.506 | 0.477 | 1.000 | 0.506 | 0.506 | 0.571 |
| **ssps742** | 8 | 8 | 5.565 | 1.890 | 0.375 | 0.820 | 0.875 | 0.543 | 0.285 | 0.493 | 1.000 | 0.285 | 0.285 | 0.973 |
| **ssps743** | 8 | 2 | 1.882 | 0.662 | 0.000 | 0.469 | 0.500 | 1.000 | 0.500 | 0.409 | 1.000 | 0.500 | 0.500 | 0.767 |
| **ssps757** | 8 | 2 | 1.882 | 0.662 | 0.000 | 0.469 | 0.500 | 1.000 | 0.500 | 0.409 | 1.000 | 0.500 | 0.500 | 0.767 |
| **ssps758** | 8 | 2 | 1.882 | 0.662 | 0.000 | 0.469 | 0.500 | 1.000 | 0.500 | 0.409 | 1.000 | 0.500 | 0.500 | 0.767 |
| **ssps762** | 8 | 3 | 1.684 | 0.736 | 0.000 | 0.406 | 0.433 | 1.000 | 0.444 | 0.435 | 1.000 | 0.444 | 0.444 | 0.899 |
| **ssps766** | 8 | 2 | 1.280 | 0.377 | 0.000 | 0.219 | 0.233 | 1.000 | 0.500 | 0.409 | 1.000 | 0.500 | 0.500 | 0.767 |
| **ssps767** | 8 | 2 | 1.600 | 0.562 | 0.000 | 0.375 | 0.400 | 1.000 | 0.500 | 0.409 | 1.000 | 0.500 | 0.500 | 0.767 |
| **ssps768** | 8 | 5 | 4.000 | 1.494 | 0.000 | 0.750 | 0.800 | 1.000 | 0.320 | 0.482 | 1.000 | 0.320 | 0.320 | 0.964 |
| **Mean** | 7.587 | 2.952 | 2.197 | 0.819 | 0.173 | 0.475 | 0.510 | 0.664 | 0.491 | 0.439 | 1.000 | 0.491 | 0.491 | 0.731 |
| **SE** | 0.053 | 0.077 | 0.058 | 0.023 | 0.016 | 0.011 | 0.012 | 0.028 | 0.079 | 0.046 | 0.000 | 0.079 | 0.079 | 0.149 |

***Na*** = No. of Different Alleles; ***Ne*** = No. of Effective Alleles; ***I*** = Shannon's Information Index; ***Ho*** = Observed Heterozygosity; ***He*** = Expected Heterozygosity; ***uHe*** = Unbiased Expected Heterozygosity; ***F*** = Fixation Index; ***H*** = expected heterozygosity; ***PIC*** = polymorphism information content; ***E*** = effective multiplex ratio; ***Hav*** = mean heterozygosity; ***MI*** = marker index; ***D*** = discriminating power.
