# Supplementary figures and images for: Development of a Large Gene-Associated SSR Marker Set and in-Depth Genetic Characterization in Scarlet Sage
Source: Front Genet. 2020 May 21;11:504. doi: 10.3389/fgene.2020.00504 (PMC7253628; doi:10.3389/fgene.2020.00504)

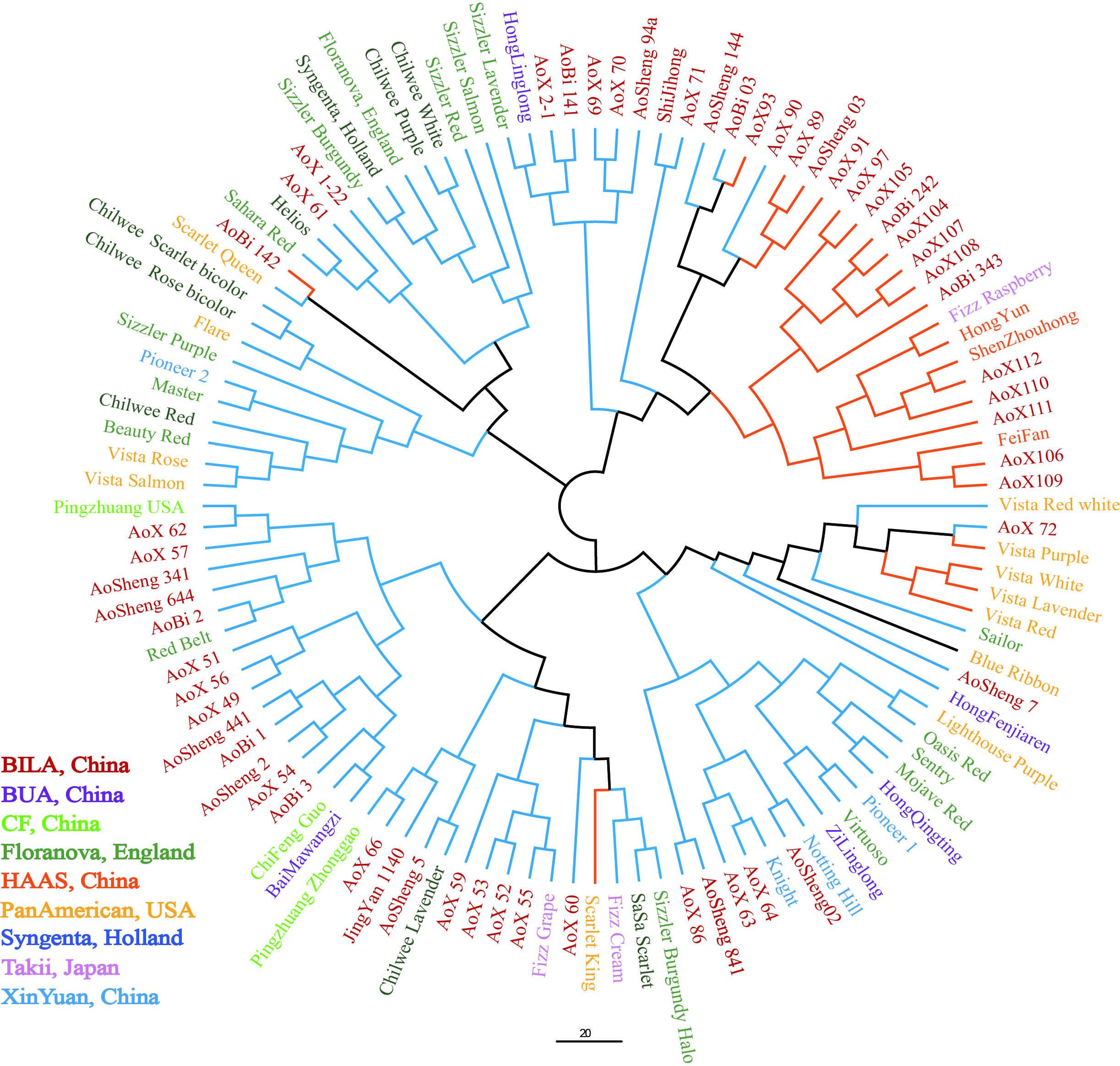

Supplement: Supplementary file 2 [file Image_1.JPEG]
